# [12]aneN_3_-based multifunctional compounds as fluorescent probes and nucleic acids delivering agents

**DOI:** 10.1080/10717544.2019.1704943

**Published:** 2019-12-20

**Authors:** Yong-Guang Gao, Shu-Yuan Huangfu, Suryaji Patil, Quan Tang, Wan Sun, Yu Li, Zhong-Lin Lu, Airong Qian

**Affiliations:** a Lab for Bone Metabolism, Key Lab for Space Biosciences and Biotechnology, School of Life Sciences, Northwestern Polytechnical University, Xi’an, Shaanxi, China;; b Key Laboratory of Theoretical and Computational Photochemistry, Ministry of Education, College of Chemistry, Beijing Normal University, Beijing, China

**Keywords:** [12]aneN3, 1,8-naphthalimide, Cu2^+^, fluorescent probe, lysosome, gene delivery

## Abstract

A series of multifunctional compounds (MFCs) **1a**–**1e** based on 1,8-naphthalimide and [12]aneN_3_ building blocks were designed and synthesized. They were used as not only fluorescent probes for recognition of Cu^2+^ ions but also as non-viral gene vectors for DNA and RNA delivery. Furthermore, their complexes with Cu^2+^ (**1**-Cu) could also selectively stain lysosome in HeLa cells. In order to achieve high performance multifunctional materials, structure-performance relationship of MFCs **1a–1e** was studied. It was found that MFCs **1a**–**1e** exhibited highly selective fluorescence turn-off for Cu^2+^, without interference by other metal ions in aqueous solution. The fluorescence emission of **1a–1e** was quenched by a factor of 10-fold, 47-fold, 6-fold, 64-fold, and 15-fold respectively in the presence of Cu^2+^ ions. Due to high sensitivity, good water solubility, and low cytotoxicity, MFCs **1a–1d** were successfully applied in the recognition of Cu^2+^ and selectively staining lysosome in HeLa cells. Most importantly, MFCs **1a** and **1b** had excellent HeLa cell selectivity in RNA delivery, and their performances were far better than lipofectamine 2000 and 25 kDa PEI.

## 1. Introduction

In recent years, considerable efforts have been paid to develop high performance probes for detecting transition-metal ions or monitoring cellular pH changes (Richter et al., 2008; Wu et al., 2014; Cotruvo et al., 2015; Yue et al., 2017; Alam et al., 2018; Yang et al., 2018). Cu^2+^ as the third most abundant of the essential nutrients plays an important role in many neurological diseases, including Menke’s disease and prion disease (Karr & Szalai, 2007; Zhang et al., 2009; Tewari, 2010; Eshak et al., 2018; Siotto & Squitti, 2018). Furthermore, abnormal cellular pH values are also related with some common diseases such as Alzheimer’s diseases and cancer (Izumi et al., 2003; Junyan & Kevin, 2010). Therefore, sensing of Cu^2+^ concentration level and monitoring of pH change would give us important information for disease diagnosis. The organelles in eukaryotic cells participate in life activities within various pH ranges. For example, the pH value of lysosome lumen ranges from 4.0 to 6.0, while the cytoplasm is about 7.2. Therefore, many fluorescence probes for the recognition of lysosome or detection of Cu^2+^ have been reported (Jeppe et al., 2013; Khan et al., 2015; Behnood et al., 2016; Gu et al., 2018; Li et al., 2018; Shi et al., 2018). However, the same compounds used for recognition of both Cu^2+^ and lysosome are relatively rare. The structure-property relationship of these materials in recognition systems has been seldom investigated (Shuqi et al., 2011; Zhu et al., 2011; Hao et al., 2012). This relationship is usually very important for the designation of high performance fluorescence probes.

It is well known that the development of safe and efficient gene delivery systems is important for gene therapy (Verma & Somia, 1997; Dunbar et al., 2018; Peng, 2018). Viral vectors such as retroviruses and adenoviruses are significantly more efficient for *in vitro* and *in vivo* gene delivery (Lundstrom, 1995; Marshall, 1999; Bloom et al., 2018). However, their clinical applications are limited due to high immunogenicity and limited loading capacity (Thomas et al., 2003; Luo et al., 2015). In contrast, non-viral vectors such as cationic lipids, cationic polymers, and organic functional molecules have received more and more attention due to their easy preparation, low immunogenicity, and good biodegradability (Posocco et al., 2013; Yi et al., 2014; Ramamoorth & Narvekar, 2015; Yang et al., 2015; Hu et al., 2016; Wang et al., 2016; Hardee et al., 2017; Wang et al., 2017; Luo et al., 2018; Tan et al., 2018; Li et al., 2019; Wang et al., 2019). Among these non-viral vectors, organic functional molecules such as organic fluorescent compounds not only show high transfection efficiency but also exhibit good tracking ability (You et al., 2014; Gao et al., 2015). However, the usage of these organic functional molecules both in non-viral vectors and Cu^2+^/pH probes has never been reported.

In recent years, we have developed a series of small organic molecules containing rigid fluorescent moieties (naphthalimide, BODIPY, and coumarin) and [12]aneN_3_ units (Yue et al., 2015; Zhang et al., 2015; Gao et al., 2016; 2016; 2016; Ding et al., 2017). It was found that [12]aneN_3_ unite exhibited multiple functions such as good water solubility, strong coordination ability with metal ions and excellent DNA binding ability. Therefore, some were used as non-viral vectors to deliver DNA into target cells (Yue et al., 2015; Gao et al., 2016; Ding et al., 2017) and some were applied in fluorescent probes for recognition of metal ions or small bioactive molecules (Zhang et al., 2015; Gao et al., 2016; 2016). However, a functional molecule serving simultaneously as a gene vector as well as the fluorescence probe has not been investigated so far. Furthermore, as the non-viral gene vectors, we mainly focused on DNA delivery, while RNA delivery mediated by these materials has never been investigated.

**Scheme 1.**
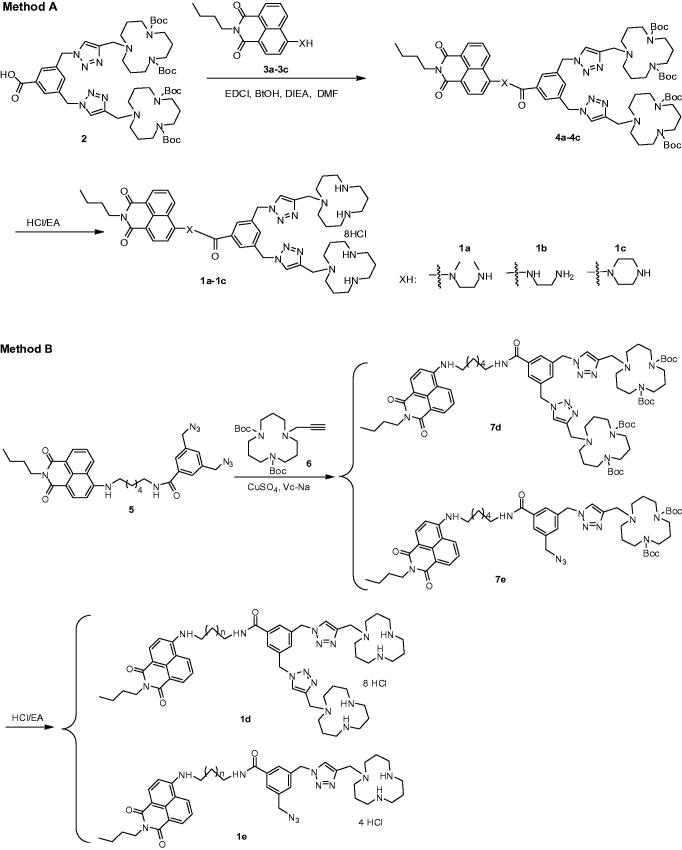
Synthetic routes of the multifunctional compounds **1a–1e**. X represents different diamine groups, **1a**: N(CH_3_)CH_2_CH_2_N(CH_3_), **1b**: N(CH_2_CH_2_)_2_N, **1c**: NHCH_2_CH_2_NH.

In this study, we designed and synthesized a series of multifunctional compounds (MFCs) **1a**–**1e** (Scheme 1). They consist of three parts: fluorophore unit naphthalimide, linker benzoyl amine derivative and hydrophilic units triazole and [12]aneN_3_. The main difference lies in the length of linker or the number of hydrophilic units. **1a**, **1b**, **1c**, and **1d** contain different linkers: *N*,*N′*- dimethyl-1,2-ethanediamine, ethylenediamine, piperazine and 1,6-hexylenediamine, respectively. Compared to other compounds, **1e** linked with 1,6-hexylenediamine only contain one triazole and one [12]aneN_3_ unit. These structural differences will be beneficial to structure-property relationship investigation. Therefore, their performance on recognition of Cu^2+^ ions and lysosome as well as gene delivery was systematically investigated. The results show that these multifunctional compounds can not only serve as fluorescent probes for detection of Cu^2+^ ions and lysosome but also serve as non-viral vectors for DNA and RNA delivery. Most importantly, the structure-property relationship investigation will give further insight to design and synthesize new types of high performance multifunctional compounds.

## 2. Materials and methods

### 2.1. Materials and instruments

The solvents used for reaction such as tetrahydrofuran (THF) and *N*,*N*-dimethylformamide (DMF) were purified by distillation before use. The solvents used for purification such as dichloromethane (DCM), methanol (MeOH), petroleum ether (PE) and ethyl acetate (EA) were directly used without any purification. 1-hydroxybenzotriazole hydrate (HOBt), copper sulfate (CuSO_4_), diisopropylethylamine (DIEA), 1-Ethyl-3-(3-dimethylaminopropyl)carbodiimide hydrochloride (EDCI) and sodium ascorbate (Vc-Na) were purchased from Beijing Ouhe Technology Co. Ltd. (Beijing, China). 3,5-bis((4-((5,9-bis(*tert*-butoxycarbonyl)- 1,5,9-triazacyclododecan-1-yl)methyl) -1*H*-1,2,3-triazol-1-yl)methyl) benzoic acid (compound **2**), 4-amino-1,8-naphthalimide derivatives (compounds **3a**, **3b**, and **3c**), 3,5- bis(azidomethyl)-*N*-(6-(2-butyl-1,3-dioxo-2,3-dihydro-1*H*-benzo[*de*]isoquinolin-6-ylamino)hexyl) benzamide (compound **5**) and ditert-butyl 9-(prop-2-ynyl)-1,5,9-triazacyclododecane- 1,5-dicarboxylate (compound **6**) were prepared according to the literature (Gao et al., 2015; 2016; 2018).

^1^H NMR and ^13^C NMR spectra were collected using a Bruker Avance spectrometer. Mass spectra were obtained on a Waters Quattro Mocro spectrometer and high resolution mass spectra were acquired on a Waters LCT Premier XE spectrometer. The infrared spectra were acquired on a Nicolet 380 spectrometer. Fluorescence spectra were measured on a Hitachi F-4500 Fluorescence Spectrophotometer. Hydrodynamic diameters and Zeta Potentials were collected using a Nano-ZS 3600 ZetaPlus Partical Size and Zeta Potential Analyzer.

### 2.2. Synthesis of multifunctional compounds

#### 2.2.1. Synthesis of compounds 4a–4c

General procedure: 3,5-bis((4-((5,9-bis(*tert*-butoxycarbonyl)-1,5,9-triazacyclododecan-1-yl) methyl)-1*H*-1,2,3-triazol-1-yl)methyl)benzoic acid **2** (1.0 mmol), EDCI (1.1 mmol), DIEA (2.0 mmol) and HOBt (1.1 mmol) were added in anhydrous *N*,*N*-dimethylformamide (20 mL) under argon atmosphere and stirred for 0.5 h. 4-Amino-1,8-naphthalimide derivatives **3a**/**3b**/**3c** (1.0 mmol) was then added slowly to the above reaction mixture, and continued to stir for 12–24 h. After the completion of reaction (monitor by TCL), ice water (50 mL) was added to the mixture. The aqueous phase was extracted with ethyl acetate. The combined organic phase was dried over anhydrous Na_2_SO_4_, filtered, and the solvent was evaporated under reduced pressure. The crude material was purified by column chromatography (DCM/MeOH = 10/1) to give products **4a–4c** as yellow solid. The detailed analytical data is listed in the Supplementary Information.

#### 2.2.2. Synthesis of compounds 7d and 7e

3,5-bis(Azidomethyl)-*N*-(6-(2-butyl-1,3-dioxo-2,3-dihydro-1*H*-benzo[*de*]isoquinolin-6-ylamino)hexyl)benzamide **5** (1.0 mmol) and ditert-butyl 9-(prop-2-ynyl)-1,5,9-triazacyclododecane- 1,5-dicarboxylate **6** (2.0 mmol) were dissolved in the mixture of tetrahydrofuran (10 mL) and water (5 mL). Sodium ascorbate (0.1 mmol) and copper sulfate (0.05 mmol) were added dropwise in 1 mL of water. The mixture was stirred for 8 h at room temperature. After completion of reaction, the solvent was removed by reduced pressure. The solid was washed with CH_2_Cl_2_ (3 × 20 mL), the combined organic layer was evaporated under reduced pressure. The crude material was purified by column chromatography (DCM/MeOH = 10/1) to give products **7d** and **7e** as yellow solid. The detailed analytical data are listed in the Supplementary Information.

#### 2.2.3. Synthesis of multifunctional organic compounds 1a–1e

General procedure: compound **4a**/**4b**/**4c**/**7d**/**7e** (0.1 mmol) was added to a saturated HCl/ethyl acetate solution (5 mL). After string for 1 h, the resulting suspension was filtrated, washed with ether 3 times and dried in vacuum at 60 °C for 24 h to give MFCs **1a**–**1e** as yellow solid.

**1a**: 95%; ^1^H NMR (400 MHz, D_2_O) δ 8.07 - 7.77 (m, 4 H), 7.66 - 7.49 (m, 1 H), 7.31 - 7.17 (m, 1 H), 7.11 - 6.98 (m, 1 H), 6.81 - 6.41 (m, 3 H), 5.30 (s, 2 H), 5.11 (s, 2 H), 4.18 - 4.15 (m, 4 H), 3.73 - 3.42 (m, 5 H), 3.33 - 2.87 (m, 28 H), 2.77 (s, 1 H), 2.36 - 1.91 (m, 14 H), 1.31 (d, *J* = 6.9 Hz, 1 H), 1.10 (s, 3 H), 0.71 - 0.66 (m, 3 H); ^13^C NMR (101 MHz, D_2_O) δ 176.24, 171.88, 171.37, 164.42, 163.66, 154.80, 136.46, 136.23, 135.92, 132.44, 131.03, 129.45, 128.75, 127.95, 126.74, 126.52, 125.01, 123.96, 123.15, 121.04, 114.39, 113.95, 113.04, 112.66, 73.17, 60.25, 58.07, 57.47, 53.26, 53.03, 48.92, 48.24, 47.47, 45.46, 42.35, 42.05, 41.16, 39.98, 37.92, 33.69, 29.49, 20.61, 20.19, 19.81, 17.97, 17.04, 13.27; IR (KBr, cm^−1^): 3428.01, 2959.04, 2650.00, 1684.94, 1641.57, 1581.93 1451.81, 1384.04, 1356.93, 1234.94, 1053.31, 790.36; HR-MS (ES^+^) calcd. For C_53_H_77_N_15_O_3_ (M + H)^+^: 972.6334, found 972.6427.

**1b**: 93%; ^1^H NMR (400 MHz, D_2_O) δ 7.99 (d, *J* = 10.7 Hz, 2 H), 7.44 (s, 2 H), 7.35 - 6.95 (m, 4 H), 6.70 - 6.46 (m, 1 H), 5.81 - 5.78 (m, 1 H), 5.40 (s, 4 H), 3.94 (s, 4 H), 3.53 - 2.75 (m, 30 H), 2.06 (s, 4 H), 1.92 (s, 12 H), 1.04 (s, 2 H), 0.94 - 0.82 (m, 2 H), 0.56 (t, *J* = 7.1 Hz, 3 H); ^13^C NMR (101 MHz, D_2_O) δ 176.38, 168.98, 164.30, 163.40, 149.85, 136.44, 136.16, 134.84, 133.60, 131.38, 130.12, 128.04, 127.56, 127.30, 123.44, 119.56, 118.24, 106.46, 103.17, 57.48, 53.32, 48.91, 47.49, 42.82, 42.04, 41.14, 39.74, 38.81, 29.56, 20.54, 20.16, 19.82, 17.97, 16.95, 13.20; IR (KBr, cm^−1^): 3425.30, 2956.33, 2650.00, 1682.23, 1638.86, 1581.93 1546.69, 1392.17, 1356.93, 1234.07, 1121.08, 1058.73, 776.81; HR-MS (ES^+^) calcd. For C_53_H_73_N_15_O_3_ (M + H)^+^: 944.6021, found 944.6103.

**1c**: 97%; ^1^H NMR (400 MHz, D_2_O) δ 8.25 (s, 2 H), 7.72 - 7.39 (m, 3 H), 7.43 - 7.30 (m, 3 H), 7.04 (s, 1 H), 6.50 (s, 1 H), 5.62 (s, 4 H), 4.40 (s, 4 H), 3.76 (s, 2 H), 3.45 - 3.25 (m, 28 H), 2.88 - 2.76 (m, 4 H), 2.14 - 2.12 (m, 12 H), 1.18 - 1.06 (m, 4 H), 0.67 (s, 3 H); ^13^C NMR (101 MHz, D_2_O) δ 170.32, 163.70, 163.25, 154.49, 136.23, 136.04, 135.65, 129.45, 128.20, 126.83, 124.13, 120.87, 114.51, 53.18, 48.97, 47.09, 41.73, 40.85, 29.48, 19.89, 17.66 (s, 8 H), 13.25 (s, 3 H); IR (KBr, cm^−1^): 3428.01, 2956.33, 2753.01, 1693.07, 1649.70, 1587.35, 1454.52, 1386.75, 1237.65, 1053.31, 787.65; ESI-MS: HR-MS (ES^+^) calcd. For C_53_H_75_N_15_O_3_ (M + H)^+^: 970.6177, found 970.6231.

**1d**：88%; ^1^H NMR (400 MHz, D_2_O) δ 7.77 (s, 2 H), 7.51 (d, *J* = 6.9 Hz, 1 H), 7.47 (d, *J* = 7.5 Hz, 1 H), 7.28 (s, 2 H), 7.22 (d, *J* = 8.1 Hz, 1 H), 7.09 (s, 1 H), 6.85 (t, *J* = 7.1 Hz, 1 H), 5.76 (d, *J* = 6.9 Hz, 1 H), 5.26 (s, 4 H), 3.64 (s, 4 H), 3.42 (s, 2 H), 3.16 - 2.99 (m, 18 H), 2.84 (s, 2 H), 2.61 (s, 8 H), 2.02 (s, 4 H), 1.77 (s, 8 H), 1.40 (s, 4 H), 1.19 (s, 6 H), 1.05 - 0.98 (m, 2 H), 0.62 (t, *J* = 7.1 Hz, 3 H); ^13^C NMR (101 MHz, D_2_O) δ 168.23, 164.70, 163.71, 150.41, 138.48, 136.03, 135.34, 134.07, 130.95, 130.56, 128.19, 127.78, 126.97, 126.96, 123.69, 119.90, 118.71, 106.06, 103.34, 53.24, 48.76, 47.31, 43.02, 42.76, 41.77, 40.70, 39.92, 29.76, 28.52, 27.67, 26.22, 26.07, 20.00, 19.94, 19.00, 13.30; IR (KBr, cm^−1^): 3409.04, 2929.22, 2853.31, 2750.30, 1676.81, 1638.86, 1579.22, 1543.98, 1457.23, 1432.83, 1392.17, 1356.93, 1245.78, 1167.17, 1115.66, 1053.31, 782.23; HR-MS (ES^+^) calcd. For C_55_H_81_N_15_O_3_ (M + H)^+^: 1000.6647, found 1000.6703.

**1e**：71%;^1^H NMR (400 MHz, CD_3_SOCD_3_) δ 11.23 (s, 1 H), 9.52 (s, 4 H), 8.76 (d, *J* = 8.3 Hz, 1 H), 8.64 (s, 1 H), 8.51 (s, 1 H), 8.42 (d, *J* = 6.9 Hz, 1 H), 8.28 - 8.23 (m, 1 H), 7.80 (d, *J* = 8.7 Hz, 2 H), 7.66 (t, *J* = 7.6 Hz, 1 H), 7.46 - 7.43 (m, 1 H), 6.76 (d, *J* = 8.4 Hz, 1 H), 5.74 - 5.67 (m, 2 H), 4.53 (s, 2 H), 4.42 (s, 2 H), 4.20 (s, 6 H), 4.04 - 3.97 (m, 2 H), 3.45 (s, 2 H), 3.26 - 3.16 (m, 8 H), 2.16 - 2.14 (m, 4 H), 1.87 - 1.70 (m, 4 H), 1.64 - 1.26 (m, 12 H), 0.91 (t, *J* = 7.3 Hz, 3 H); ^13^C NMR (101 MHz, DMSO) δ 165.74, 164.19, 163.35, 151.13, 137.05, 136.09, 134.71, 131.06, 129.89, 129.23, 127.24, 127.09, 124.59, 122.24, 120.57, 107.82, 104.15, 53.58, 43.19, 30.27, 29.51, 28.22, 26.82, 26.73, 20.27, 14.19; IR (KBr, cm^−1^): 3400.90, 2923.80, 2853.31, 2099.70, 1679.52, 1638.86, 1579.22, 1546.69, 1462.65, 1397.59, 1384.04, 1354.22, 1272.89, 1237.65, 1115.66, 1056.02, 782.23; HR-MS (ES^+^) calcd. For C_43_H_58_N_12_O_3_ (M + H)^+^: 791.4755, found 791.4841.

### 2.3. UV or fluorescent spectral measurements

The stock solutions of multifunctional organic compounds (MFCs) **1a**–**1e** (1 mM) and metal ions (10 mM) were prepared in tri-distilled water, and stored at 4 °C for use. The metal salts were AgClO_4_·H_2_O, Ca(ClO_4_)_2_·4H_2_O, Cd(ClO_4_)3·6H_2_O, Co(ClO_4_)_2_·6H_2_O, Fe(ClO_4_)_2_, 3H_2_O, Hg(ClO_4_)_2_·3H_2_O, KClO_4_, LiClO_4_·3H_2_O, Mg(ClO_4_)_2_·6H_2_O, NaClO_4_·H_2_O, Ni(ClO_4_)_2_·6H_2_O, Pb(ClO_4_)_2_·3H_2_O,Zn(ClO_4_)_2_·6H_2_O and Cu(ClO_4_)_2_·6H_2_O. The fluorescence emission and ultraviolet absorption experiments were carried out in Tris-HCl buffer (1 mM, pH 7.2). Test solution was prepared by placing MFCs **1a**–**1e**, Tris-HCl buffer solution and an appropriate volume of each analyte into 3 mL of cuvette. After equilibration for 2 min, an ultraviolet absorption and fluorescence emission spectrum were recorded at 25 °C.

### 2.4. Cell culture and fluorescence imaging

Cell culture: HeLa cells ((a human cervical carcinoma cell line) were cultured with DMEM containing 100 units/mL penicillin sulfate and streptomycin, medium supplemented with 10% fetal bovine serum at 37 °C under 5% CO_2_ for 24 h.

Cell imaging (Cu^2+^ recognition): cells were seeded in glass bottom cell culture dish (8 × 10^4^ cells), and incubated with 20 μM of MFCs **1a**–**1e** for 0.5 h. After incubation, the cells were washed with PBS 4 times and imaged under a fluorescence microscope. Then 10 equivalent of Cu(ClO_4_)_2_ was added to the cells, and fluorescence images were taken one time per 15 minutes under a LEICA DMI8 Inverted Fluorescence Microscope. Fluorescence images were obtained using a 10 × objective lens. Cell images were processed and analyzed using the Image-Pro Plus software. Three repeats were conducted for each sample.

Lysosome imaging: The cells were seeded in 24-well plates at 8 × 10^4^ cells per well and grew for 24 h. After removing the medium, **1a–1e**/Cu complexes (20 μM) were added and incubated for 30 minutes in DMEM medium. Then the medium was replaced with fresh medium containing Lyso-Tracker Red (10 μM) and incubated for another 30 minutes. The samples were then imaged by a Inverted Fluorescence Microscope after washing with cell culture medium. Lyso-Tracker Red images were observed in the red channel, **1a–1e**/Cu images were observed in the green channel with a 20 × objective lens. Cell images were processed and analyzed using the Image-Pro Plus software. Three repeats were conducted for each sample.

### 2.5. Dynamic light scattering (DLS) and scanning electron microscope (SEM)

The complexes of MFCs **1a**–**1e** with DNA or RNA were prepared by adding 0.9 μL of pUC18 DNA (200 μg/mL) or 0.7 μL of siRNA (264 μg/mL, 5′-UUCUCCGAACGUGUCACGUTT-3′) to the appropriate volume of the stock solutions of **1a**–**1e**. Then the complex solution was vortexed for 30 s and then diluted up to 0.5 mL by tri-distilled water. The zeta potentials and the hydrodynamic diameters were measured using Nano-ZS 3600 ZetaPlus Partical Size and Zeta Potential Analyzer. The complexes of **1b** (20 µM) with RNA or DNA were added dropwise to the silicon slice. The slice was then dried in a vacuum oven at room temperature for 8 h before observation.

### 2.6. Cell uptake of 1a–1e/RNA (DNA) complexes

The cellular uptake of the complexes of MFCs **1a**–**1e** with Cy5-labeled siRNA or dsDNA was obtained by fluorescence microscopy. HeLa, HepG2 and U2Os cells were cultured with DMEM medium, and MC3T3-E1 cells were cultured with α-MEM medium. The cells were seeded in 24-well plates at 8 × 10^4^ cells per well and grew for 24 h. After washing with DMEM, the cells were incubated with freshly prepared complexes of MFCs **1a**–**1e** with Cy5-RNA (9 μg/mL) or Cy5-DNA (9 μg/mL) and the controls (500 µL). After 4 h of incubation, the cells were washed for 6 times with PBS buffer. Cy5-labeled RNA or DNA was observed in the red channel, and MFCs **1a**–**1e** was observed in the green channel using a Fluorescence Microscope with a 10 × objective lens.

### 2.7. Cytotoxicity experiment

The cytotoxicity of **1a**–**1e**/RNA or **1a**–**1e**/DNA complexes toward different cell lines (HeLa, U2Os, HepG2, and MC3T3-E1 cells) was tested by MTT assays. The cells were seeded on 96-well plates at densities of 5 × 10^3^ cells in 100 μL α-MEM/DMEM medium. After culture for 24 h, the complexes (**1a**–**1e**/RNA or **1a**–**1e**/DNA) were added to cells at various concentrations (10 μM, 15 μM, 20 μM, and 25 μM). After incubation for 4 h, the medium was replaced with 200 μL of fresh medium containing 10% FBS and cells were cultured for another 48 h. Subsequently, 20 µL of MTT (5 mg/mL) solution in PBS was added to each well for additional 4 h incubation. The MTT medium was replaced by 200 µL of DMSO. The absorbance was measured using a microplate spectrophotometer at a wavelength of 490 nm. The cells treated without any complexes were used as controls. The relative viability of the cells was calculated based on the data of five parallel tests by comparing to the controls.

## 3. Results and discussion

### 3.1. Synthesis of the MFCs 1a–1e

In this report, five multifunctional compounds were synthesized by two methods (Scheme 1). The compounds linked with short diamine chains (**1a, 1b,** and **1c**, XH with *N*,*N′*-Dimethylethylenediamine, ethylenediamine, and piperazine respectively) were easily synthesized according to Method A, and the synthetic route consisted of two steps. Compound **4** was synthesized through acylation of **3** with compound **2** in the presence of (1-ethyl-3-(3-dimethylaminopropyl)carbodiimide hydrochloride) (EDC·HCl) and *N*-hydroxybenzotriazole (HOBt). The title compounds **1a, 1b,** and **1c** were prepared by de-protection of **4** under acidic condition. The compounds linked with C-6 alky chain **(1d** with two [12]aneN_3_ units and **1e** with one [12]aneN_3_ unit**)** were synthesized according to Method B. Reaction of **5** with propargyl [12]aneN_3_ **6** through click cycloaddition yielded key intermediates **7d** and **7e**. Further de-protection of Boc under acidic condition resulted in title compounds **1d** and **1e**. All new compounds were fully characterized by ^1^H NMR, ^13^C NMR, IR and MS.

### 3.2. Spectroscopic properties of 1a–1e

**Figure 1.**
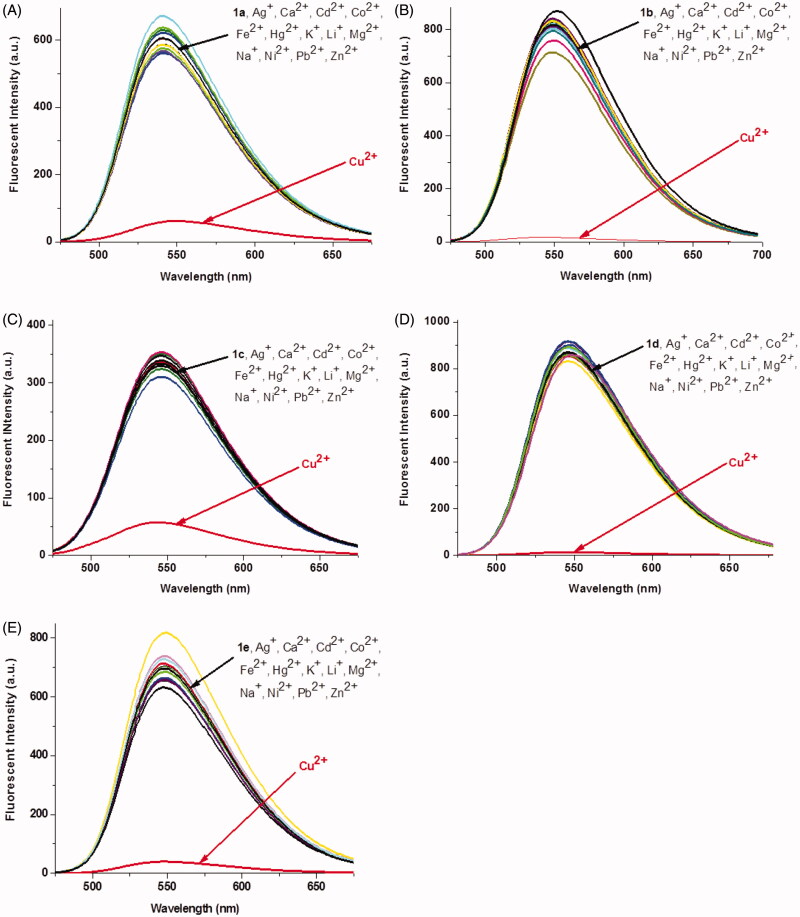
Fluorescence changes of **1a–1e** (1 × 10^−5^M, Tris-HCl buffer, 1 mM) upon the addition of 5 equiv of various metal ions. The excited wavelength of **1a**–**1e**: 465.0 nm, 465.0 nm, 410.0 nm, 465.0 nm, and 465.0 nm, respectively.

First, the absorption and fluorescence properties of **1a–1e** were measured in the water-Tris-HCl buffer (1 mM, pH 7.2). Each compound exhibited a broad absorption peak in the range of 300–500 nm (Figure S1(A)). Compared to other compounds, the maximum absorption wavelength of **1c** linked with piperazine shifted toward shorter wavelength, which may be caused by the larger steric hindrance. The fluorescence spectra of **1a–1e** showed that there are no obvious changes in fluorescence emission spectra, the maximum emission wavelength of **1a–1e** appeared at 542 nm, 550 nm, 546 nm, 546 nm, and 546 nm, respectively (Figure S1(B)). Then, the fluorescence spectra changes of **1a–1e** were monitored in the presence of different metal ions (Ag^+^, Ca^2+^, Cd^2+^, Co^2+^, Fe^2+^, Hg^2+^, K^+^, Li^+^, Mg^2+^, Na^+^, Ni^2+^, Pb^2+^, Zn^2+^, and Cu^2+^). As shown in Figure 1, these five materials exhibited high selectivity toward Cu^2+^ in aqueous solution. The fluorescence intensities of **1a**–**1e** were decreased in different degrees after the addition of Cu^2+^, about 10-fold, 47-fold, 6-fold, 64-fold, and 15-fold, respectively (Table S1). However, the addition of the other metal ions produced a negligible fluorescence change (Figure 1). These results suggest that the linker between the naphthalimide unit and the benzoic acid unit has a great influence on the fluorescence recognition of copper ions. The probe linked with a longer alkyl chain exhibited stronger recognition ability to copper ions (**1d **>** 1b**). When a bulk group such as *N*,*N′*-dimethyl-1,2-ethanediamine or piperazine was used as a linker, the ability to recognize copper ions was decreased dramatically (**1b **>** 1a**, **1b **>** 1c**), which may be induced by the steric hindrance effect of substituent group. Besides, the fluorescence changes were also affected by the number of triazole and [12]aneN_3_. The probes with two triazole and [12]aneN_3_ units showed stronger Cu^2+^ recognition ability than these with one unit (**1d **>** 1e**). Furthermore, the metal ions competition experiments were also carried out. As shown in Figure S2, the fluorescence emission of compound **1b** was nearly quenched after addition of Cu^2+^ in the presence of other interfering metal ions (except Hg^2+^), which exhibited its good ability for Cu^2+^ recognition.

**Figure 2.**
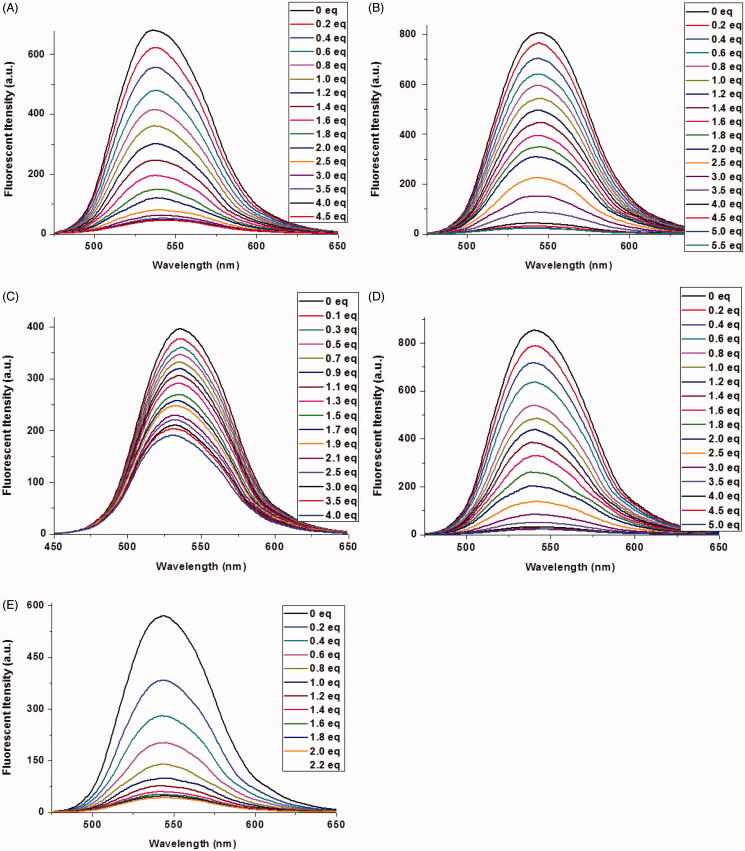
Fluorescence spectra of **1a**–**1e** (10 μM) upon the addition of Cu^2+^ in aqueous solution (1 mM Tris-HCl, pH 7.2). The excited wavelength of **1a**–**1e**: 465.0 nm, 465.0 nm, 410.0 nm, 465.0 nm, and 465.0 nm, respectively.

The fluorescence titrations of **1a–1e** with Cu^2+^ were performed in 1 mM Tris-HCl buffer solution. As shown in Figure 2, the addition of Cu^2+^ to **1a**–**1e** led to a large quenching effect with the maximum emission at 542 nm, 550 nm, 546 nm, 546 nm, and 546 nm, respectively. A good linear relationship was found between Cu^2+^ concentration and fluorescent intensities of **1a–1e** (**1a–1d**: 0–2.0 equiv. of Cu^2+^, **1e**: 0–1.0 equiv. of Cu^2+^). According the formula (LOD = 3σ/k), the limit of detection of **1a–1e** was calculated to be 1.21 × 10^−8^M, 7.48 × 10^−9^M, 4.40 × 10^−8^M, 1.23 × 10^−9^M and 2.36 × 10^−9^M, respectively (Figure S3). These results indicated that reducing steric hindrance or increasing the length of alkyl chain between naphthalimide unit and benzoic acid unit was beneficial to lower the detection limits of Cu^2+^. It should be noticed that the limits of detection toward Cu^2+^by these probes (except **1c**) are lower than our previous reports, and far lower than the limit of Cu^2+^ ions in drinking water (Zhong et al., 2013; Zhang et al., 2015).

To confirm the stoichiometry between **1** and Cu^2+^, Job’s plot analyses were carried out (Figure S4).

The results indicated that **1a–1d** and Cu^2+^ formed 1:2 stoichiometrical complexes with association constants of Kassoc = 8.2 × 10^9^ M^−2^, 2.2 × 10^10^ M^−2^, 1.1 × 10^10^ M^−2^, and 4.4 × 10^10^ M^−2^, respectively. Compound **1e** and Cu^2+^ formed a 1:1 stoichiometrical complex with association constant of Kassoc = 3.0 × 10^10^ M^−2^. These Job’s plot analyses were in agreement with the fluorescence titration analyses. Furthermore, ^1^H NMR titration of probe **1b** with Cu^2+^ in DMSO-d_6_ was undertaken to determine the complexation mode of **1-Cu**. As shown in Figure S5, some significant spectral changes are observed in the ^1^H NMR spectra on addition of Cu^2+^ ions. For the chromophore naphthalimide region, an amino hydrogen atom (NH) of the naphthalimide undergoes an upfield shift by 8.98–8.69 ppm, while the aromatic proton (H4) shift by 6.81–6.85 ppm. Similarly, in the presence of Cu^2+^, the aromatic protons of the triazole (H9 and H10) are downfield shifted by 0.05 ppm. These spectral changes suggest that the amino group of naphthalimide and the nitrogen atom of triazole moieties should be involved in the coordination with Cu^2+^ ions. Based on the titration profile, Job’s plot and structure-variation investigations, we proposed the possible mechanism and binding modes of probes **1a–1f** with Cu^2+^ (Figure S6). Probes **1b** and **1d** exhibited stronger recognition ability to Cu^2+^ than **1a** and **1c**. This phenomenon can be explained by steric effect. Probes **1b** (linked with 1,2-ethanediamine) and **1d** (linked with 1,6-hexylenediamine) gave smaller steric hindrance than **1a** (linked with dimethyl-1,2-ethanediamine) and **1c** (linked with piperazine), which made probes **1b** and**1d** have strong coordination affinity with Cu^2+^. Furthermore, the ability of Cu^2+^ recognition was also affected by the number of ligands. The probe **1d** grafted with two [12]aneN_3_ moieties exhibited stronger fluorescence quenching effect than **1e** modified with one [12]aneN_3_ moiety. Overall, the amino group linked with naphthalimide played an important role in quenching the fluorescence of the probes through a PET mechanism. The concerted interactions of the triazole-[12]aneN_3_ unit and the amino group with Cu^2+^ enhances the fluorescence quenching effect. These results may give us implies to design high performance fluorescence probes to recognition Cu^2+^.

**Figure 3.**
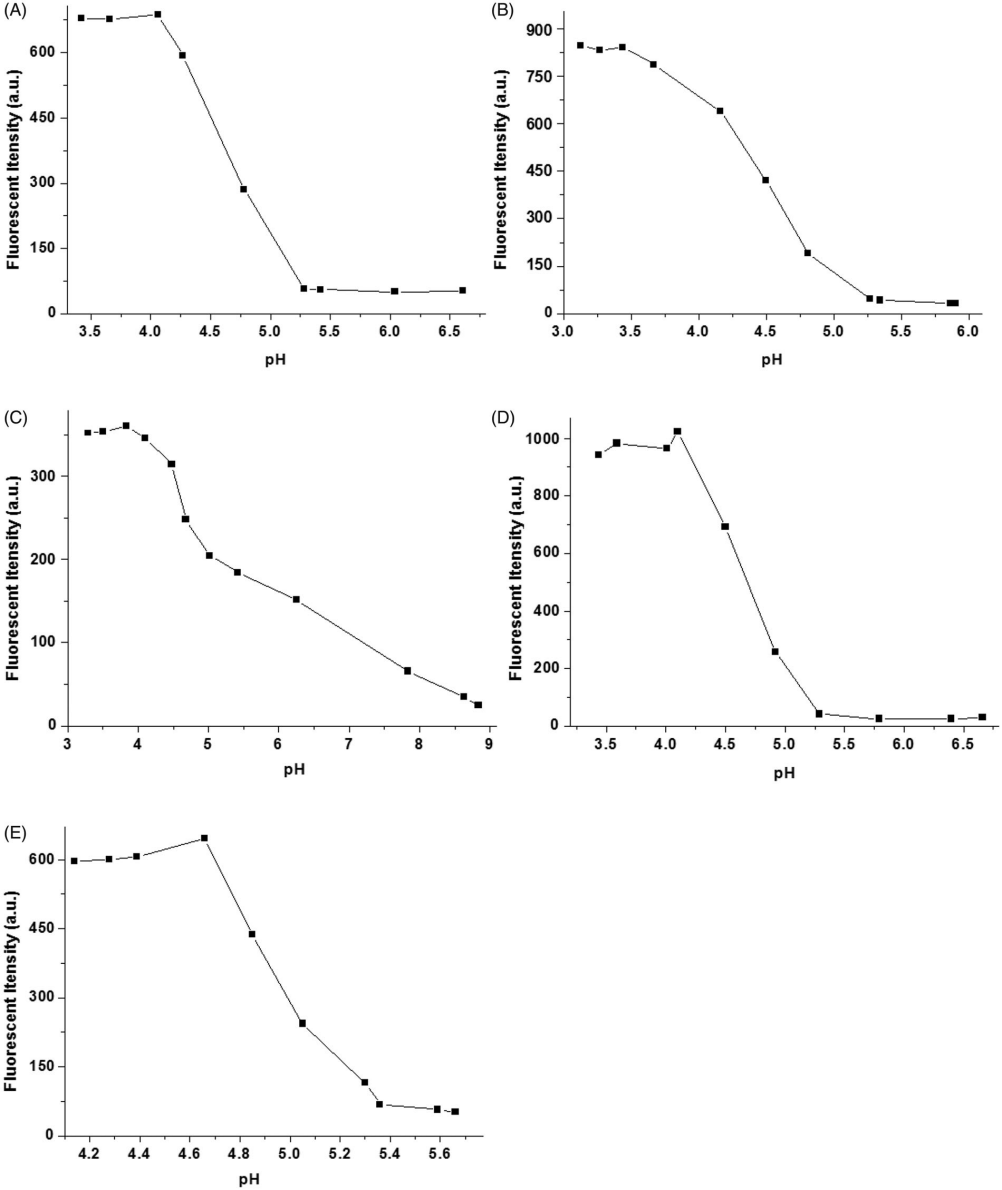
Fluorescence intensity of probes **1a**–**1e** (10 μM, A–E) with Cu^2+^ ions in water measured as a function of pH.

To investigate the effect of pH value on the fluorescence properties of complexes of **1-**Cu, we carried out fluorescence emission experiments of complexes of **1-**Cu at varying pH. As shown in Figure 3, pH value has a very strong effect on the complexes of **1-**Cu. Under acid pH (pH < 5) the fluorescence emission intensity was significantly enhanced with the increasing acidity of **1**-Cu complex solution. When the pH value reached 3.5, no obvious fluorescence change was observed. It is likely that the complete protonation of the nitrogen atoms of probes restricts the binding ability of probes **1a–1e** with Cu^2+^ under strongly acidic conditions. The binding ability of probes with Cu^2+^ is enhanced under neutral or basic condition, which leads to the fluorescence almost gets quenched and a very negligible change is observed (except **1c**) in the presence of Cu^2+^. Such results make us believe that **1a–1e** could become useful Cu^2+^ ion probes under biological conditions.

### 3.3. Cell imaging for Cu^2+^ recognition

**Figure 4.**
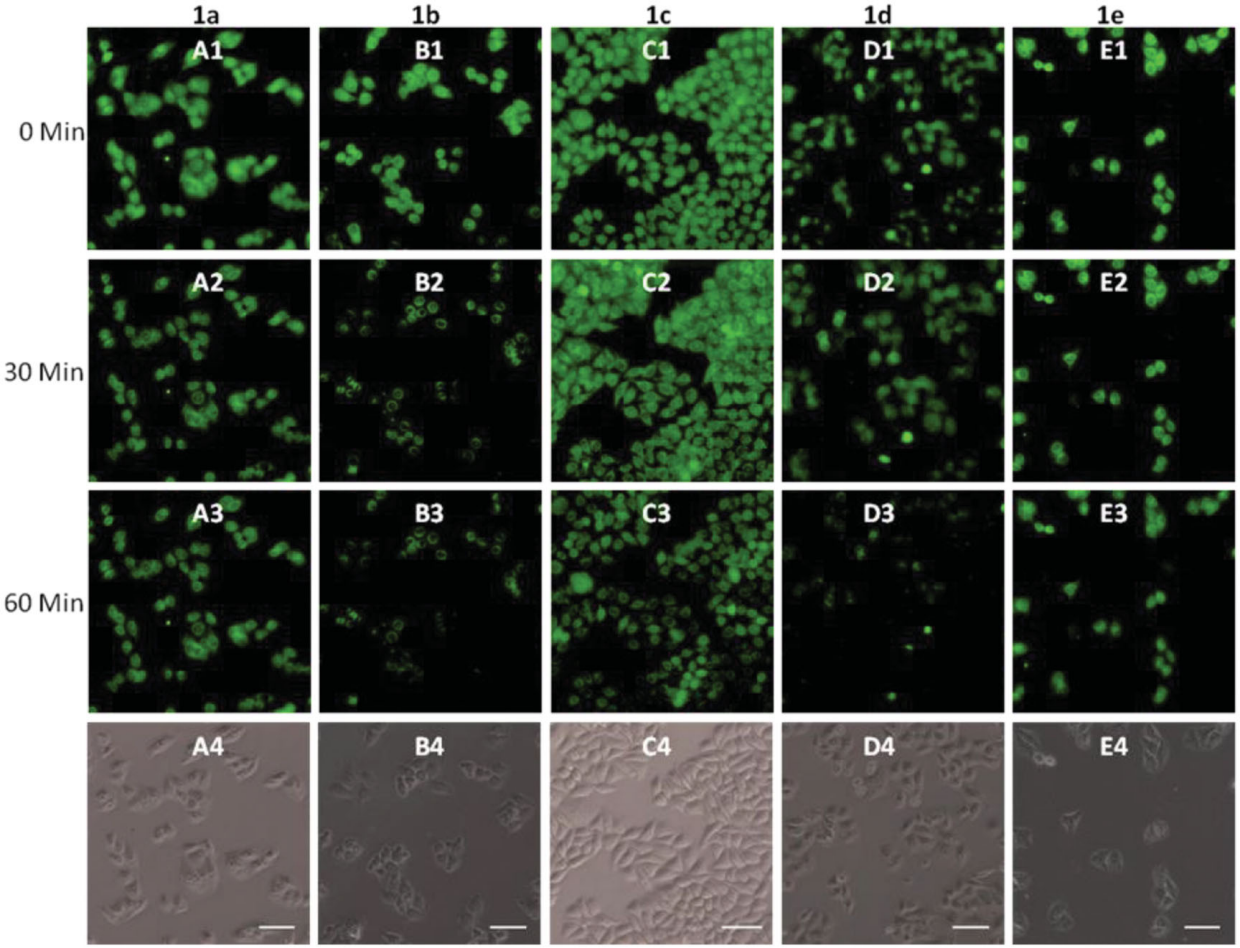
Fluorescence microscopy images of HeLa cells incubated with **1a**–**1e** (A1–E1) upon addition of Cu^2+^ for 30 minutes (A2–E2), 60 minutes (A3–E3). Bright field images of **1a**–**1e** (A4–E4). The scale bar in the figure is 30 μm.

**Figure 5.**
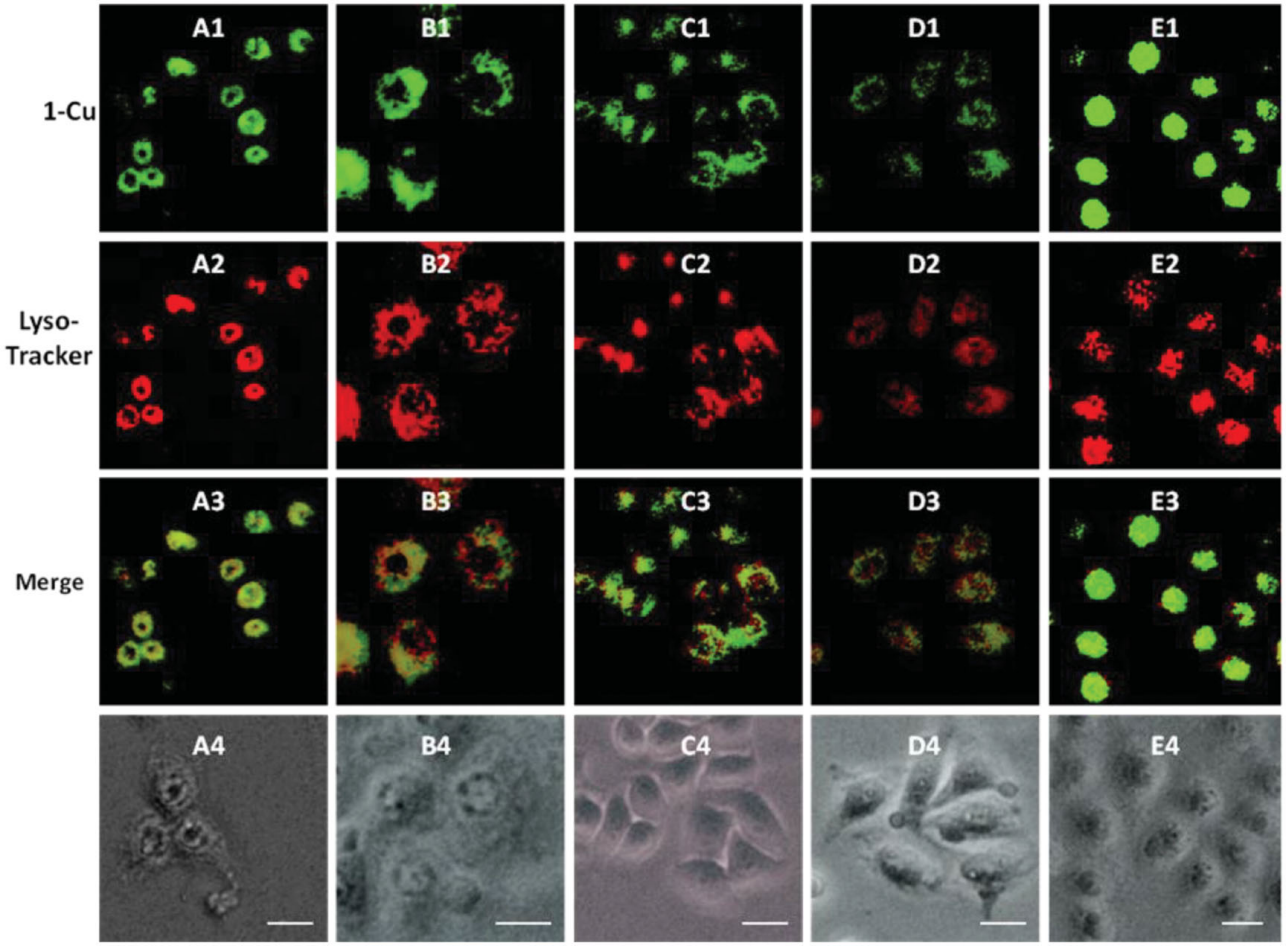
Fluorescence microscope images of HeLa cells incubated with complexes of **1a**–**1e** (20 μM) with Cu^2+^ (100 μM) for 0.5 h (A1–E1) and Lyso-Tracker Red for 0.5 h (A2–E2). Bright field images of **1a**–**1e** (A4–E4). The scale bar in the figure is 15 μm.

To further investigate the biological applications of **1a**–**1e**, the fluorescence microscopy experiment in HeLa cells was carried out. The cells were first treated with probes **1a**–**1e (**20 μM) for 30 min in DMEM medium at 37 °C, and then washed with PBS buffer, significant green fluorescence was observed under the fluorescence microscope (Figure 4(A1–E1)). The fluorescence intensity of **1a–1d** was decreased gradually after addition of Cu^2+^, and no obvious change was observed after 60 min (Figure 5(A3–E3)). However, the fluorescence was not completely quenched, which may be caused by the acidic environment of lysosomes. Strangely, the fluorescence intensity of **1e** was almost unaffected by the addition of Cu^2+^, which was not in accordance with the fluorometric titration spectra measured by fluorescence spectrophotometer (Figure 2(E)). This phenomenon should be ascribed to the special structure of **1e**, which was modified with only one [12]aneN_3_ unite, while other probes (**1a**, **1b**, **1c,** and **1d**) were modified with two [12]aneN_3_ unites. The less number of [12]aneN_3_ units made **1e** have weak coordination ability with Cu^2+^ as well as poor anti-interference performance especially in a complex intracellular environment, which led to the weak recognition for Cu^2+^ in the cells.

### 3.4. Lysosome imaging

It is well known that lysosome is one of important organelles in eukaryotic cells. It plays a key role in the degradation of macromolecules and cell components. The pH value in lysosomes ranges from 4.0 to 6.0, while the cytoplasm is about 7.2. The 1-Cu complexes exhibit strong fluorescence emission under acid conditions, while almost no fluorescence emission under neutral conditions, which make us believe that 1-Cu complexes can be used for lysosome imaging. In order to confirm above idea, lysosome imaging experiment was carried out in HeLa cells. Lyso-Tracker Red, a commercially available lysosome specific staining probe, was used to co-stain HeLa cells with **1**-Cu complexes. HeLa cells were firstly incubated with **1**-Cu complexes for 30 min, and then the medium was replaced with fresh medium containing Lyso-Tracker Red and incubated for another 30 min. As shown in Figure 5, the green emission of **1a**-Cu, **1b**-Cu, **1c**-Cu, and **1d**-Cu (Figure 5(A1–D1)) and the red emission of Lyso-Tracker Red (Figure 5(A2–D2)) are nearly overlapping (Figure 5(A3–D3)). The correlation coefficient of overlap between green and red channel has been analyzed by IPP software. The Overlap coefficients of **1a**–**1e** with Lyso-Tracker Red are 0.93, 0.85, 0.88, 0.95 and 0.81, respectively. These results indicate that the 1-Cu complexes can be applied to fluorescence probes lysosome imaging.

### 3.5. Characterization of 1a–1e/RNA (DNA) complexes

**Figure 6.**
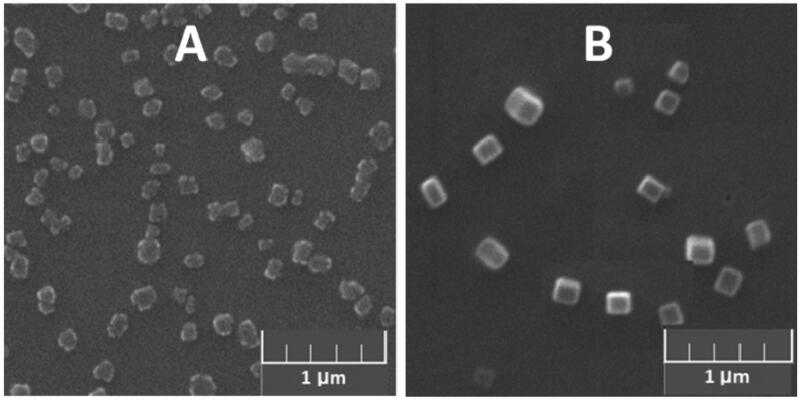
SEM image of (A) **1b**/RNA complexes and (B) **1b**/DNA complexes at a concentration of 20 μM (DNA or RNA: 9 μg/mL).

**1a**–**1e**/DNA (RNA) complexes were prepared by combining constant amount of DNA or RNA with varying concentrations of MFCs. The changes of particle sizes and zeta-potentials were measured at different concentrations of MFCs **1a**–**1e** ranging from 10 μM to 60 μM *via* dynamic light scattering (DLS). As shown in Figures S7(A1–E1), these materials can condense DNA/RNA into nanoparticles ranging from 50 nm to 500 nm, and the complexes of **1**/RNA particles were slight smaller than **1**/DNA particles. SEM was also used to directly visualize the morphology and size of **1b**/RNA (DNA) complexes. As shown in Figure 6, **1****b**/DNA complexes have homogeneous oblong shape with diameter of about 150 nm, while **1b**/RNA complexes have an irregular shape, and it looks something like a cauliflower, the size of which was little smaller than particles formed by **1b**/DNA complexes. The zeta potentials of the complexes of **1/**RNA (DNA) were also measured *via* DLS (Figure S6(A2–E2)). The zeta potentials rose along with the increasing of concentration of **1a**–**1e**. Compared to other materials, the complexes of **1e** with DNA or RNA showed larger zeta potentials. At the concentration of 20 μM, the surface charges of complexes derived from **1e** turned to positive, while other complexes still remained negatively charged. DLS and SEM results suggest that these multifunctional compounds have the potential to be used as nonviral vectors to deliver DNA or RNA.

### 3.6. Cell uptake of 1a–1e/RNA (DNA) complexes

To examine the possibility of using MFCs **1a**–**1e** as non-viral vectors for gene delivery, cellular uptake experiment was carried out by using Cy5-labeled siRNA and dsDNA, 25 kDa PEI and lipofectamine 2000 were served as positive controls. The Cy5-labeled RNA/DNA emitted red light, while MFCs **1a**–**1e** gave rise to green fluorescence, which enabled us to observe MFCs **1a**–**1e** and RNA/DNA simultaneously under fluorescence microscopy.

**Figure 7.**
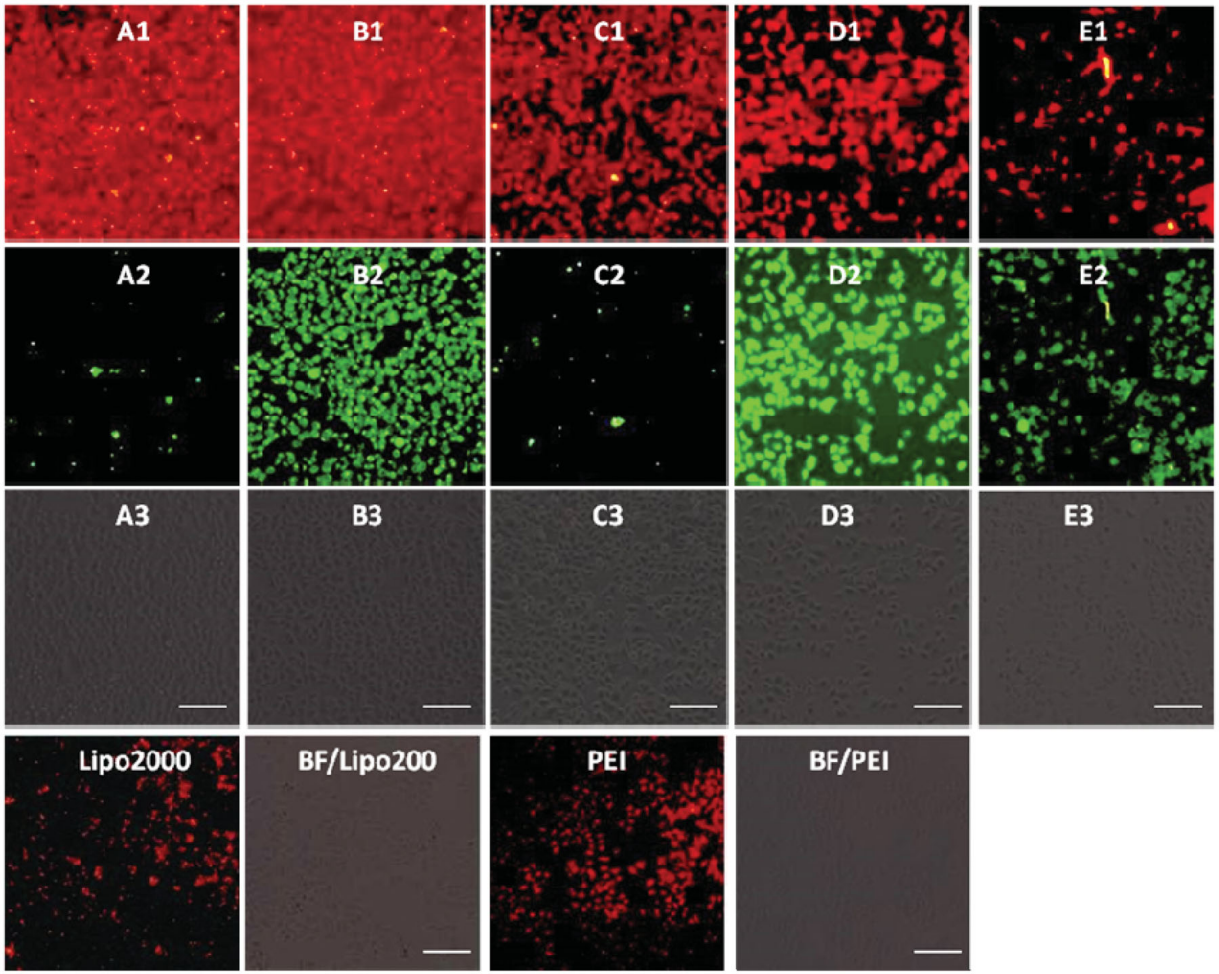
Fluorescence microscopy images of HeLa cells transfected with Cy5- labeled RNA (9 μg/mL) by MFCs **1a**–**1e** at the concentration of 20 μM, 20 kD PEI and lipofectamine 2000 as positive control. (A1–E1) red channels, (A2–E2) green channels, (A3–E3) bright field images. The scale bar in the figure is 100 μm.

Before a direct comparison on the cellular uptake capacities, the optimal concentration (**1d**) and w/w ratio (PEI/RNA and lipofectamine2000/RNA) were screened on the HeLa cells. As shown in Figures S7–S9, the MTC **1d** exhibited the best RNA delivery ability at the concentration of 20 μM, while the optimized w/w ratios for commercial agents PEI and lipofectamine 2000 were 5/1 and 20/1, respectively. The cellular uptake experiments were then conducted using HeLa cells at the optimal gene delivery condition of each material. As shown in Figure 7, these materials modified with shorter linker (such as compound **1a** and **1b**) showed stronger red fluorescence than those with longer linker, and almost all the cells were stained with Cy5-labeled RNA, indicating their excellent RNA delivery ability. The commercial agents 25 kDa PEI and lipofectamine 2000, by contrast, gave much weaker red fluorescence emission than **1a** and **1b** in HeLa cells. We also found that red fluorescence and green fluorescence did not overlap well. It can be explained from two aspects: on the one hand, the fluorescence intensity of compounds **1a** and **1e** was much weaker than **1b**, **1c,** and **1e**, which can be observed in Figure 1. Therefore, much green fluorescence (**1a** and **1c**) was loose when we take pictures at same conditions (**1b**, **1d,** and **1e** can show suitable fluorescence) by under fluorescence microscopy. On the other hand, all the images were obtained after 4 h cell uptake, some RNA may be released from the complexes. Therefore, the images from red channel are not overlapped with the green channel very well. The RNA delivery ability of **1a**–**1e** was further evaluated in HepG2, U2Os and MC3T3-E1 cell lines at the concentration of 20 μM. As shown in Figures S10–S12, the intensity of red fluorescence observed in above three cell lines was far weaker than that observed in HeLa cells, indicating that these five compounds, especially for **1a** and **1b**, have excellent HeLa cell selectivity in RNA delivery. While for lipofectamine 2000 and PEI, they showed almost no cell-selectivity.

**Figure 8.**
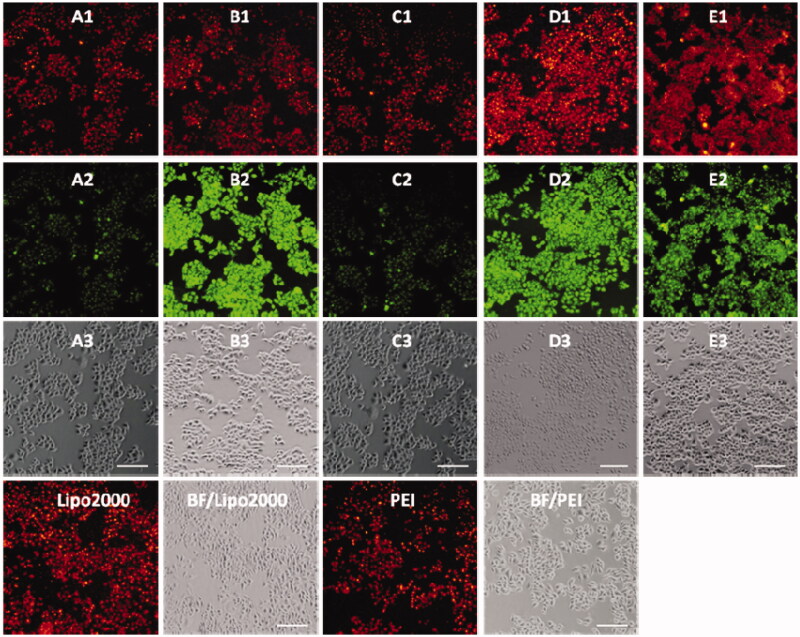
Fluorescence microscopy images of HeLa cells transfected with Cy5-labeled DNA (9 μg/mL) by MFCs **1a**–**1e** at the concentration of 20 μM, 20 kD PEI and lipofectamiine 2000 as positive control. (A1–E1) red channels, (A2–E2) green channels, (A3–E3) bright field images. The scale bar in the figure is 100 μm.

**Figure 9.**
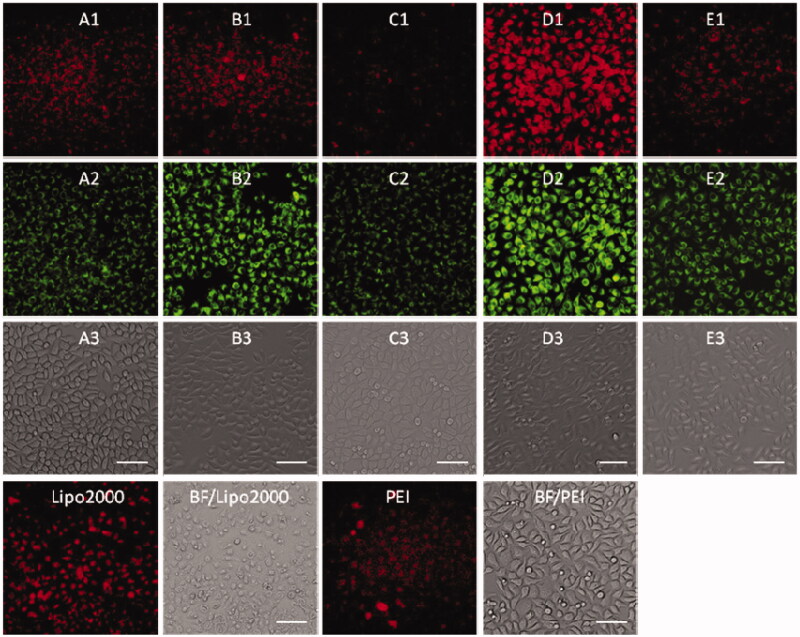
Fluorescence microscope images of HepG2 cells transfected with Cy5-labeled DNA (9 μg/mL) by MFCs **1a**–**1e** at the concentration of 20 μM, 25 kD PEI and lipofectamiine 2000 as positive control. (A1–E1) red channels, (A2–E2) green channels, (A3–E3) bright field images. The scale bar in the figure is 50 μm.

Subsequently, DNA delivery ability of **1a**–**1e** was investigated at the concentration of 20 μM in HeLa and HepG2 cell lines. As shown in Figures 8 and 9, unlike RNA delivery, longer hydrophobic chain modified MTC **1d** gave higher DNA delivery efficiency than shorter chain modified compounds, which was slightly higher than commercial materials lipofectamine 2000 and PEI in HeLa and HepG2 cells. These results indicated that DNA or RNA delivery ability is closely related with the structure of multifunctional compounds. The short chain linked compound **1b** has excellent RNA delivery ability, while long chain modified compound **1d** exhibits high DNA delivery efficiency, especially in HeLa cells, their performance is superior to lipofectamine 2000 and PEI.

### 3.7. Cytotoxicity

**Figure 10.**
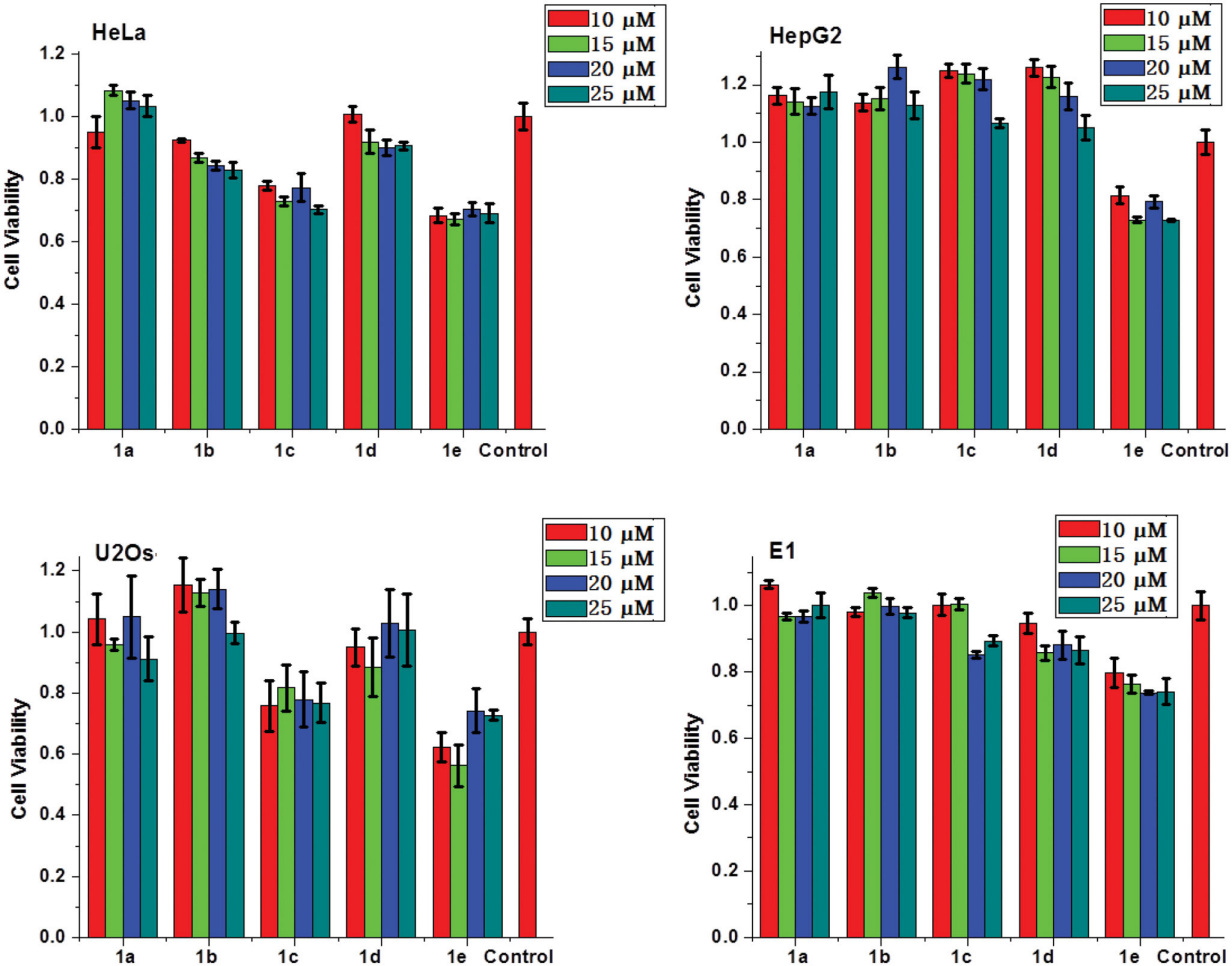
Cytotoxicities of the complexes of MFCs **1a**–**1e**/RNA at different concetrations on HeLa, HepG2, U2Os and MC3T3-E1 cells.

Besides high DNA and RNA delivery efficiency, the biocompatibility of gene vector is also very important in clinical applications. Therefore, MTT assay was performed to evaluate the cytotoxicity of the complexes of **1a–1e**/RNA and **1a**–**1e**/DNA in HeLa, HepG2, U2Os, and MC3T3-E1, cells. It should be noted that the concentration of **1a**–**1e** was ranged from 10 μM to 25 μM, which was consistent with cellular uptake experiment. As shown in Figures 10 and S13, these multifunctional compounds (except **1e)** have low cytotoxicity, especially for complexes of **1d** with RNA or DNA, nearly no cytotoxicity was observed on HepG2 and U2Os cells. These results suggest that these materials can achieve high delivery efficacy and good biocompatibility when used as non-viral gene vectors.

## 4. Conclusion

A series of [12]aneN_3_-based naphthalimide MFCs **1a**–**1e** were designed and synthesized. various linkers and different numbers of [12]aneN_3_ units were employed to facilitate the investigation of the structure-performance relationships. Some of these small organic compounds exhibited excellent performance on detection of Cu^2+^, lysosomal staining as well as RNA and DNA delivery. It was found that the probes with long hydrophobic chains and low steric hindrance linkers showed higher recognition ability toward Cu^2+^ (**1d **>** 1b **>** 1a **>** 1c**). The sensing mechanisms and binding modes of MFCs with Cu^2+^ were confirmed by fluorescence titration, Job’s plot analyses, and ^1^H-NMR titration. Besides, their complexes with Cu^2+^ (**1**-Cu) could also selectively stain lysosome in HeLa cells due to the acidic environment of lysosome. Cellular uptake experiments revealed that MFCs **1a** and **1b** have excellent HeLa cell selectivity in RNA delivery, and their performance was far better than lipofectamine 2000 and 25 kDa PEI. While for DNA delivery, long chain modified MFC **1d** exhibited higher delivery efficiency than other compounds. These results will give us further insight to design high performance multifunctional compounds for fluorescence probes and non-viral vectors.

## Supplementary Material

Supplemental Material
