# Supplementary material for: [12]aneN3-based multifunctional compounds as fluorescent probes and nucleic acids delivering agents
Source: Drug Deliv. 2019 Dec 20;27(1):66–80. doi: 10.1080/10717544.2019.1704943 (PMC6968532; doi:10.1080/10717544.2019.1704943)
Supplement: Supplemental Material [file IDRD_A_1704943_SM0920.pdf]

Electronic Supplementary Information (ESI)

[12]aneN_3-based multifunctional compounds as fluorescent probes and nucleic acids delivering agents

Yong-Guang Gao,[ab*] Shuyuan Huangfu,[a] Suryaji Patil,[a] Quan Tang,[b] Wan Sun,[b] Yu Li,[a] Zhong-Lin Lu[b*] and Ai-Rong Qian[a*]

[a] Lab for Bone Metabolism, Key Lab for Space Biosciences and Biotechnology, School of Life Sciences, Northwestern Polytechnical University, Xi'an, Shaanxi 710072, China

[b.] *KeyLaboratory of Theoretical and Computational Photochemistry, Ministry of Education, College of Chemistry, Beijing Normal University, Xinjiekouwai Street 19, Beijing 100875， China.*

** Corresponding authors, Tel.: +86-29-88460660; gaoyongguang@nwpu.edu.cn (Y.-G. Gao). Tel.: +86 10 58801804. luzl@bnu.edu.cn (Z.-L. Lu); Tel.: +86-29-88491840, qianair@nwpu.edu.cn (A.-R. Qian).*

Contents

1. Spectroscopic properties of 1a-1e

2. Characterization of 1a-1e/RNA (DNA) complexes

3. Cell uptake of 1a-1e/RNA (DNA) complexes

4. Cytotoxicity

5. Spectroscopic data of compounds

6. Spectra

1. Spectroscopic properties of 1a-1e

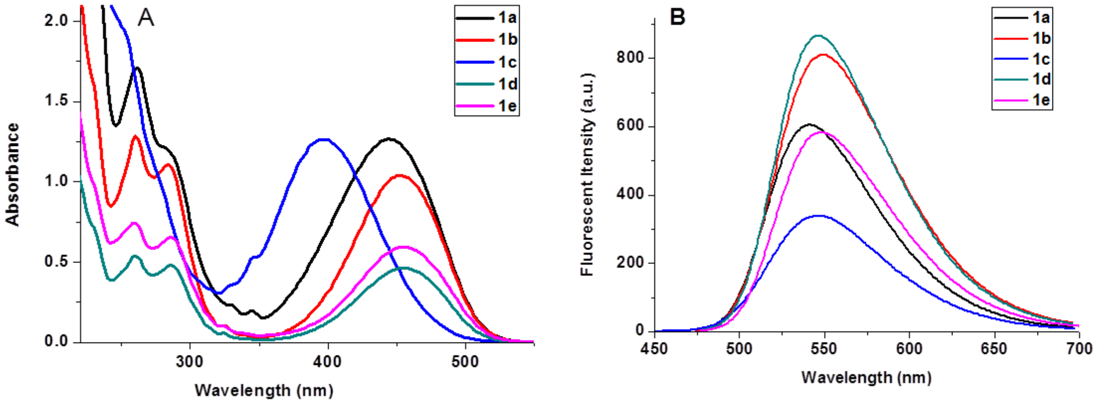

Fig. S1 The absorption spectra (50 μM) and fluorescence (10 μM) spectra of **1a-1e** in water-Tris-HCl buffer (1 mM, pH = 7.2).

Table S1 The fluorescent intensity changes of **1a-1e** after addition of Cu^{2+} ions

Probes	Fluorescent Intensity (F0, before addition of Cu^{2+})	Fluorescent Intensity (F, after addition of Cu^{2+})	Fluorescent Changes (F0/ F)
1a	605.86	60.91	9.95
1b	810.36	17.15	47.25
1c	339.10	57.22	5.93
1d	866.29	13.60	63.69
1e	582.98	39.97	14.59

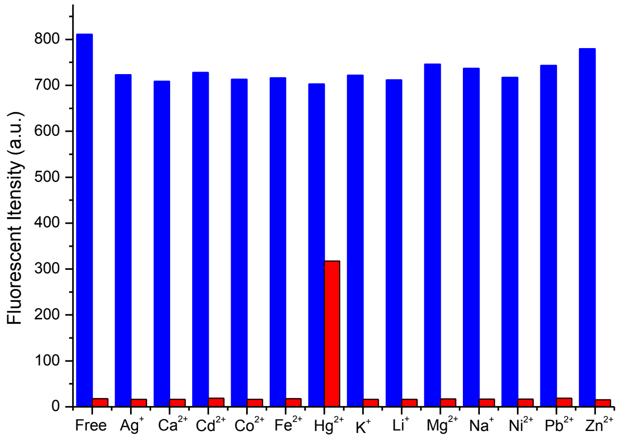

Fig. S2 Selectivity studies of **1b** with Cu^{2+} in the presence of other metal ions. Blue bars represent the addition of the competing metal ion (100 μM) to the solution of the 1 (10 μM). Red bars represent the addition of Cu^{2+} (30 μM) to the solution containing other metal ions.

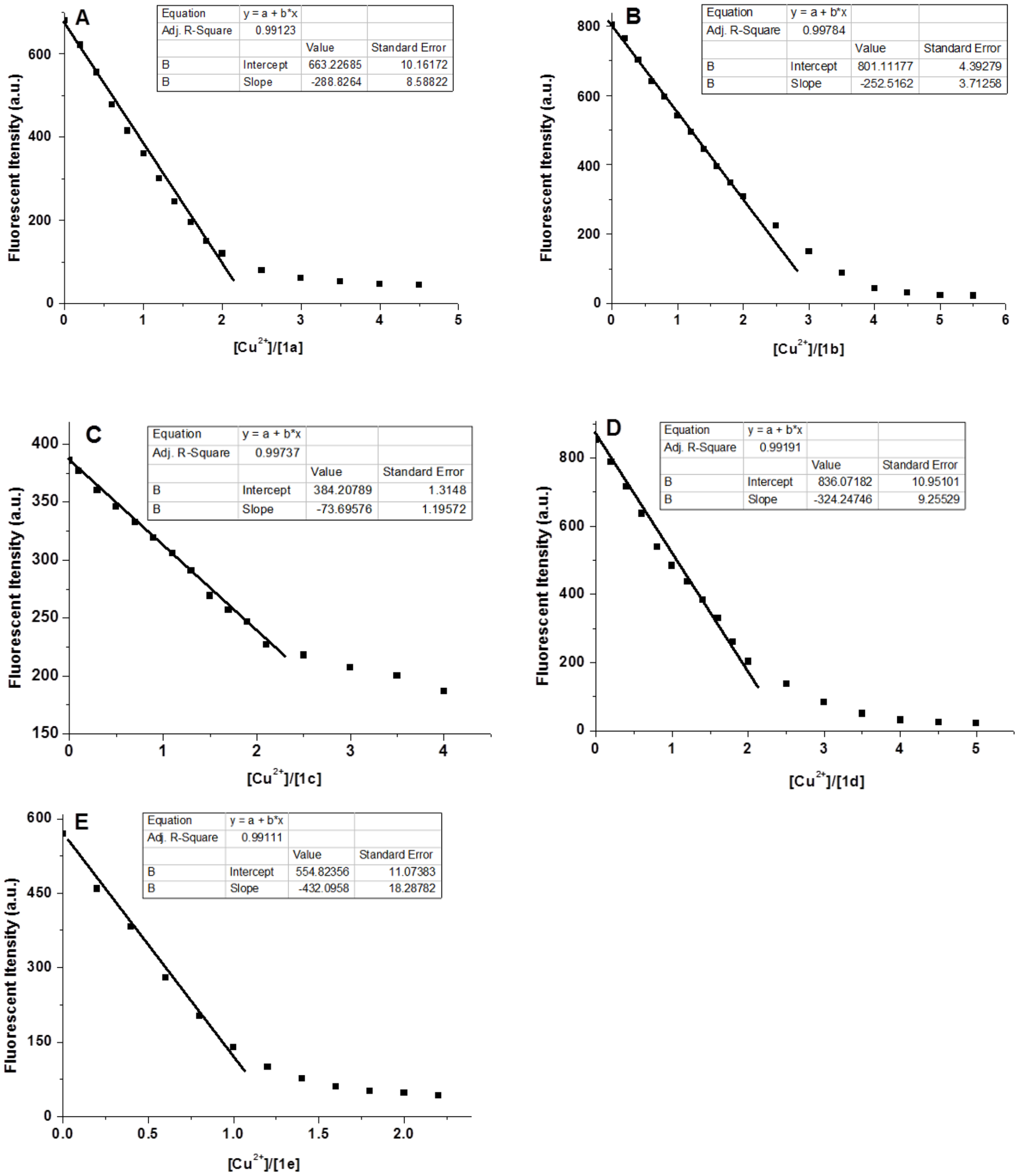

Fig. S3 Plots of fluorescence intensity of **1a-1e** as a function of [Cu^{2+}]/[**1**]. The standard deviations obtained by fluorescence responses of **1a-1e** were determined to be σ = 1.17, 0.63, 1.08, 0.13 and 0.34 for Cu^{2+}, therefore, the detection limits were calculated by the formula (3σ/k) and given the results of 1.21×10^{-8} M, 7.48×10^{-9} M, 4.40×10^{-8} M, 1.23×10^{-9} M and 2.36×10^{-9} M, respectively.

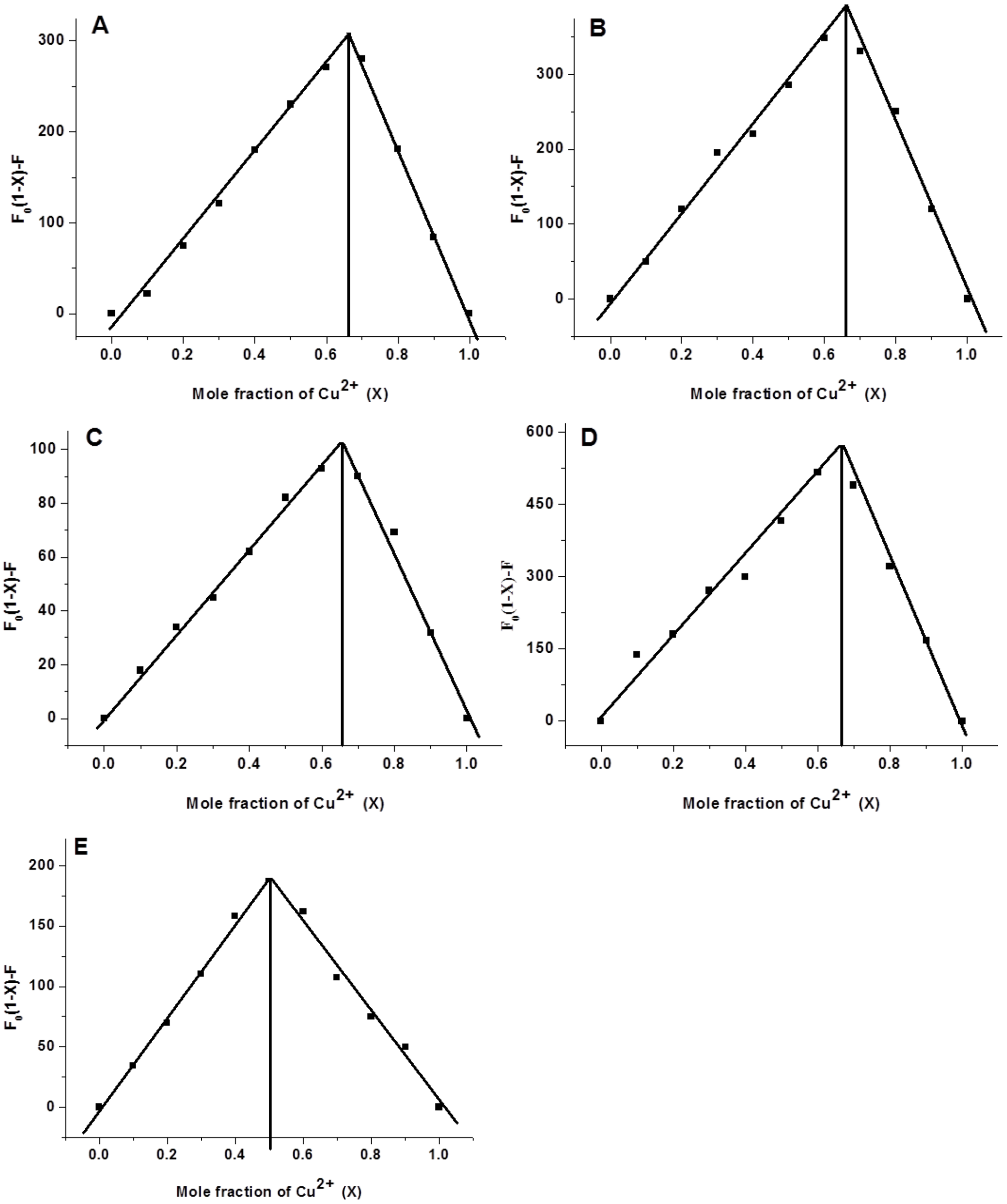

Fig. S4 Job's plot showing the 1:1 (**1a**-**1d**/Cu^{2+}) and 1:2 (**1e**/Cu^{2+}) complex stiochiometry, mole fraction of Cu^{2+} X = $[Cu^{2+}]/([Cu^{2+}]+[\mathbf{1}])$, $[Cu^{2+}]+[\mathbf{1}]$ = 30 µM in Tris-HCl buffer.

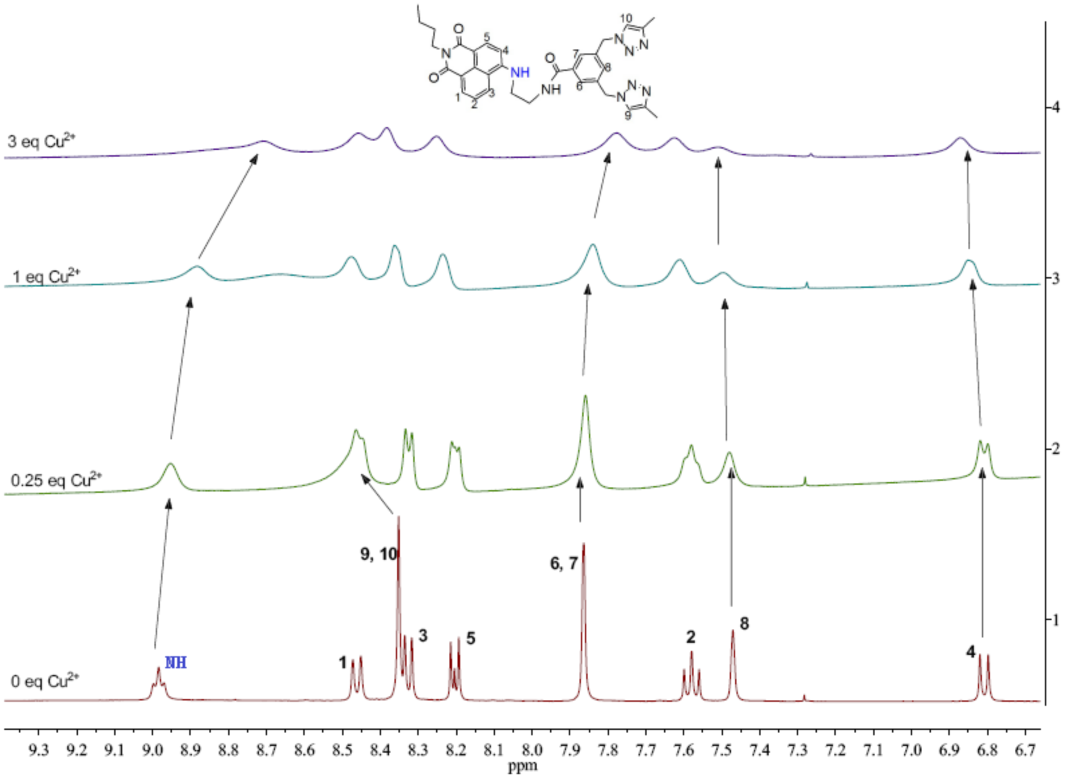

Fig.S5 ^{1}H NMR of compound **1b** upon titration of $Cu(ClO_4)_2$ (CD_3SOCD_3)

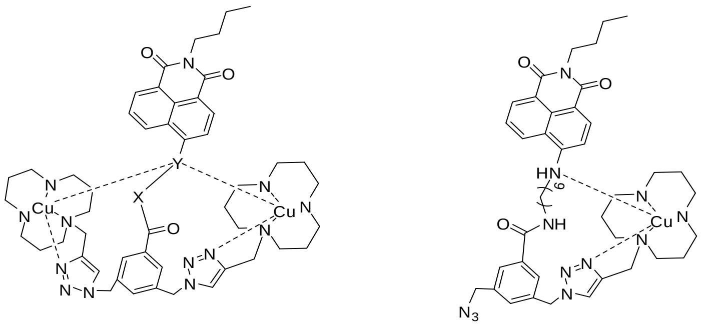

(**1a-1d**)-Cu　　**1e**-Cu

Fig.S6 The proposed binding modes of **1** and Cu^{2+}

2. Characterization of 1a-1e/RNA (DNA) complexes

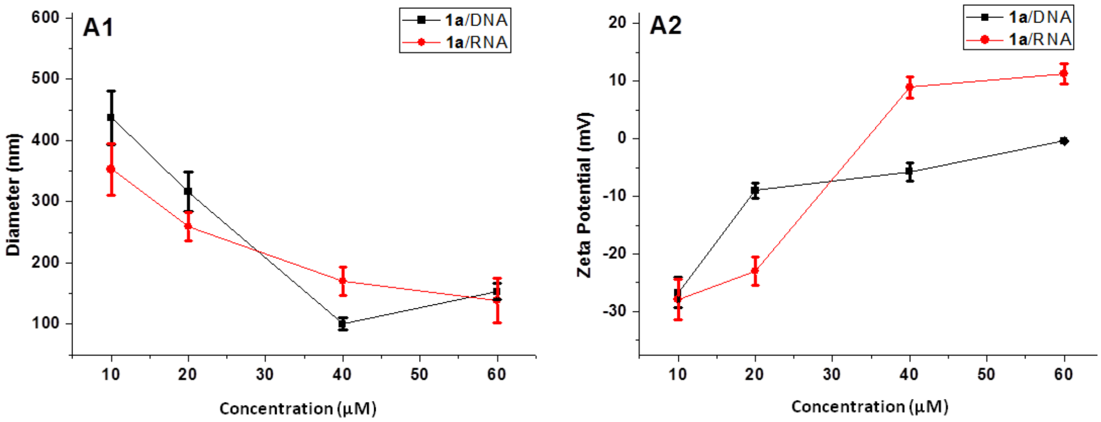

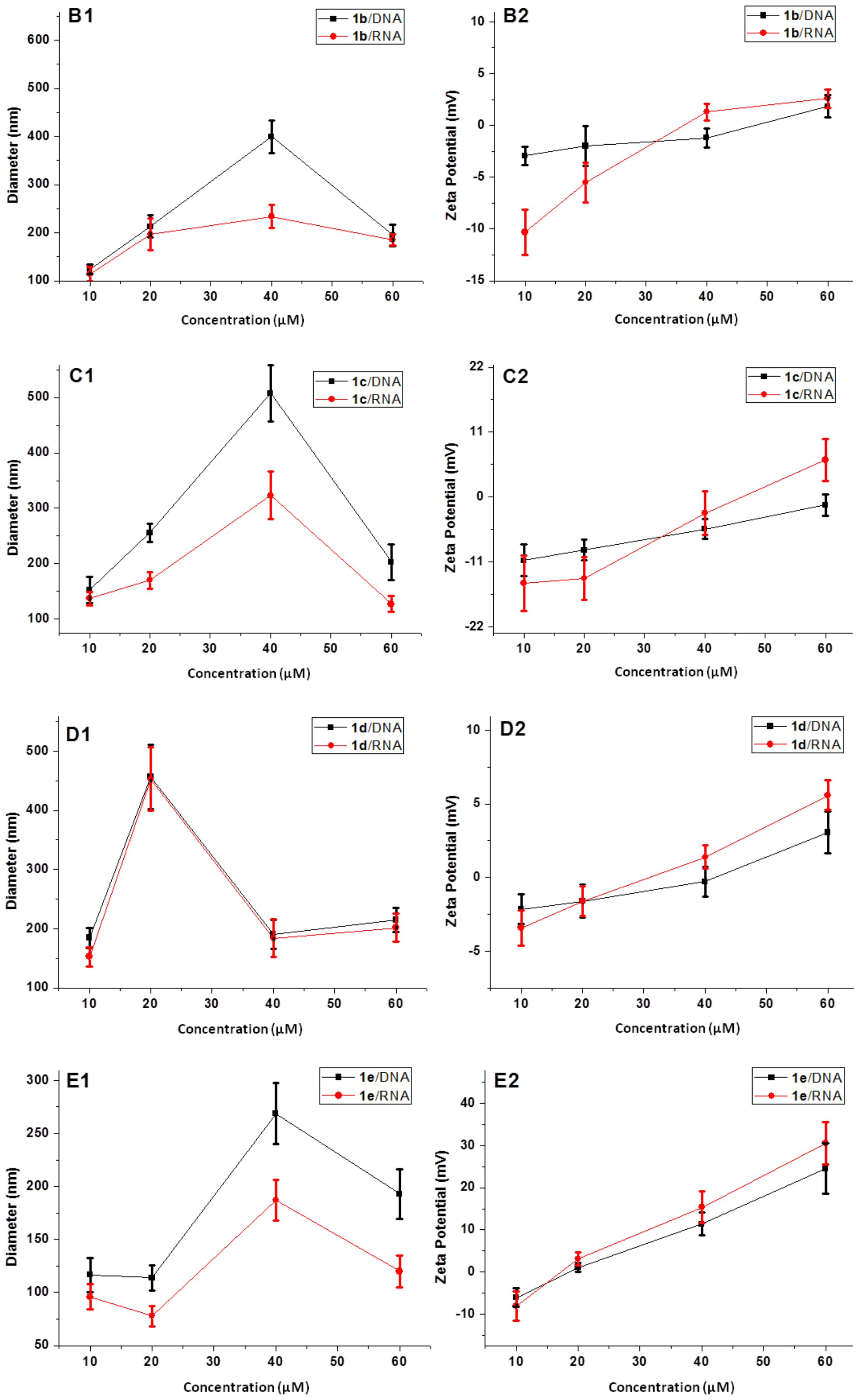
B1
1b/DNA
1b/RNA
Diameter (nm)
Concentration (µM)
B2
1b/DNA
1b/RNA
Zeta Potential (mV)
Concentration (µM)
C1
1c/DNA
1c/RNA
Diameter (nm)
Concentration (µM)
C2
1c/DNA
1c/RNA
Zeta Potential (mV)
Concentration (µM)
D1
1d/DNA
1d/RNA
Diameter (nm)
Concentration (µM)
D2
1d/DNA
1d/RNA
Zeta Potential (mV)
Concentration (µM)
E1
1e/DNA
1e/RNA
Diameter (nm)
Concentration (µM)
E2
1e/DNA
1e/RNA
Zeta Potential (mV)
Concentration (µM)

Fig. S7 (A1-E1) Mean diameter and (A2-E2) zeta potential of **1a**-**1e**/DNA complexes and **1a**-**1e**/RNA complexes obtained at various concentrations by DLS.

3 Cell uptake of 1a-1e/RNA (DNA) complexes

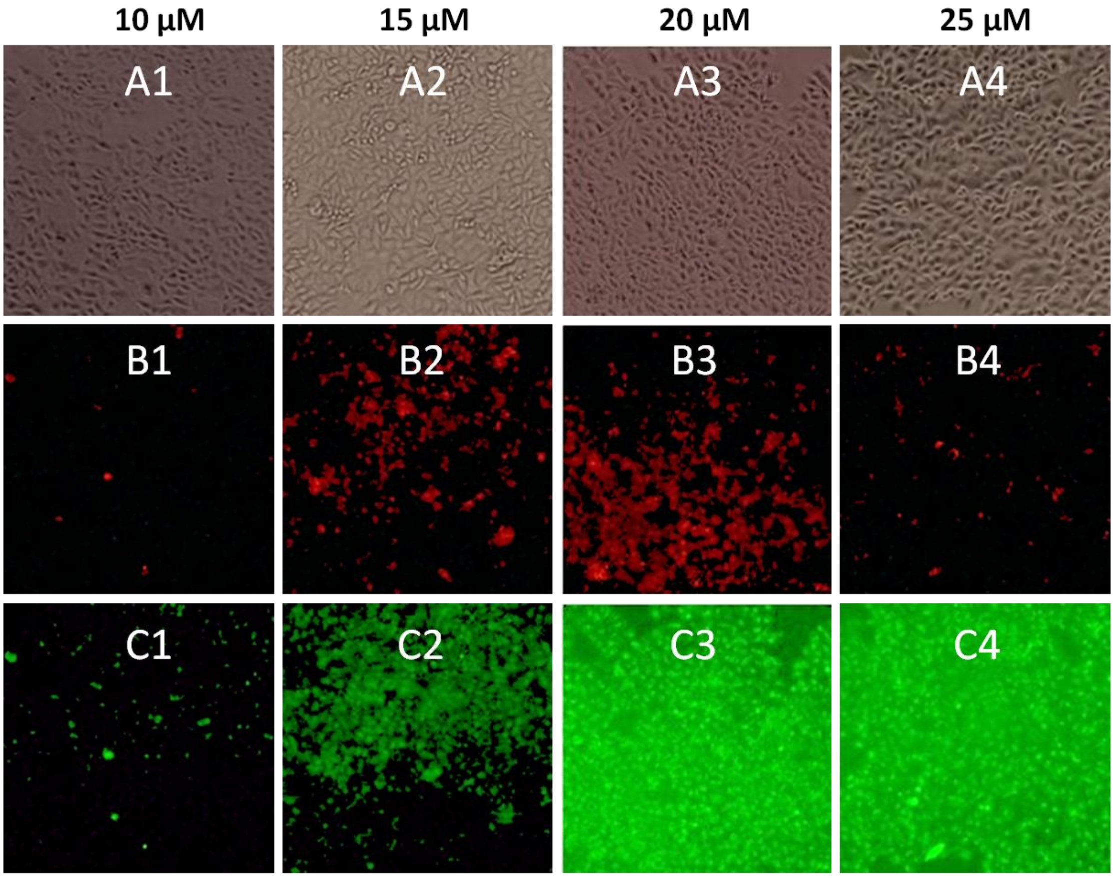

Fig. S7 Fluorescence microscope images of HeLa cells transfected with Cy5-labaled siRNA (9 μg/mL) by **1b** at different concentrations. A1-A4: BF, B1-B4: red channels, C1-C4: green channels.

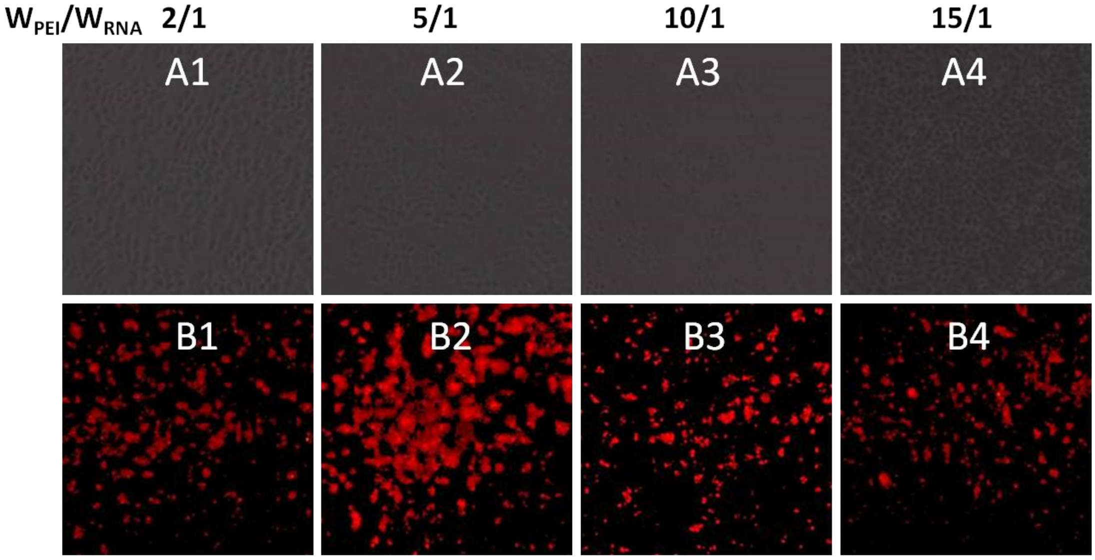

Fig. S8 Fluorescence microscope images of HeLa cells transfected with Cy5-labaled siRNA (9 μg/mL) by 25 KDa PEI at different weight ratios. A1-A4: BF, B1-B4: red channels**.**

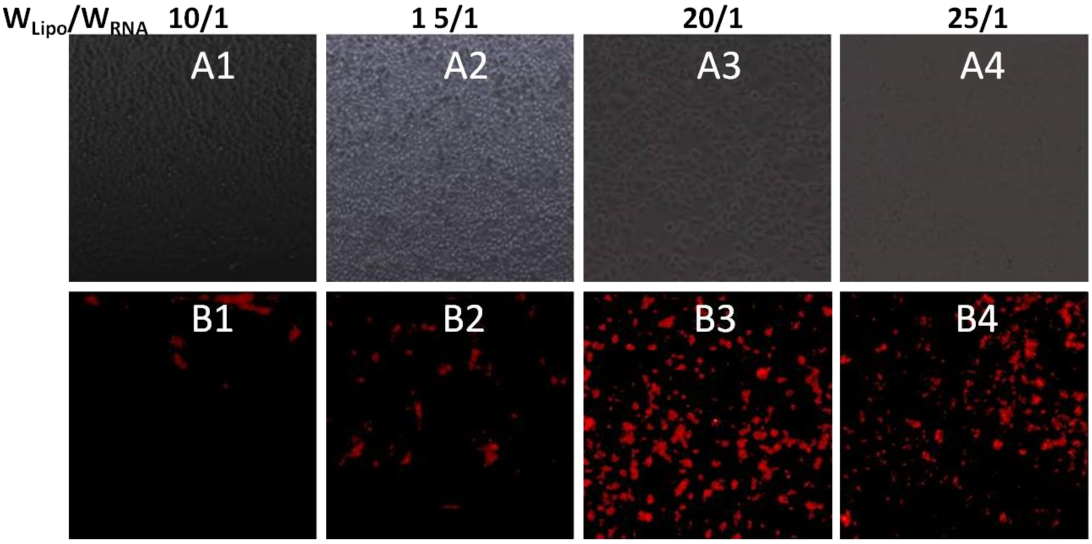

Fig. S9 Fluorescence microscope images of HeLa cells transfected with Cy5-labaled siRNA (9 µg/mL) by lipofectamine 2000 at different weight ratios. A1-A4: BF, B1-B4: red channels.

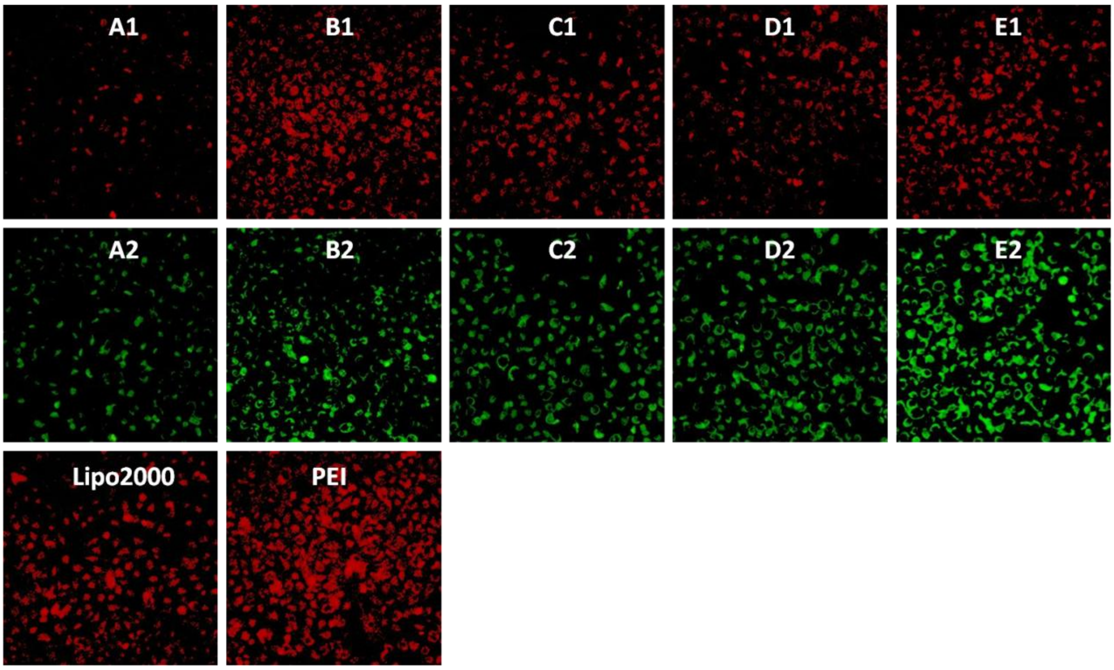

Fig. S10 Fluorescence microscope images of HepG2 cells transfected with Cy5-labaled siRNA (9 µg/mL) by MFCs **1a**-**1e** at the concentration of 20 µM, 25 kD PEI and lipofectamiine 2000 as positive control. A1-E1: red channels, A2-E2: green channels.

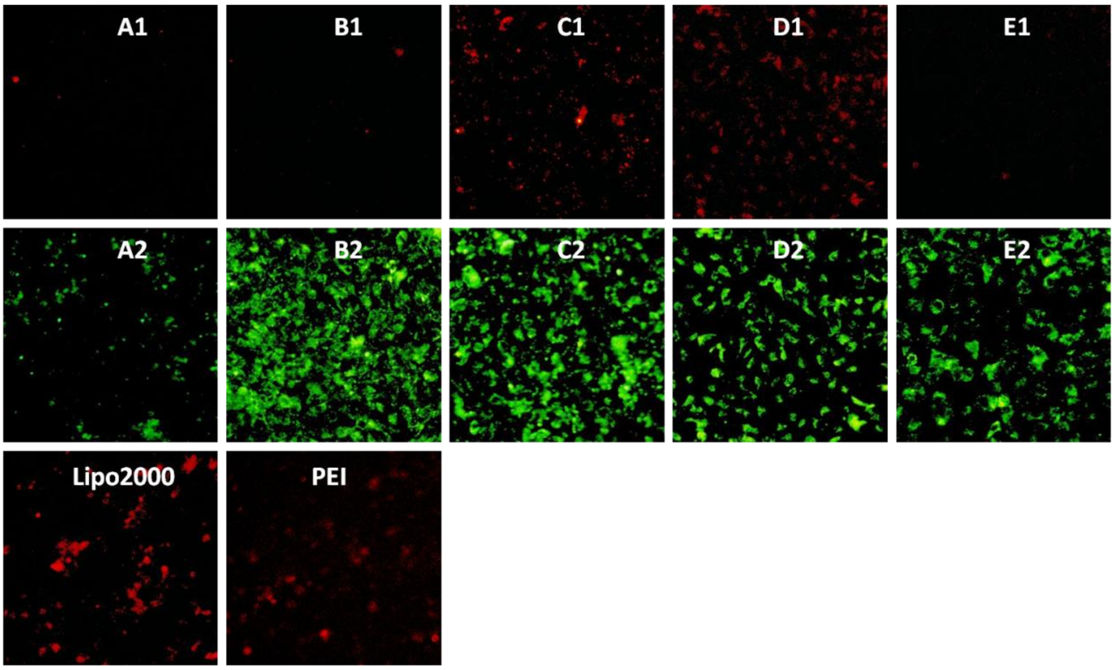

Fig. S11 Fluorescence microscope images of U2Os cells transfected with Cy5-labaled siRNA (9 μg/mL) by MFCs **1a**-**1e** at the concentration of 20 μM, 25 kD PEI and lipofectamiine 2000 as positive control. A1-E1: red channels, A2-E2: green channels.

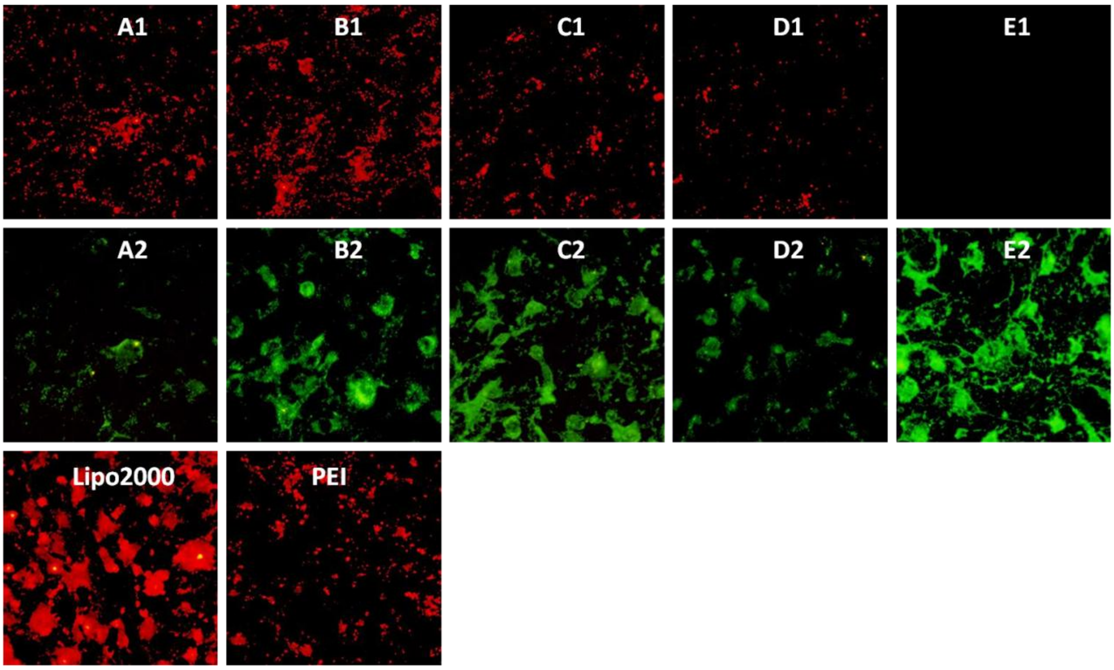

Fig. S12 Fluorescence microscope images of MC3T3-E1 cells transfected with Cy5-labaled siRNA (9 μg/mL) by MFCs 1a-1e at the concentration of 20 μM, 25 kD PEI and lipofectamiine 2000 as positive control. A1-E1: red channels, A2-E2: green channels.

4. Cytotoxicity

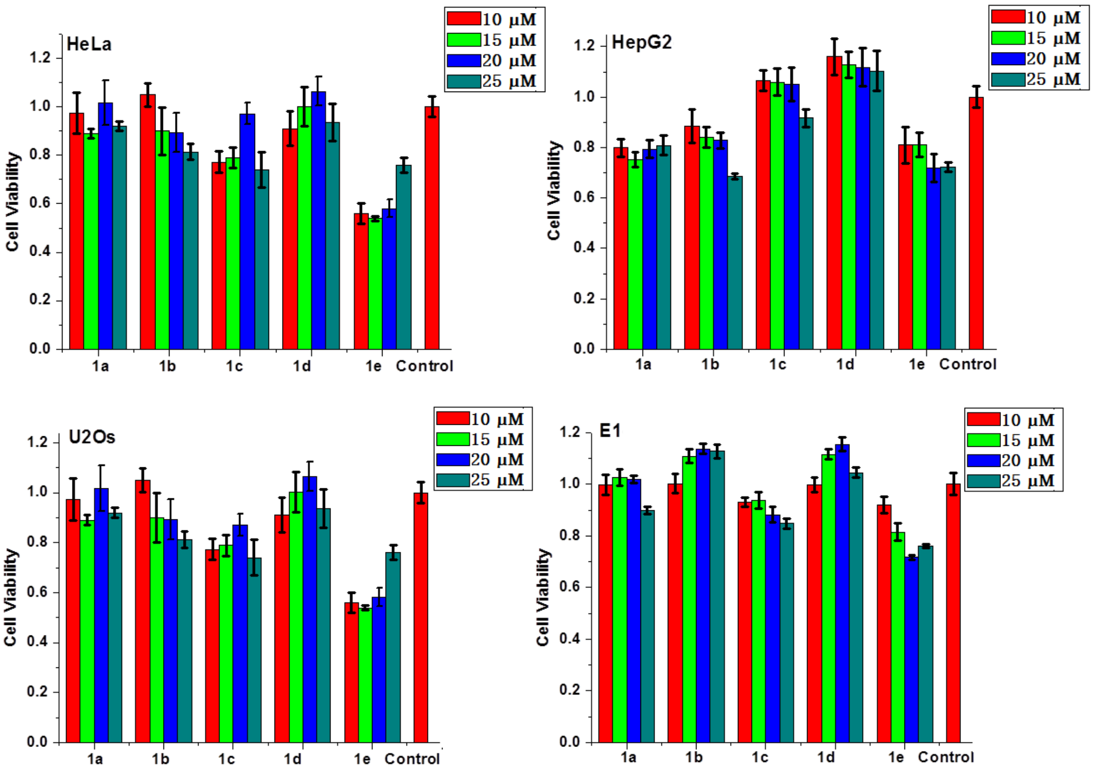

Fig. S13 Cytotoxicities of the complexes of MFCs **1a**-**1e**/DNA at different concentrations on HeLa, HepG2, U2Os and MC3T3-E1 cells.

5. Spectroscopic data of compounds

4a: 59%; ^{1}H NMR (400 MHz, $CDCl_3$) δ 8.59 (d, *J* = 7.2 Hz, 1H), 8.48 (dd, *J* = 17.1, 8.1 Hz, 2H), 7.70 (t, *J* = 7.9 Hz, 1H), 7.38 (s, 2H), 7.28 - 7.09 (m, 4H), 5.45 (s, 4H), 4.15 (t, *J* = 7.2 Hz, 2H), 3.83 - 3.76 (m, 8H), 3.33 (s, 16H), 3.19 (s, 3H), 2.65 (s, 3H), 2.42 (s, 8H), 1.88 - 1.82 (m, 12H), 1.73 - 1.65 (m, 2H), 1.45 (s, 38H), 0.96 (t, *J* = 7.2 Hz, 3H); ^{13}C NMR (101 MHz, $CDCl_3$) δ 169.68, 164.15, 163.76, 155.96, 143.89, 137.52, 136.45, 132.15, 130.98, 130.44, 129.94, 127.91, 126.38, 125.16, 122.93, 115.55, 114.88, 79.07, 53.01, 49.56, 46.52, 45.30, 43.70, 41.90, 39.85, 37.62, 30.11, 28.36, 27.62, 25.96, 20.22, 13.73; IR (KBr, cm^{-1}): 3338.86, 3127.11, 2967.17, 2931.93, 1690.36, 1649.70, 1581.93 1416.57, 1359.64, 1245.78, 1169.88, 776.81; EI-MS calcd. For $C_{73}H_{109}N_{15}O_{11}$ $(M+H)^{+}$: 1372.8, found 1373.0.

4b: 78%; ^{1}H NMR (400 MHz, $CDCl_3$) δ 8.57 (d, *J* = 7.3 Hz, 1H), 8.41 (d, *J* = 8.4 Hz, 1H), 8.28 (s, 1H), 7.73 (s, 2H), 7.64 (t, *J* = 7.9 Hz, 1H), 7.34 (s, 7H), 7.15 (s, 1H), 6.58 (d, *J* = 8.5 Hz, 1H), 5.52 (s, 4H), 4.19 - 4.12 (m, 2H), 3.89 (s, 2H), 3.70 (s, 4H), 3.55 (s, 2H), 3.30 - 3.24 (m, 16H), 2.39 (s, 8H), 1.90 - 1.66 (m, 14H), 1.42 (d, *J* = 13.1 Hz, 38H), 0.96 (t, *J* = 7.3 Hz, 3H); ^{13}C NMR (101 MHz, $CDCl_3$) δ 168.55, 164.39, 163.90, 156.20, 149.97, 144.08, 136.55, 135.81, 134.13, 130.72, 130.16, 129.45, 127.07, 124.42, 122.41, 120.11, 109.37, 103.27, 79.25, 53.12, 49.65, 46.87, 45.31, 43.90, 39.73, 39.22, 30.21, 28.38, 25.84, 25.40, 20.29, 13.78; IR (KBr, cm^{-1}): 3379.22, 2967.17, 2923.80, 1684.94, 1646.99, 1584.64, 1413.86, 1362.35, 1248.49, EI-MS calcd. For $C_{71}H_{105}N_{15}O_{11}$ $(M+H)^{+}$: 1344.8, found 1344.9.

4c: 57%; ^{1}H NMR (400 MHz, $CDCl_3$) δ 8.61 (d, *J* = 7.1 Hz, 1H), 8.54 (d, *J* = 8.0 Hz, 1H), 8.40 (d, *J* = 8.4 Hz, 1H), 7.76 - 7.72 (m, 1H), 7.41 (s, 2H), 7.31 (s, 2H), 7.24 (d, *J* = 4.0 Hz, 2H), 5.56 (s, 4H), 4.31

(t, J = 6.7 Hz, 1H), 4.20 - 4.15 (m, 2H), 4.07 (s, 1H), 3.79 (s, 4H), 3.31 (s, 18H), 2.44 - 2.42 (m, 8H), 1.88 - 1.83 (m, 12H), 1.73 - 1.67 (m, 4H), 1.44 (s, 38H), 1.28 - 1.21 (m, 2H), 0.98 (d, J = 7.3 Hz, 3H); ^{13}C NMR (101 MHz, D_2O) δ 166.26, 165.02, 161.60, 161.15, 153.66, 152.17, 141.72, 134.54, 134.26, 129.71, 129.66, 128.53, 128.27, 127.13, 126.99, 126.19, 125.60, 124.01, 123.59, 120.82, 120.26, 115.24, 112.96, 76.67, 62.88, 50.54, 50.39, 47.10, 44.28, 42.82, 41.33, 37.45, 27.93, 27.61, 25.87, 24.75, 23.46, 17.73, 16.54, 11.21, 11.08; IR (KBr, cm^{-1}): 3343.72, 3127.11, 2969.88, 2926.51, 1690.36, 1657.83, 1590.06 1416.57, 1365.06, 1229.52, 1161.75, 784.94; EI-MS calcd. For $C_{73}H_{107}N_{15}O_{11}$ $(M+H)^+$: 1370.8, found 1370.8.

7d: 42%; ^{1}H NMR (400 MHz, $CDCl_3$) δ 8.58 (d, J = 7.0 Hz, 1H), 8.45 (d, J = 8.0 Hz, 1H), 8.25 (d, J = 8.3 Hz, 1H), 7.63 - 7.59 (m, 3H), 7.38 (s, 2H), 7.32 (s, 1H), 6.71 (d, J = 8.1 Hz, 1H), 6.61 (s, 1H), 5.76 (s, 1H), 5.52 (s, 4H), 4.16 (s, 2H), 3.74 (s, 4H), 3.58 - 3.10 (m, 20H), 2.41 (s, 8H), 1.87 - 1.39 (m, 60H), 0.97 (t, J = 6.8 Hz, 3H); ^{13}C NMR (101 MHz, $CDCl_3$) δ 166.36, 164.64, 164.06, 156.26, 149.89, 136.84, 136.46, 134.41, 130.93, 129.82, 126.77, 124.38, 122.88, 120.33, 109.64, 104.04, 79.31, 53.25, 49.85, 46.97, 45.39, 43.95, 43.28, 39.85, 39.71 30.28, 29.60 , 29.38, 28.53, 27.88, 26.39, 26.29, 26.04, 20.36, 13.83; IR (KBr, cm^{-1}): 3433.43, 2929.22, 1638.86, 1579.22, 1384.04, 1362.35, 1251.20, 1167.17, 1104.82, 641.2; EI-MS calcd. For $C_{75}H_{113}N_{15}O_{11}$ $(M+H)^+$: 1400.8, found 1400.6.

7e: 36%; ^{1}H NMR (400 MHz, $CDCl_3$) δ 8.49 (d, J = 6.3 Hz, 1H), 8.37 (d, J = 7.6 Hz, 1H), 8.17 (d, J = 6.9 Hz, 1H), 7.60 (s, 2H), 7.52 (s, 1H), 7.27 (s, 1H), 7.20 (s, 1H), 6.62 (d, J = 8.3 Hz, 2H), 5.65 (s, 1H), 5.48 (s, 2H), 4.31 (s, 2H), 4.08 (s, 2H), 3.67 (s, 2H), 3.39 - 3.32 (m, 4H), 3.21 - 3.16 (m, 8H), 2.33 (s, 4H), 1.73 (s, 8H), 1.61 (s, 4H), 1.47 (s, 2H), 1.37 (s, 20H), 1.18 (s, 2H), 0.89 (s, 3H); ^{13}C NMR (101 MHz, $CDCl_3$) δ 166.61, 164.67, 164.11, 156.30, 149.72, 137.18, 136.56, 136.13, 134.39, 130.97, 129.98, 129.83, 126.41, 126.32, 126.90, 126.41, 126.32, 124.48, 123.02, 120.32, 109.92, 104.12, 79.40, 53.94, 53.48, 49.88, 47.22, 45.42, 44.05, 43.28, 39.90, 39.69, 30.30, 29.63, 29.47, 28.55, 28.45, 26.36, 26.25, 20.39, 13.83; IR (KBr, cm^{-1}): 3438.86, 2926.51, 2099.70, 1638.86, 1579.22, 1549.40, 1384.04, 1359.64, 1248.49, 1164.46, 1115.66, 779.52; EI-MS calcd. For $C_{53}H_{74}N_{12}O_7$ $(M+H)^+$: 991.5, found 991.3.

6. Spectra

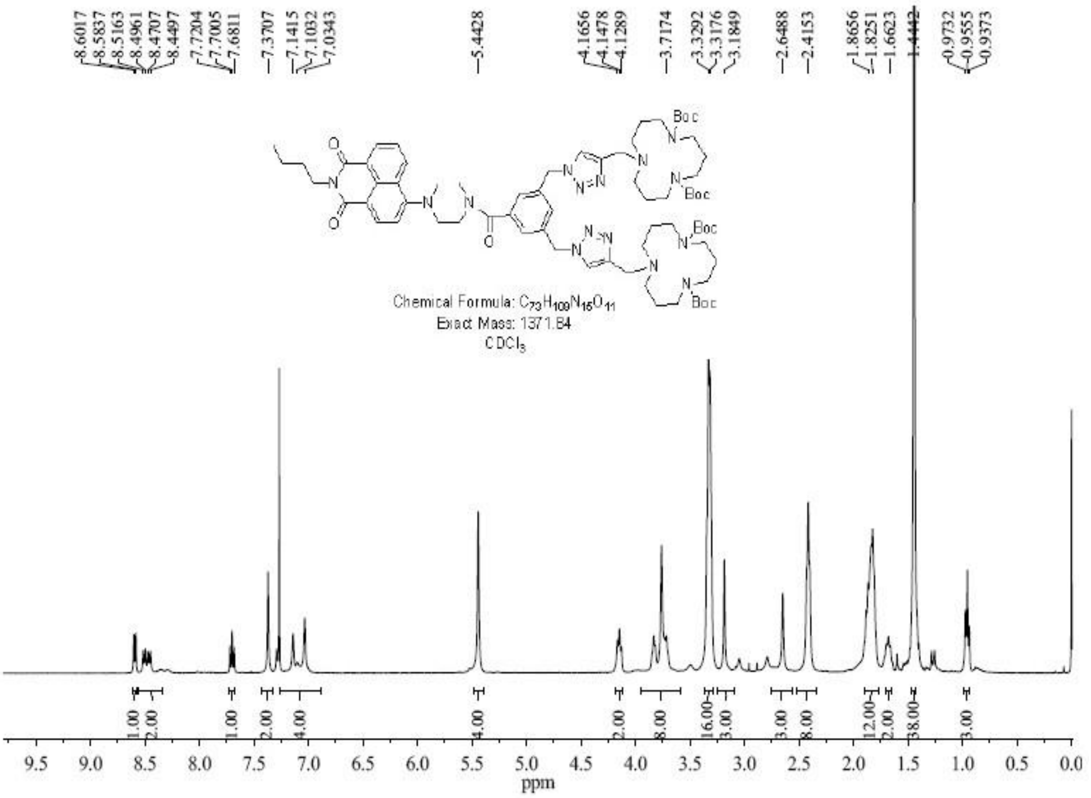

^{1}H NMR spectrum of compound **4a** (solvent: $CDCl_3$)

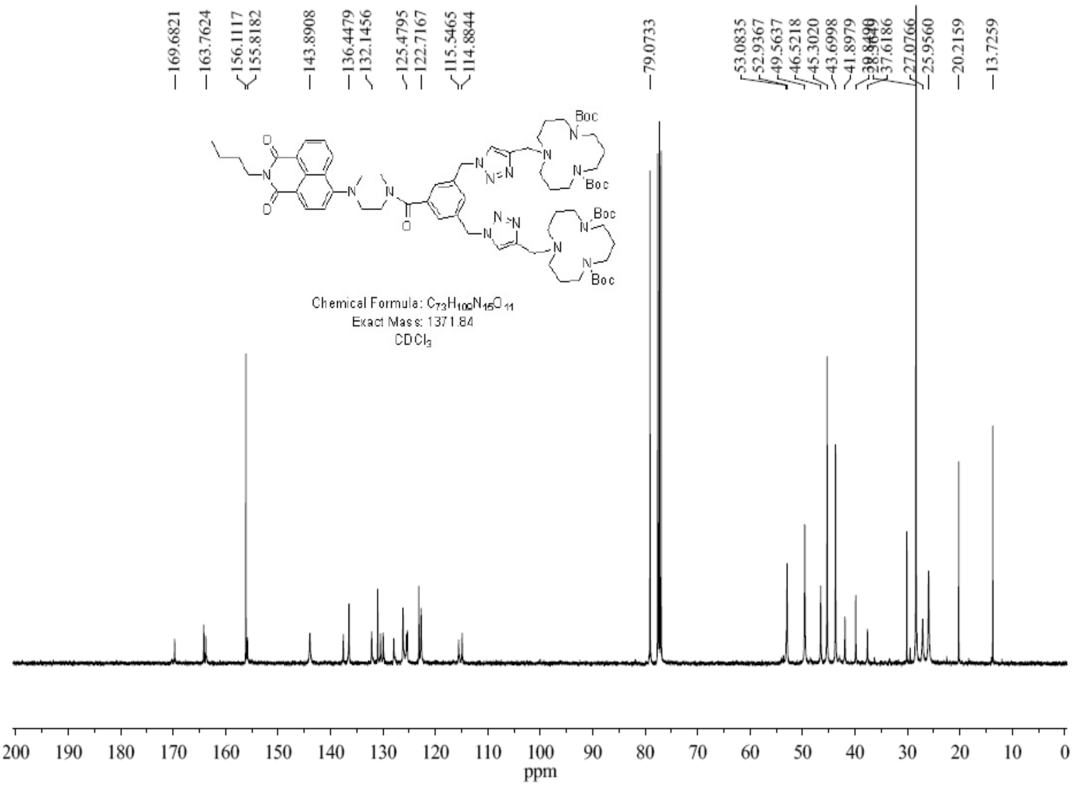

^{13}C NMR spectrum of compound **4a** (solvent: $CDCl_3$)

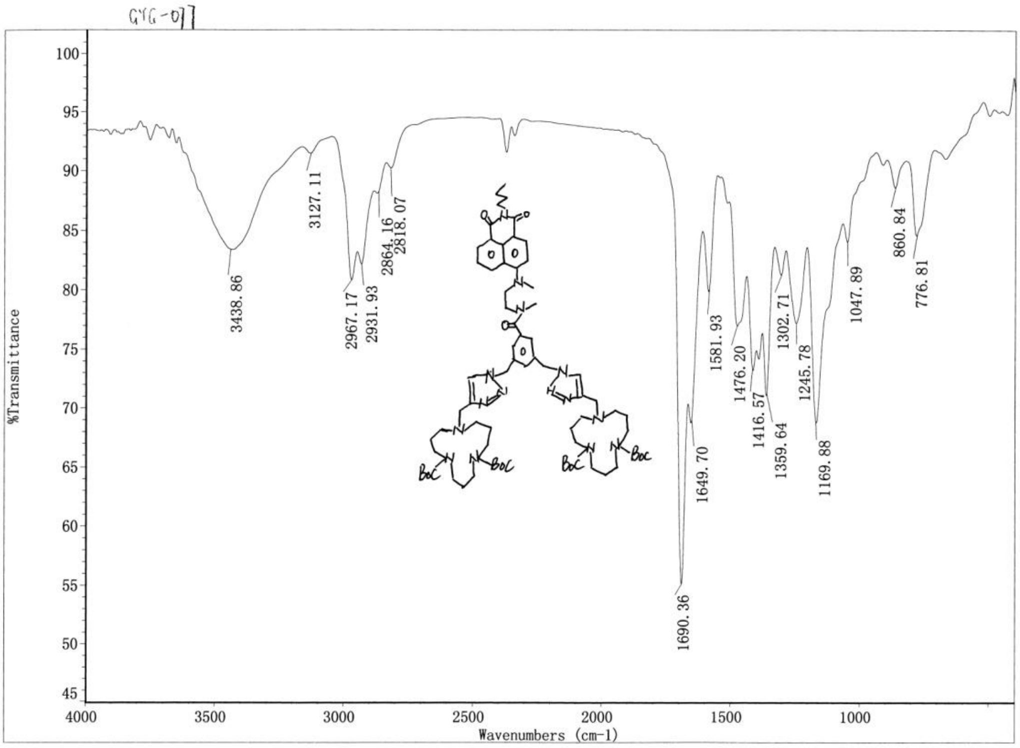

IR spectrum of compound **4a**

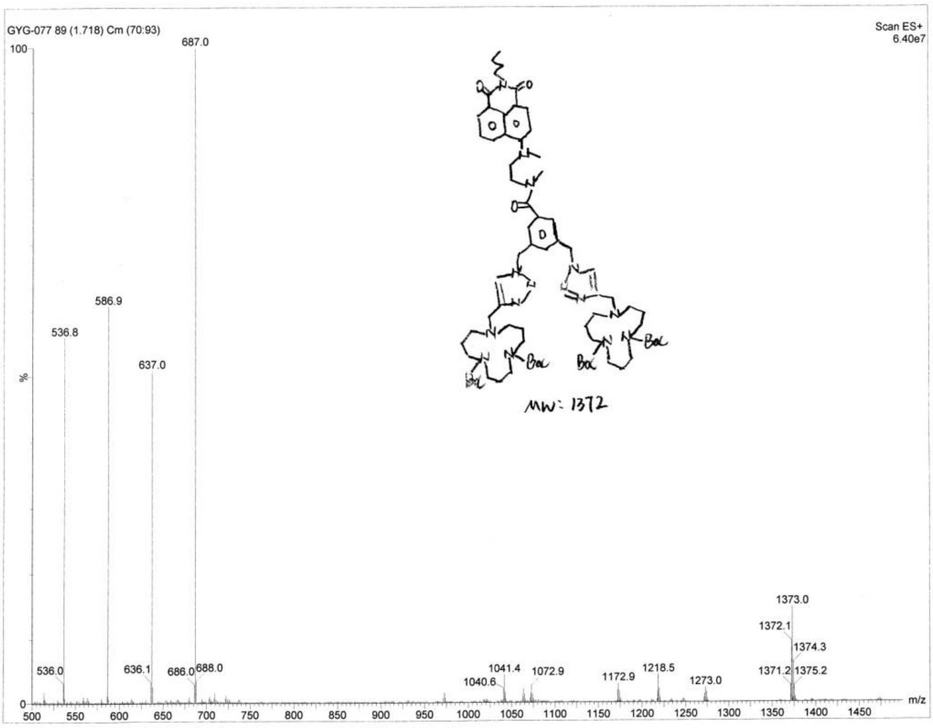

Ms spectrum of compound **4a**

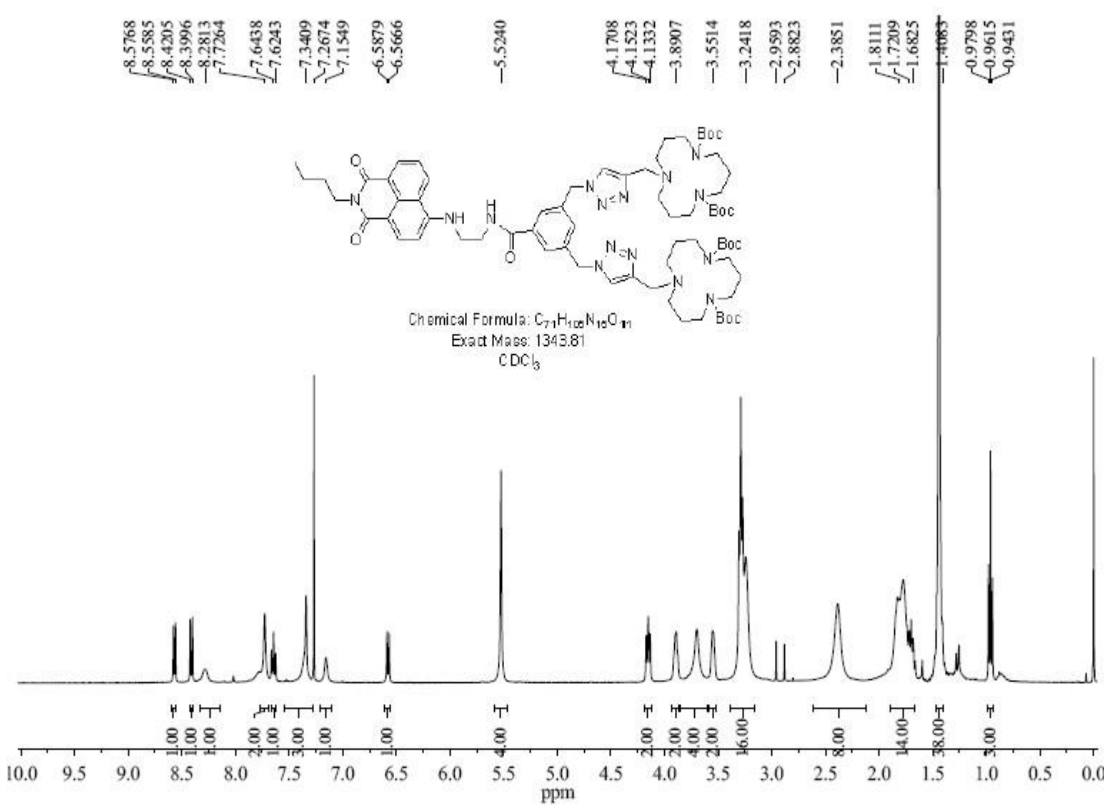

^{1}H NMR spectrum of compound **4b** (solvent: $CDCl_3$)

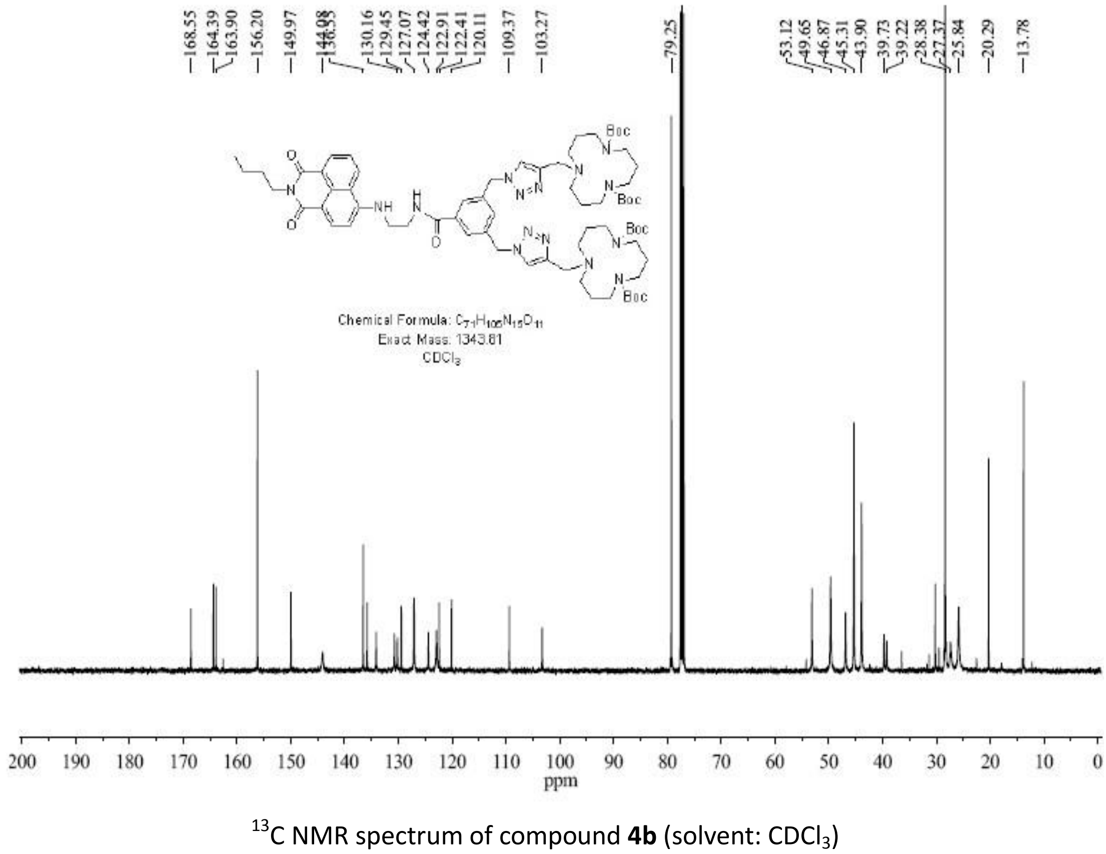

^{13}C NMR spectrum of compound **4b** (solvent: $CDCl_3$)

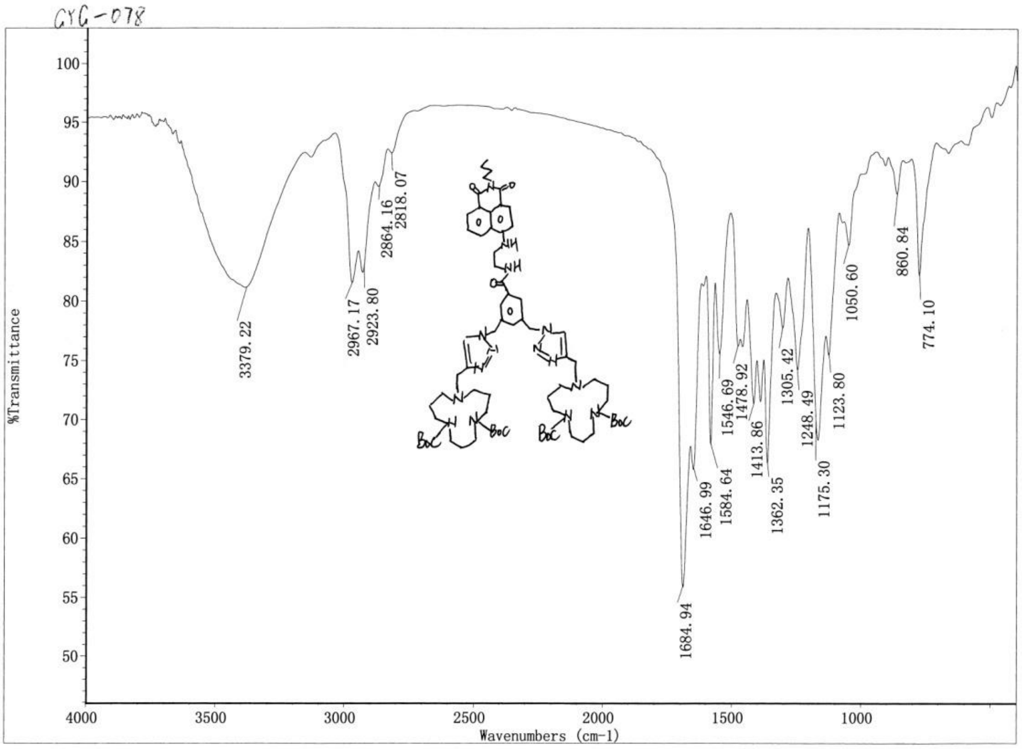

IR spectrum of compound **4b**

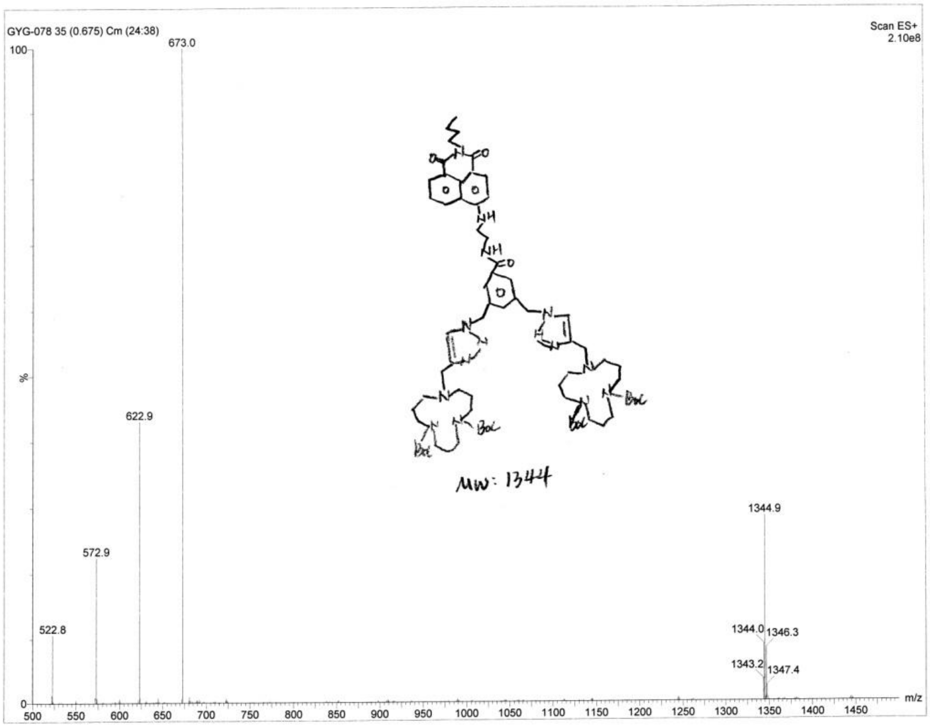

Ms spectrum of compound **4b** (solvent: $CDCl_3$)

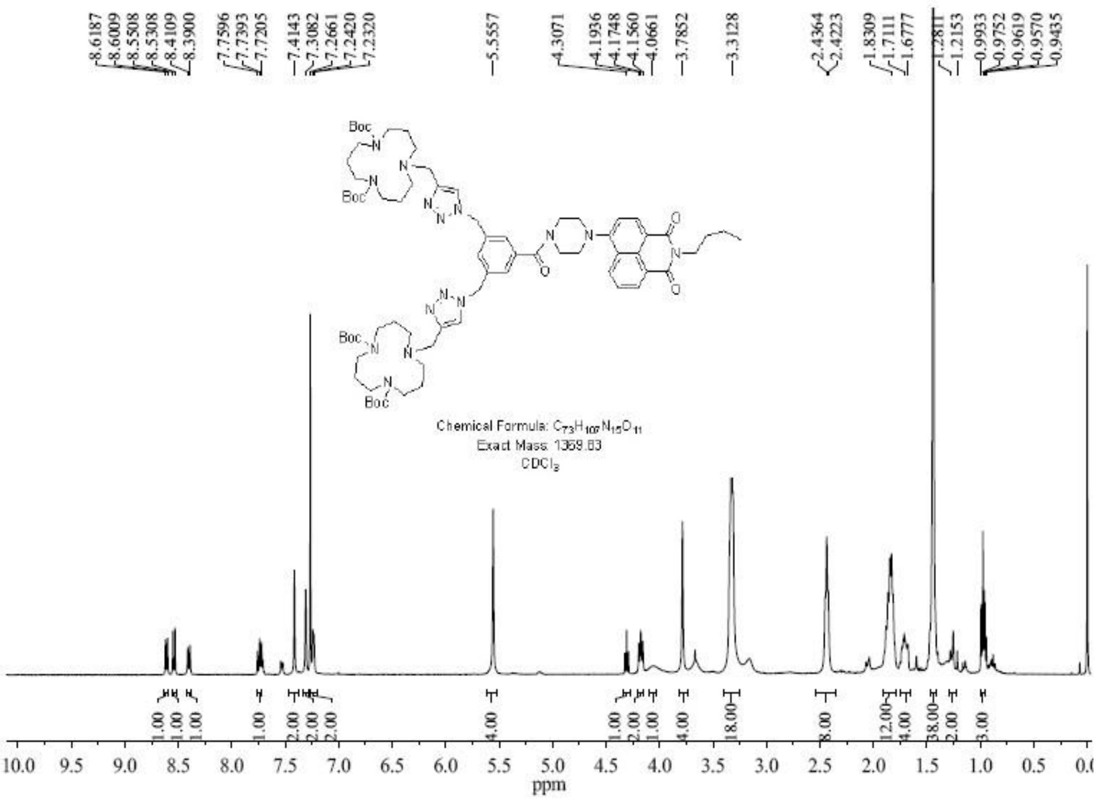

^{1}H NMR spectrum of compound **4c** (solvent: $CDCl_3$)

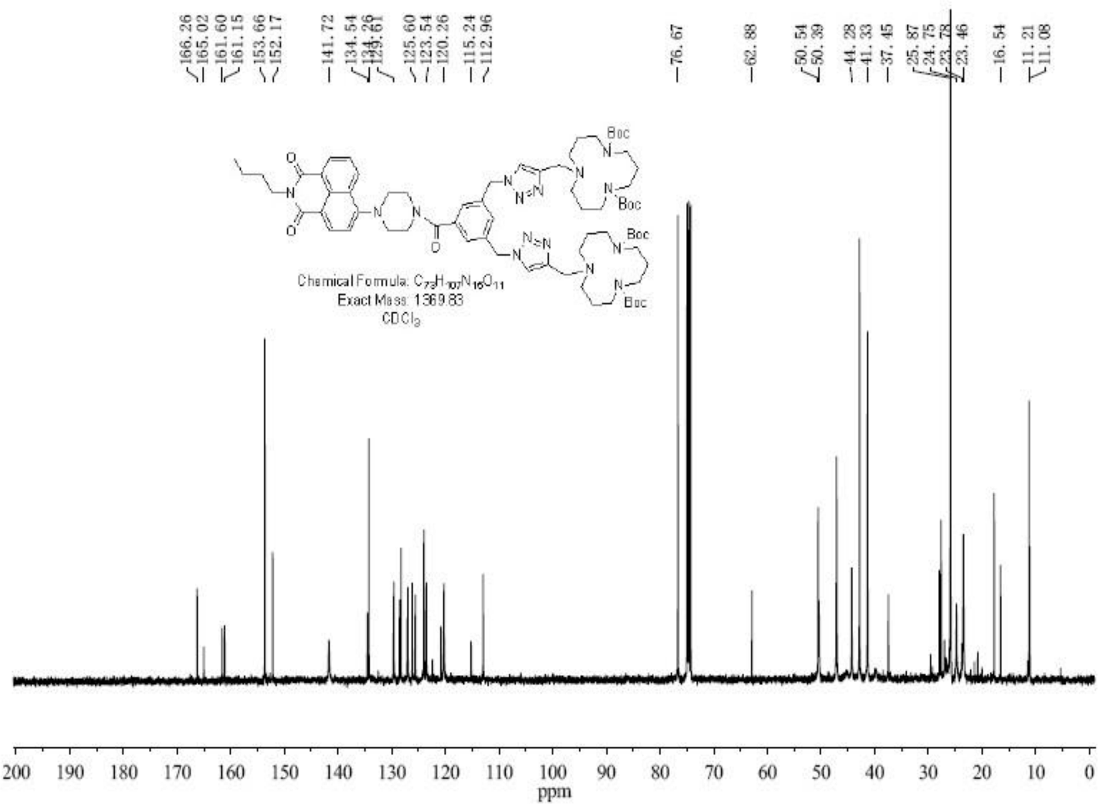

^{13}C NMR spectrum of compound **4c** (solvent: $CDCl_3$)

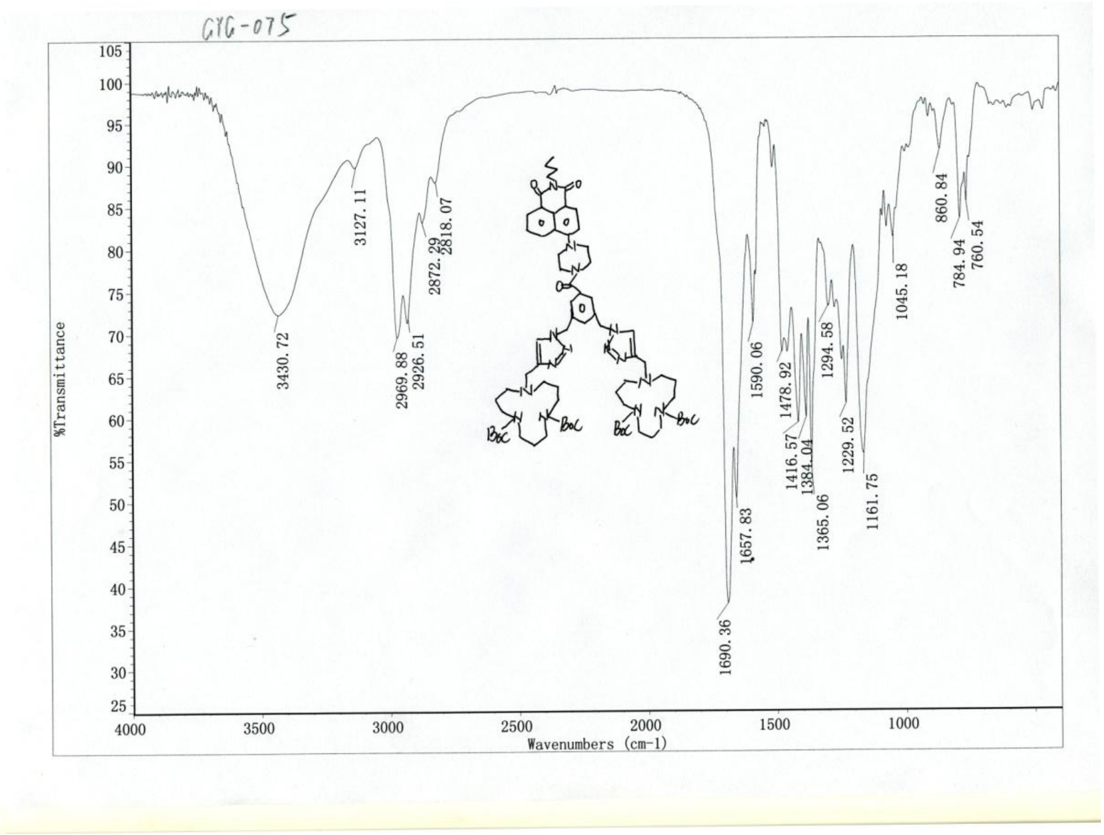

IR spectrum of compound **4c**

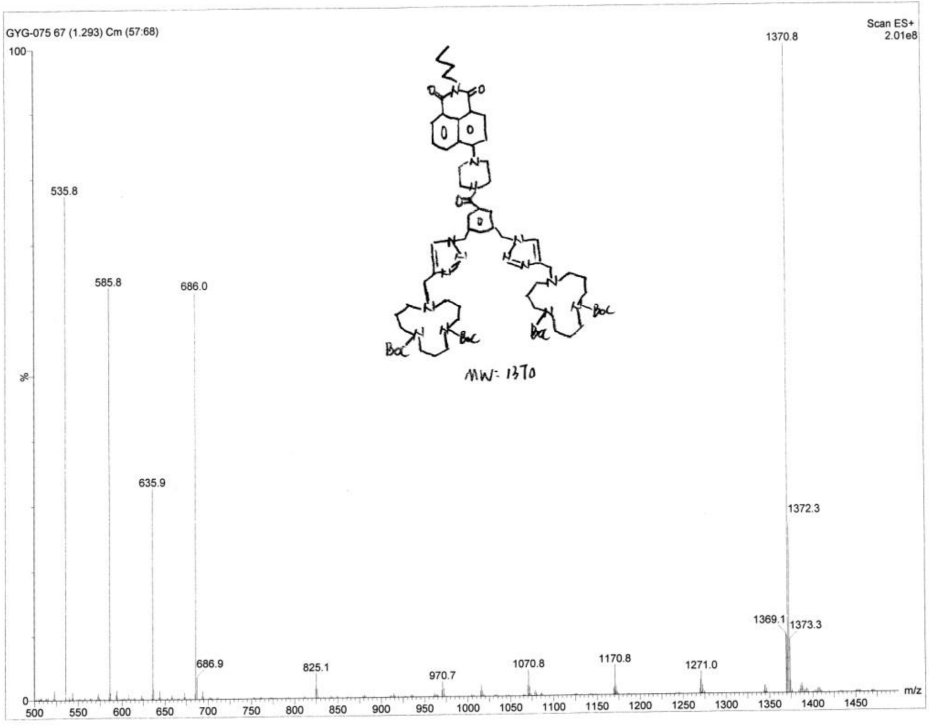

Ms spectrum of compound **4c**

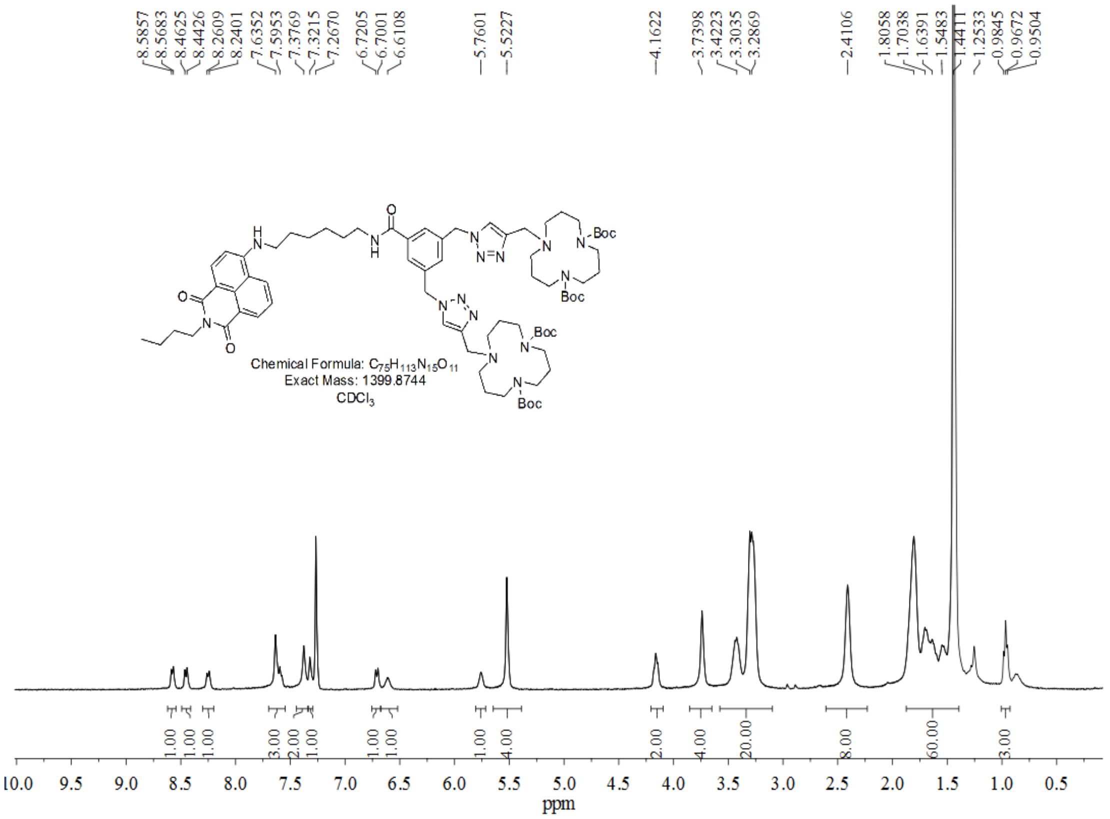

^{1}H NMR spectrum of compound **7d** (solvent: $CDCl_3$)

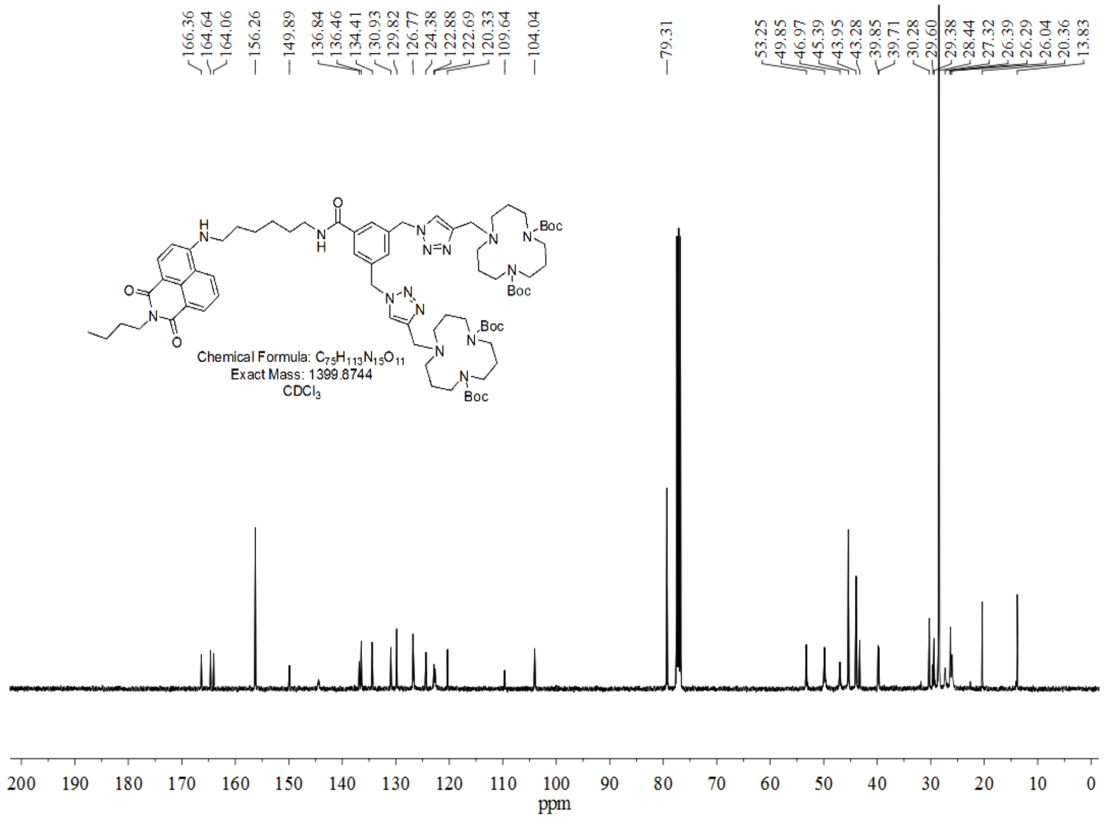

^{13}C NMR spectrum of compound **7d** (solvent: $CDCl_3$)

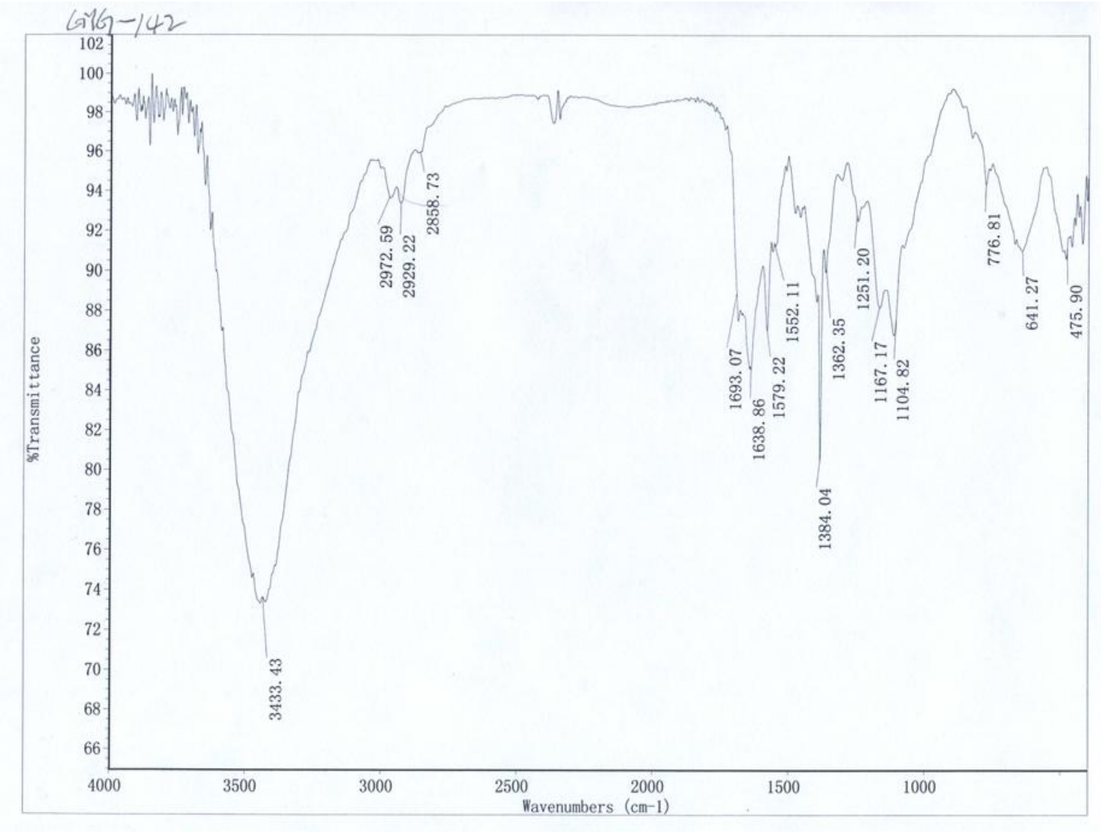

IR spectrum of compound **7d** (solvent: $CDCl_3$)

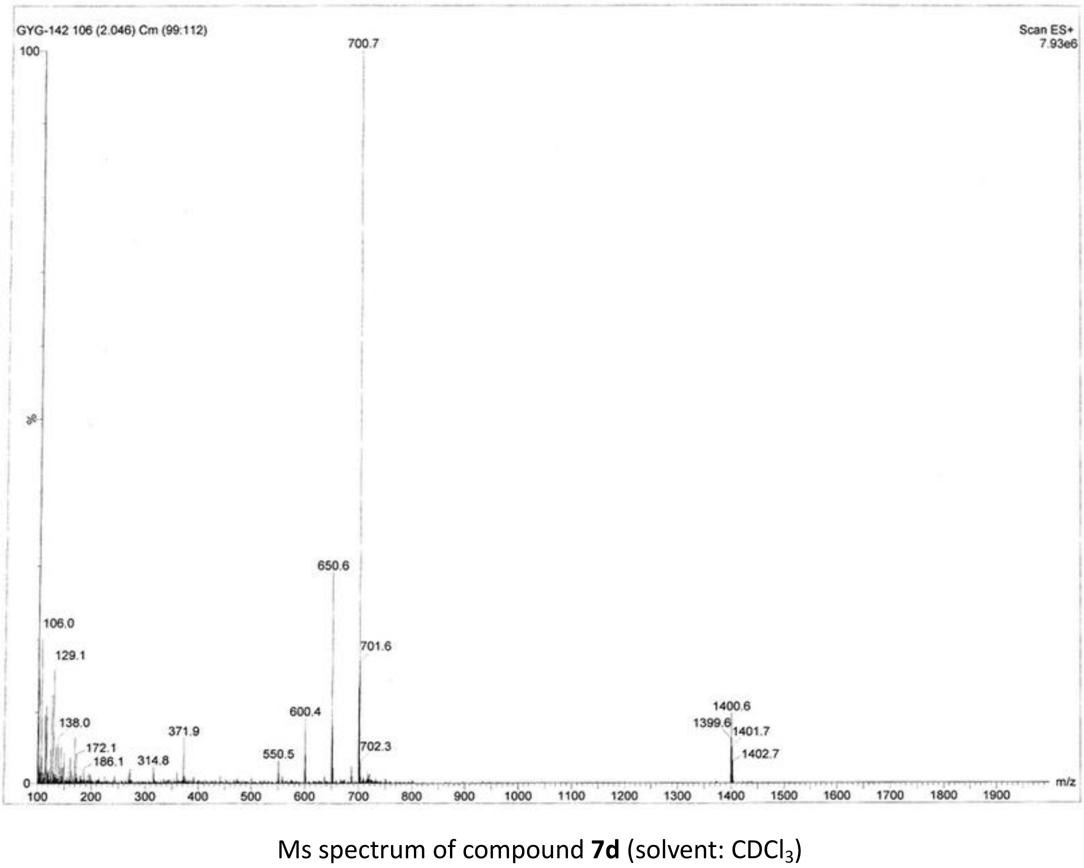

Ms spectrum of compound **7d** (solvent: $CDCl_3$)

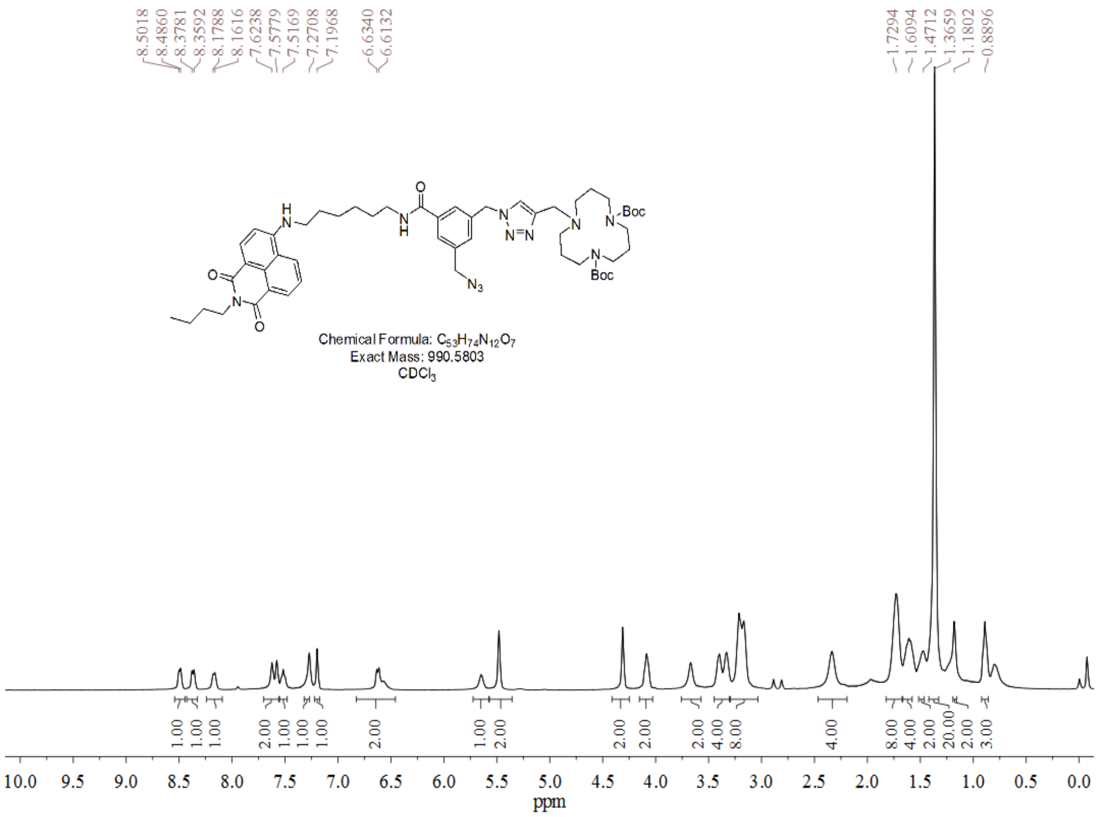

^{1}H NMR spectrum of compound **7e** (solvent: $CDCl_3$)

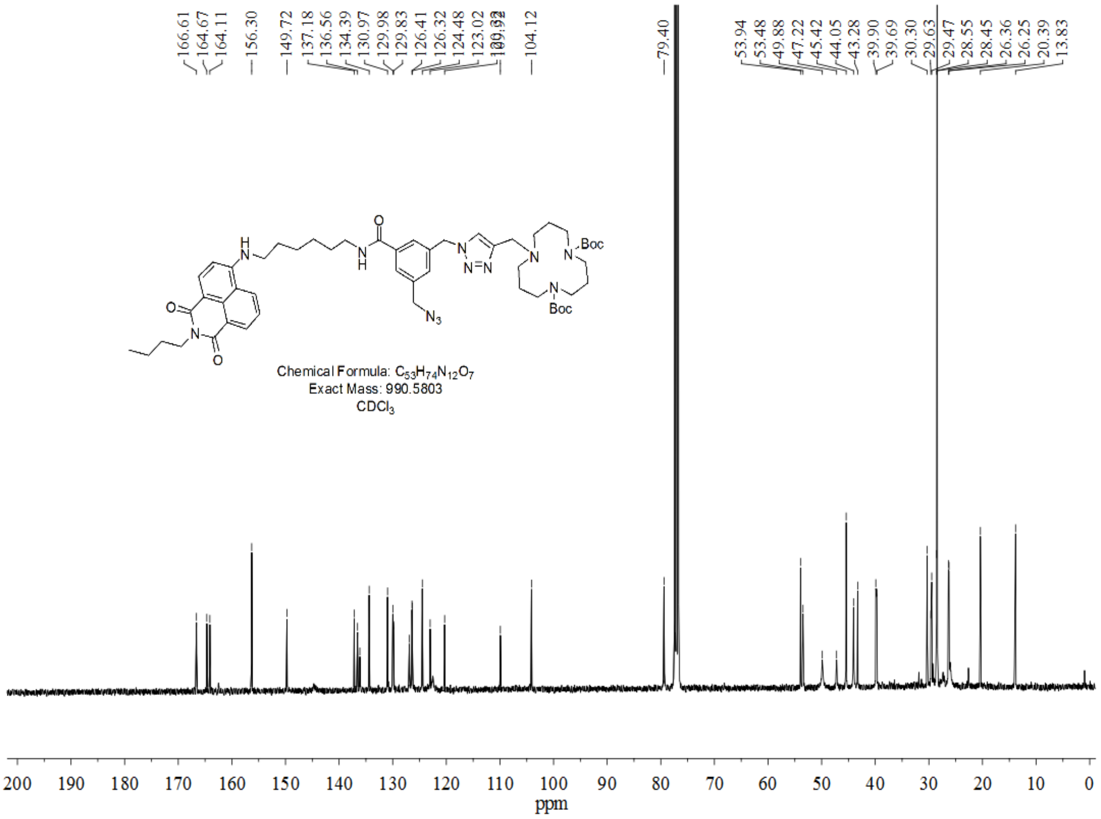

^{13}C NMR spectrum of compound **7e** (solvent: $CDCl_3$)

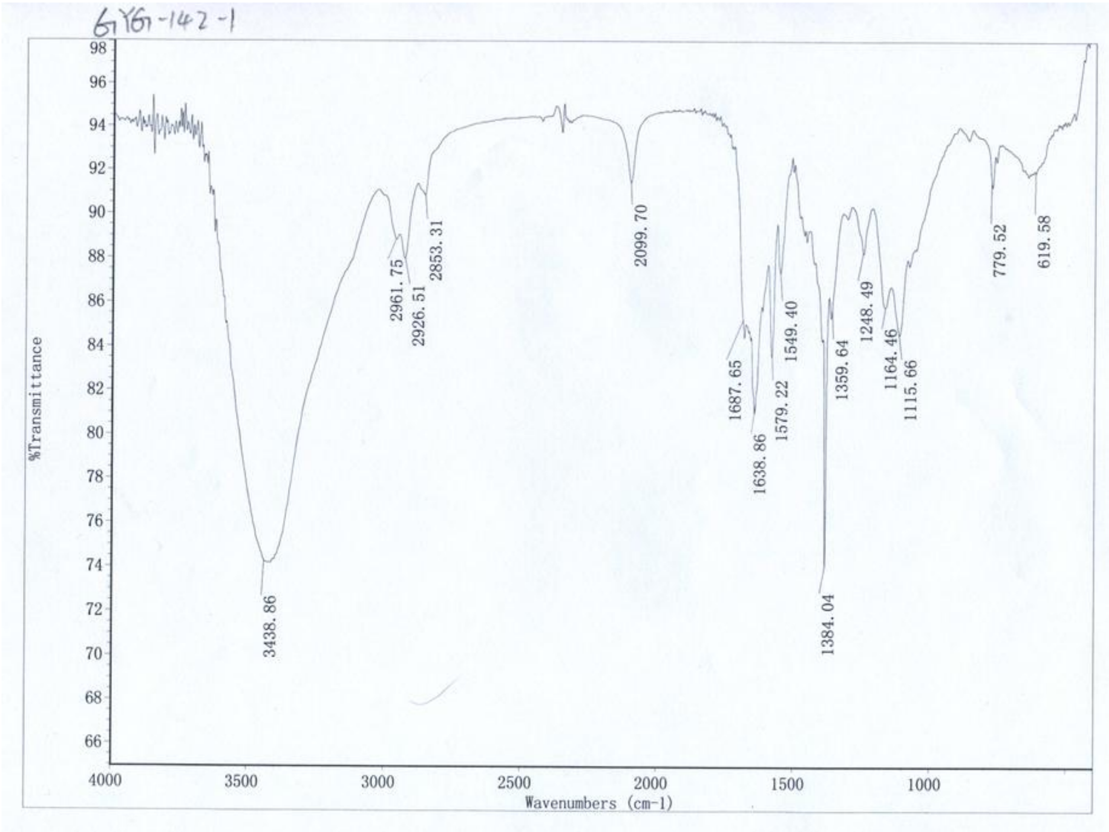

IR spectrum of compound **7e** (solvent: $CDCl_3$)

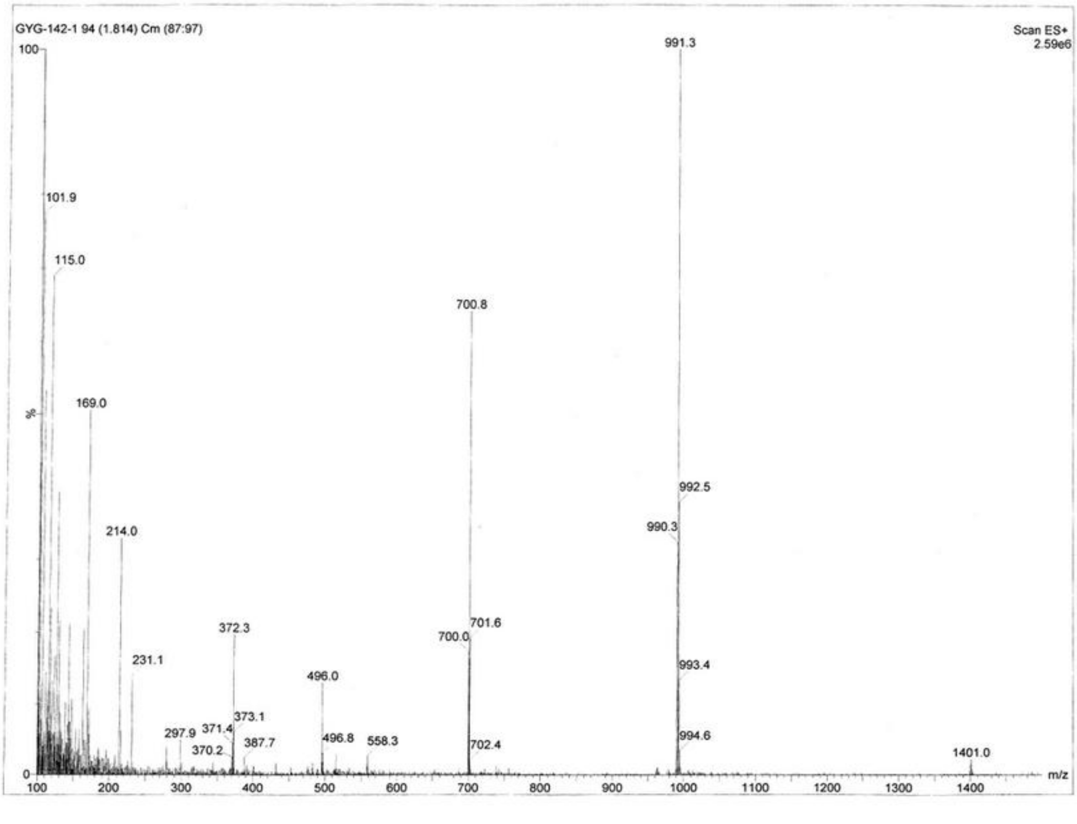

Ms spectrum of compound **7e** (solvent: $CDCl_3$)

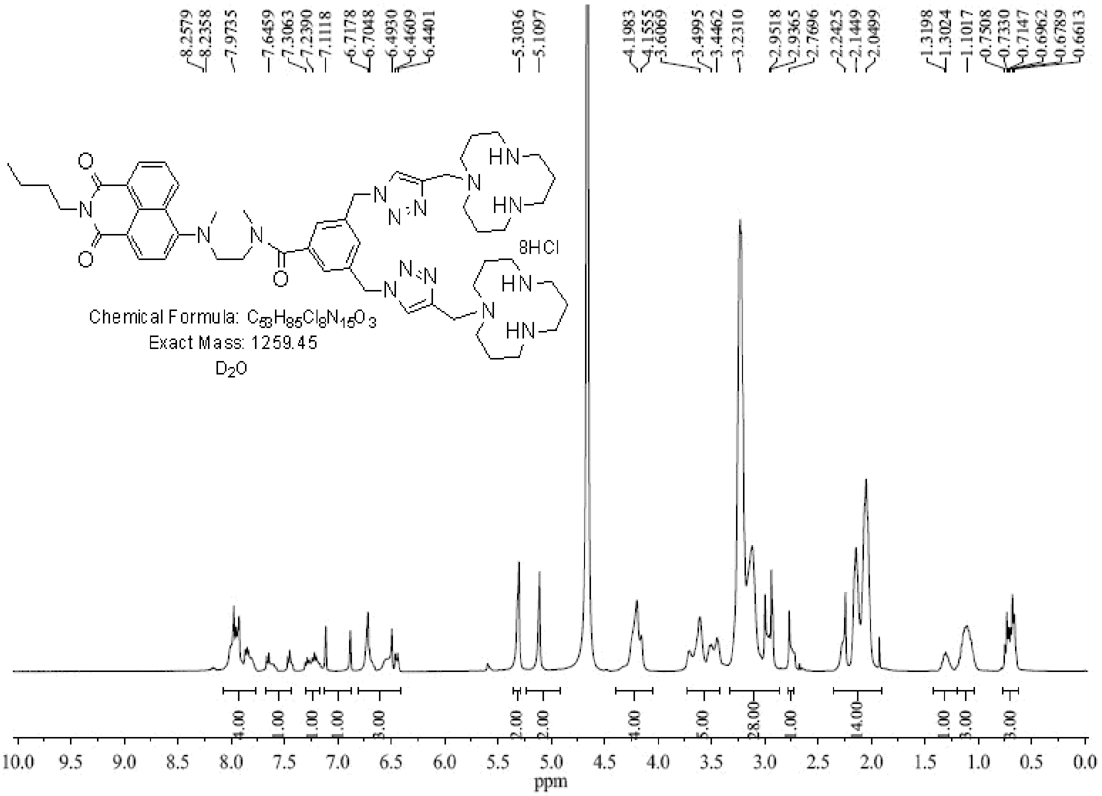

^{1}H NMR spectrum of compound **1a** (solvent: D_2O)

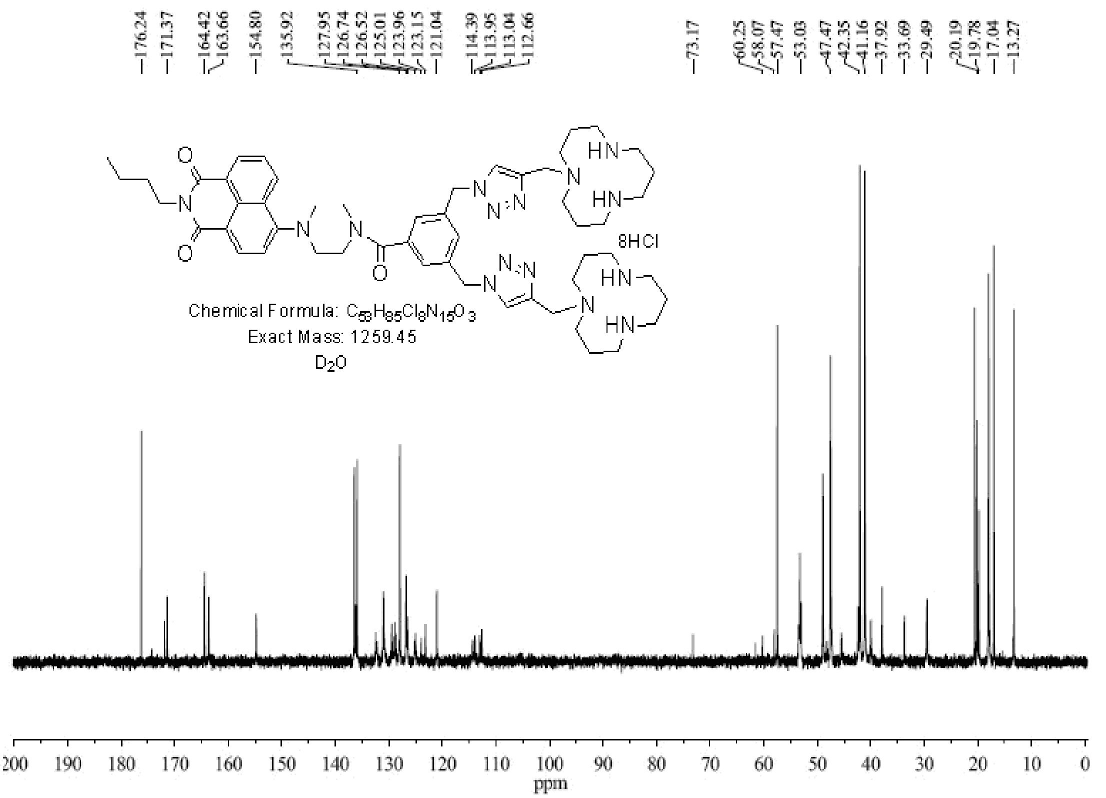

^{13}C NMR spectrum of compound **1a** (solvent: D_2O)

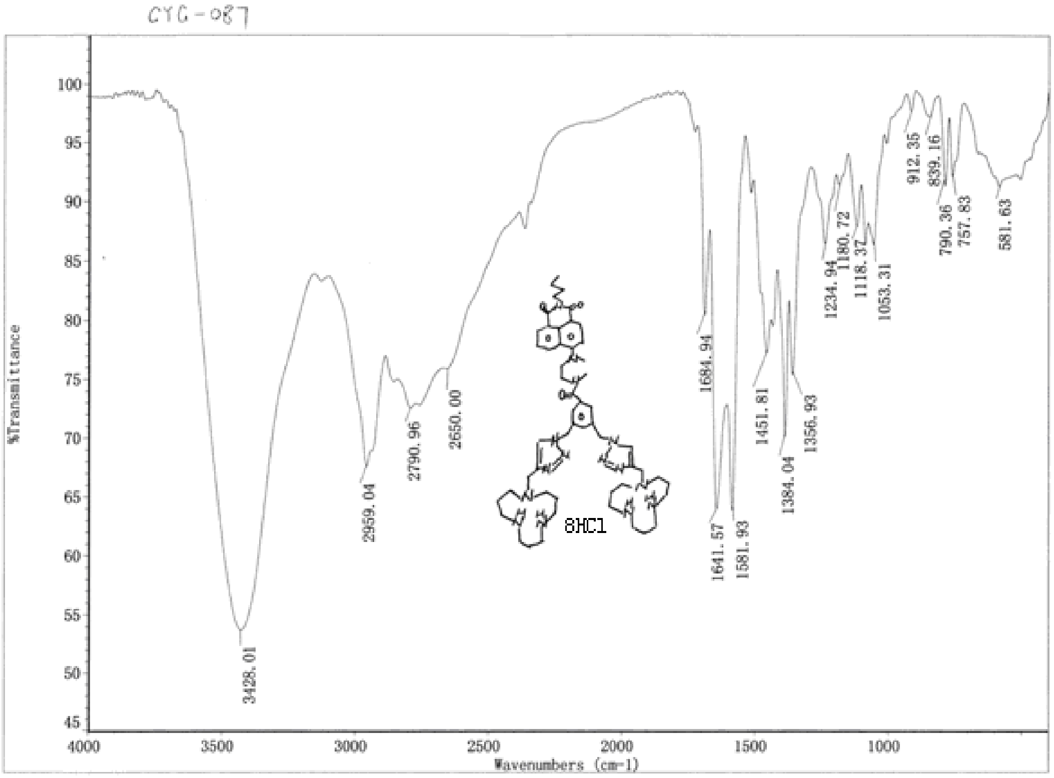

IR spectrum of compound **1a**

Elemental Composition Report **Page 1**

Single Mass Analysis
Tolerance = 3.0 PPM / DBE: min = -1.5, max = 50.0
Element prediction: Off
Number of isotope peaks used for i-FIT = 2

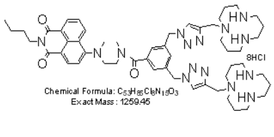

Monoisotopic Mass, Even Electron Ions
535 formula(e) evaluated with 2 results within limits (up to 50 closest results for each mass)
Elements Used:
C: 0-60 H: 0-80 N: 0-15 O: 0-10
GYG087 28 (0.518)
TOF MS ES+

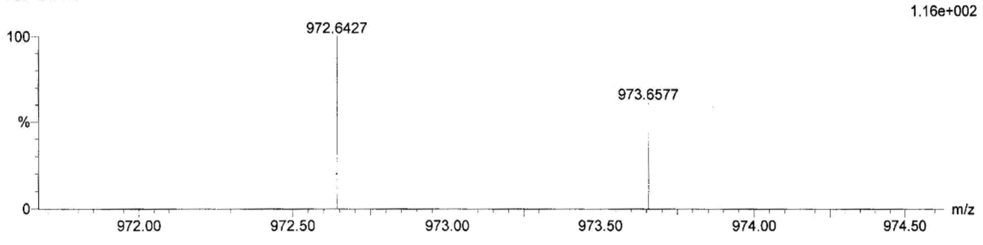

Minimum:				-1.5		
Maximum:		5.0	3.0	50.0		
Mass	Calc. Mass	mDa	PPM	DBE	i-FIT	Formula
972.6427	972.6412	1.5	1.5	22.5	0.2	C53 H78 N15 O3
	972.6452	-2.5	-2.6	26.5	0.5	C58 H78 N13 O

HRMs spectrum of compound **1a**

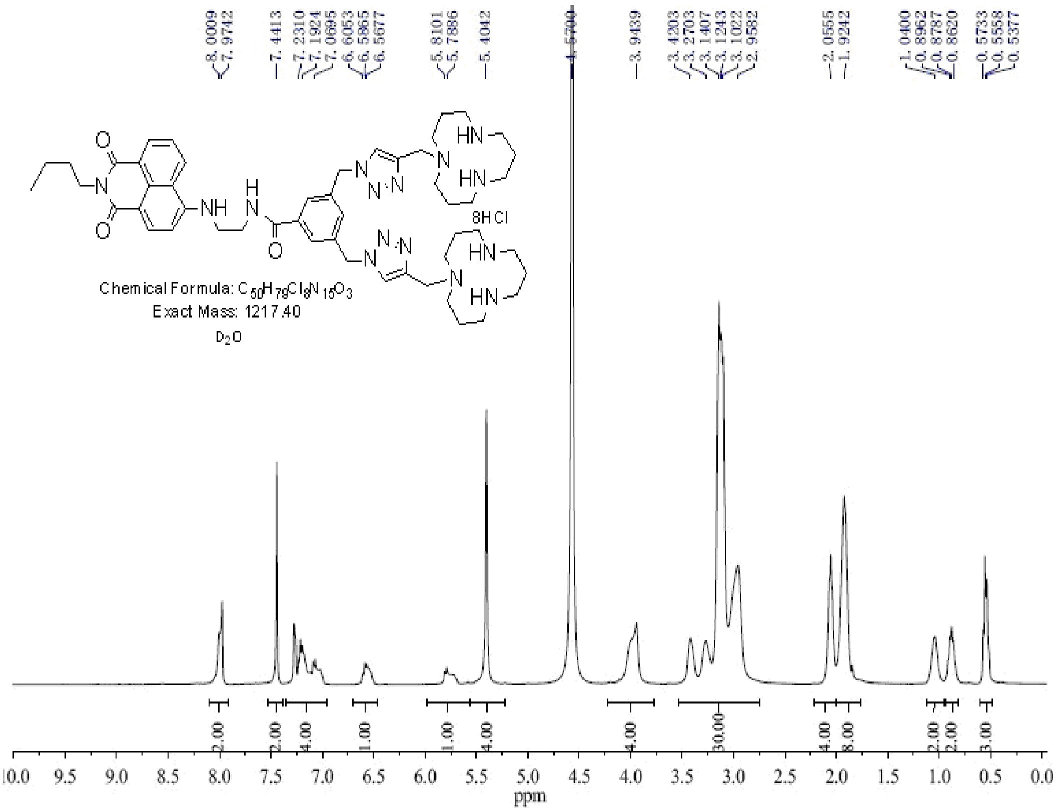

^{1}H NMR spectrum of compound **1b** (solvent: D_2O)

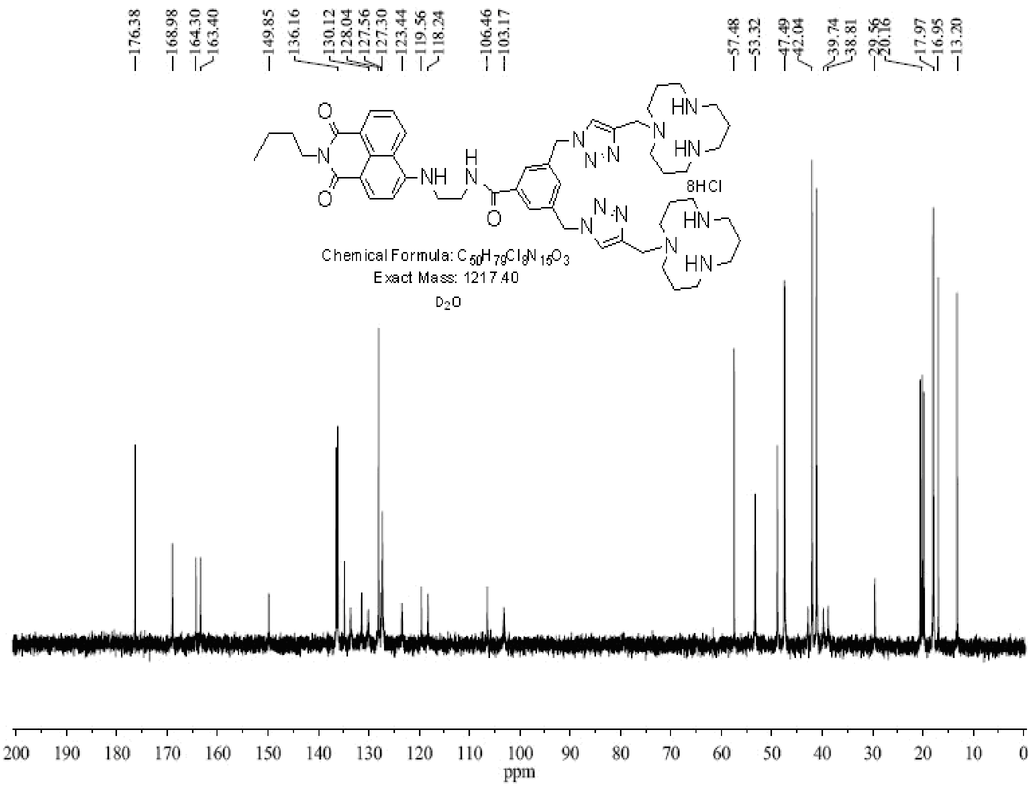

^{13}C NMR spectrum of compound **1b** (solvent: D_2O)

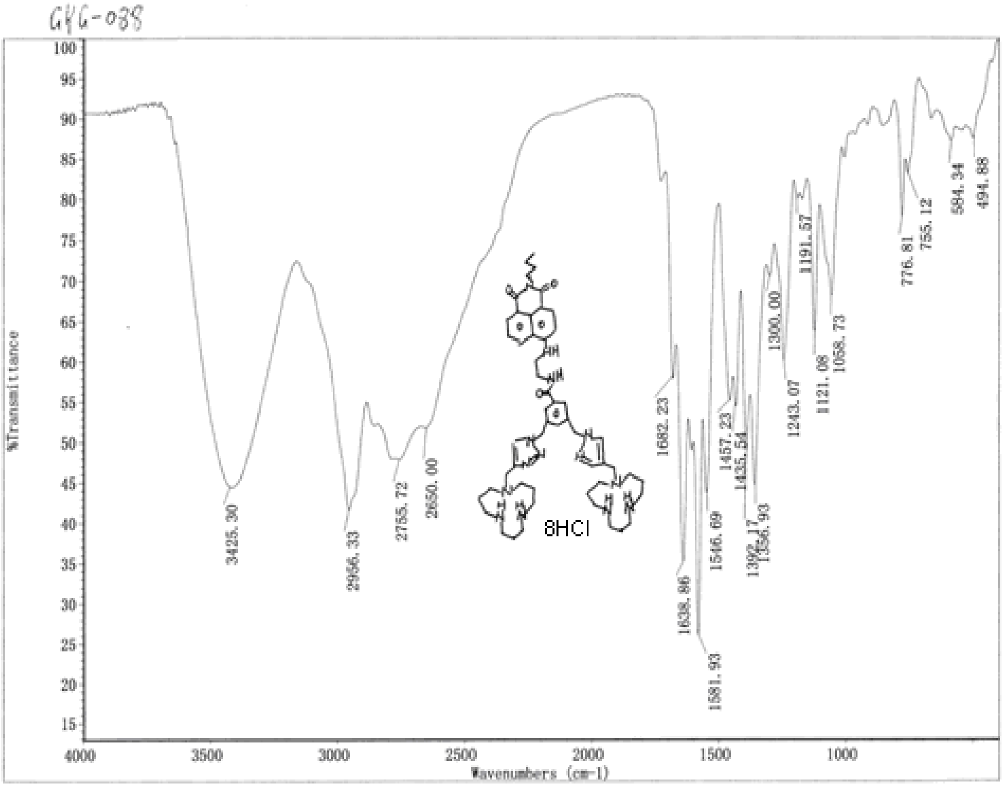

IR spectrum of compound **1b**

Elemental Composition Report **Page 1**

Single Mass Analysis
Tolerance = 5.0 PPM / DBE: min = -1.5, max = 50.0
Element prediction: Off
Number of isotope peaks used for i-FIT = 2

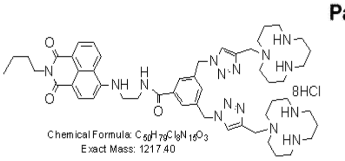

Monoisotopic Mass, Even Electron Ions
671 formula(e) evaluated with 4 results within limits (up to 50 closest results for each mass)
Elements Used:
C: 0-60 H: 0-80 N: 0-15 O: 0-10
GYG088 46 (0.851)
TOF MS ES+

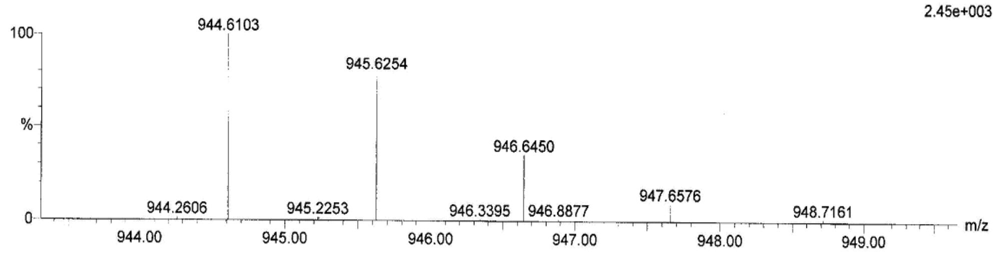

Minimum:				-1.5		
Maximum:		5.0	5.0	50.0		
Mass	Calc. Mass	mDa	PPM	DBE	i-FIT	Formula
944.6103	944.6099	0.4	0.4	22.5	26.7	C51 H74 N15 O3
	944.6086	1.7	1.8	17.5	36.0	C50 H78 N11 O7
	944.6126	-2.3	-2.4	21.5	18.7	C55 H78 N9 O5
	944.6139	-3.6	-3.8	26.5	12.5	C56 H74 N13 O

HRMs spectrum of compound **1b**

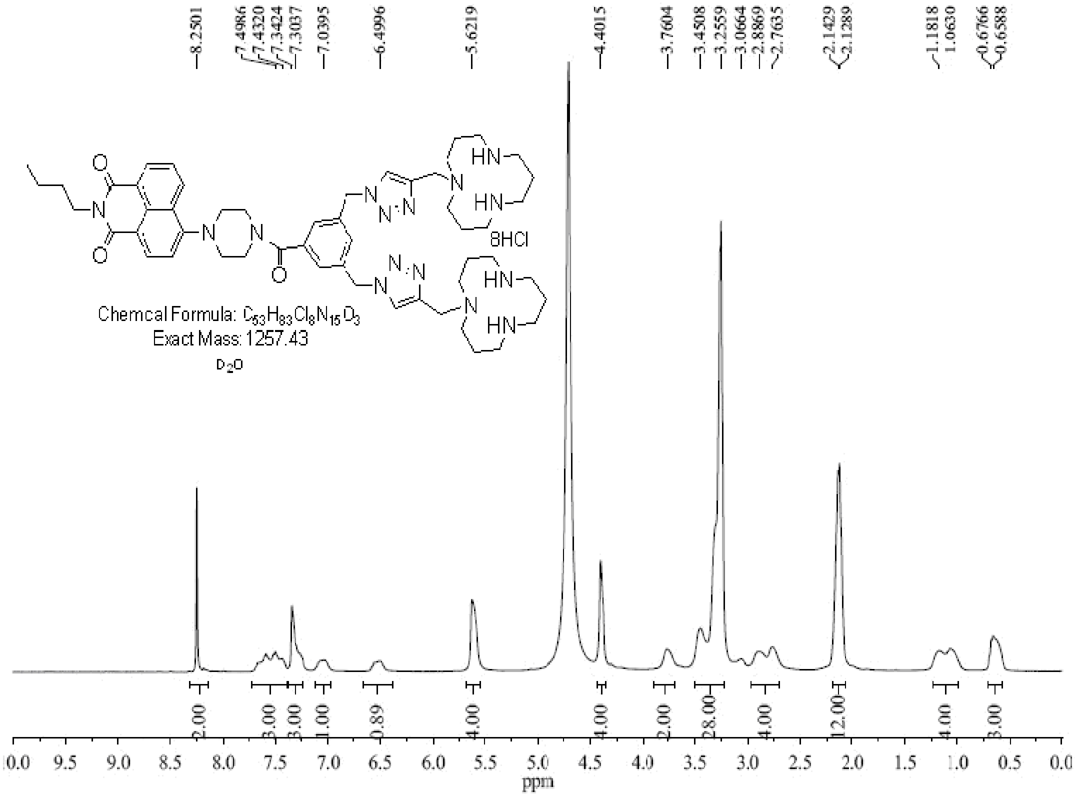

^{1}H NMR spectrum of compound **1c** (solvent: D_2O)

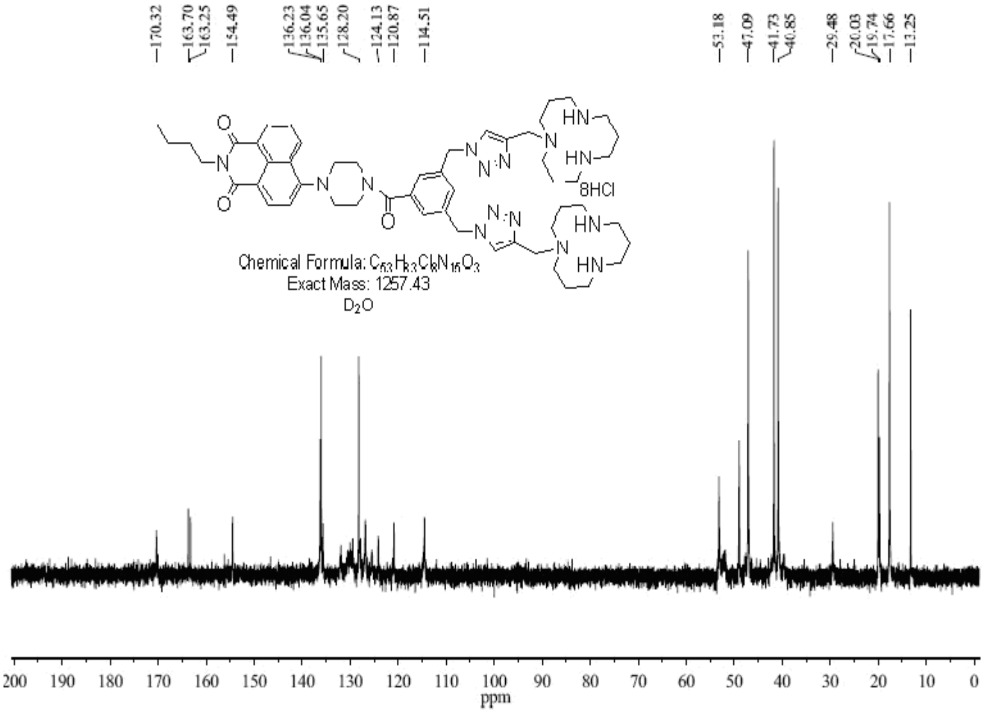

^{13}C NMR spectrum of compound **1c** (solvent: D_2O)

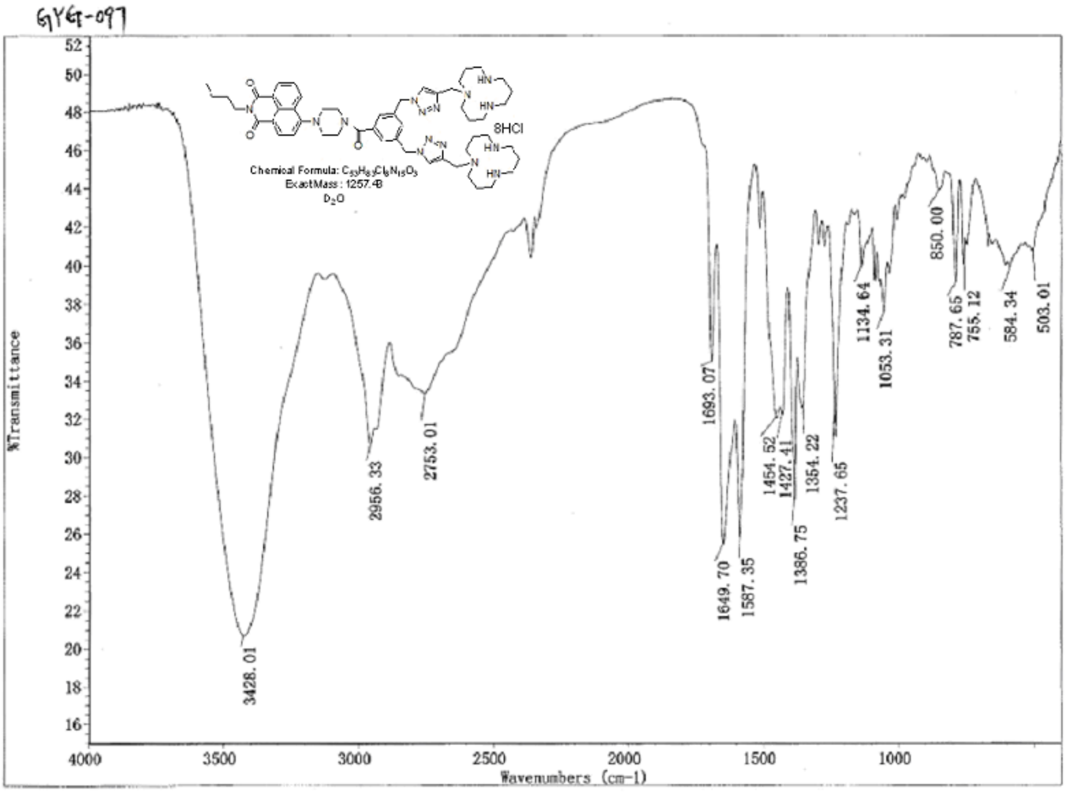

IR spectrum of compound **1c**

Elemental Composition Report **Page 1**

Single Mass Analysis
Tolerance = 5.0 PPM / DBE: min = -1.5, max = 50.0
Element prediction: Off
Number of isotope peaks used for i-FIT = 2

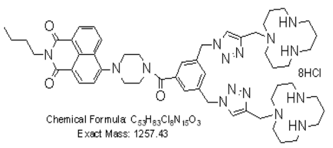

Monoisotopic Mass, Even Electron Ions
546 formula(e) evaluated with 4 results within limits (up to 50 closest results for each mass)
Elements Used:
C: 0-60 H: 0-80 N: 0-15 O: 0-10
GYG097 12 (0.222)
TOF MS ES+

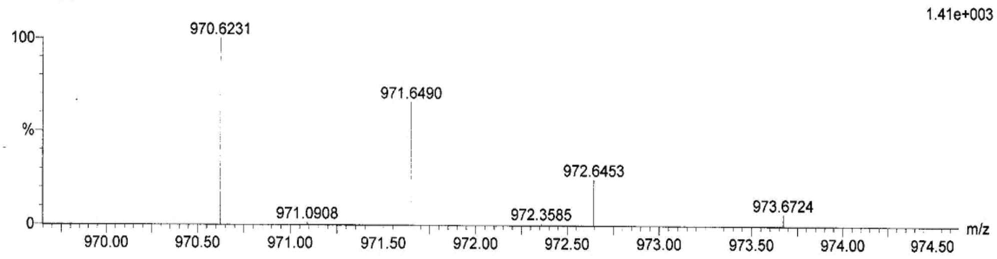

Mass	Calc. Mass	mDa	PPM	DBE	i-FIT	Formula
Minimum:				-1.5		
Maximum:		5.0	5.0	50.0		
970.6231	970.6242	-1.1	-1.1	18.5	1.6	C52 H80 N11 O7
	970.6256	-2.5	-2.6	23.5	1.2	C53 H76 N15 O3
	970.6202	2.9	3.0	14.5	5.3	C47 H80 N13 O9
	970.6183	4.8	4.9	27.5	3.9	C59 H76 N11 O2

HRMs spectrum of compound **1c**

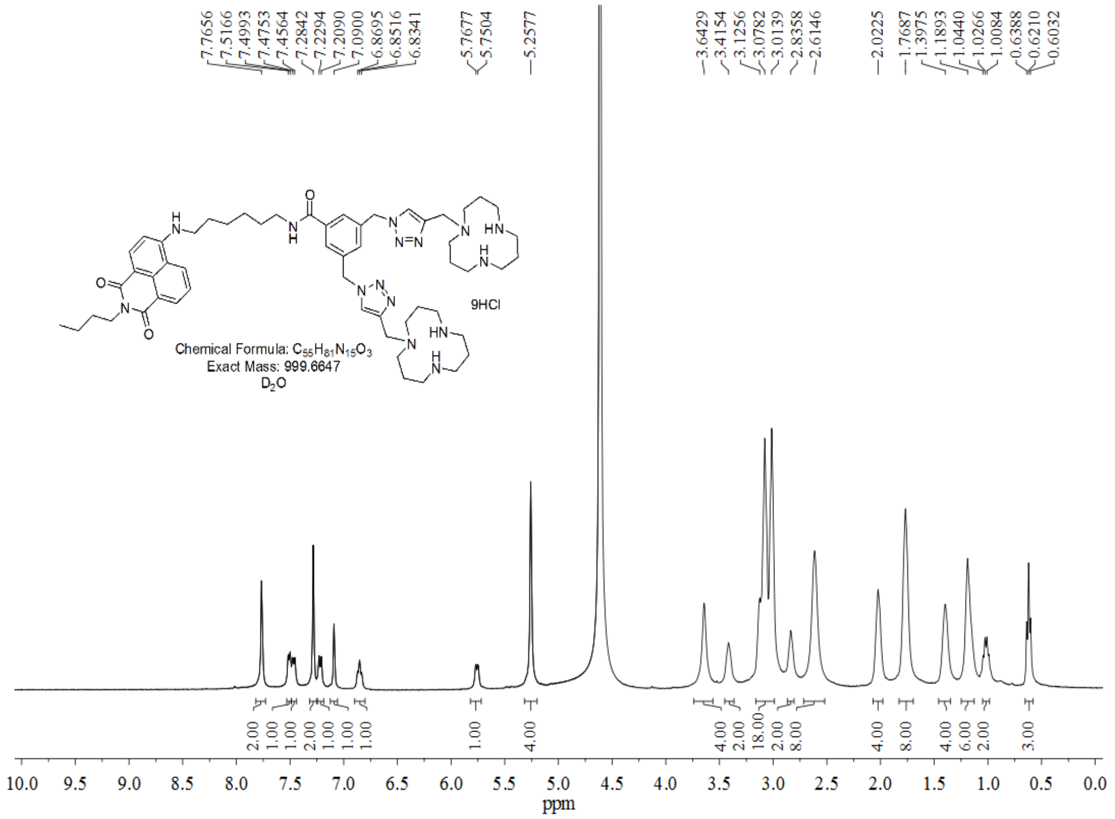

^{1}H NMR spectrum of compound **1d** (solvent: D_2O)

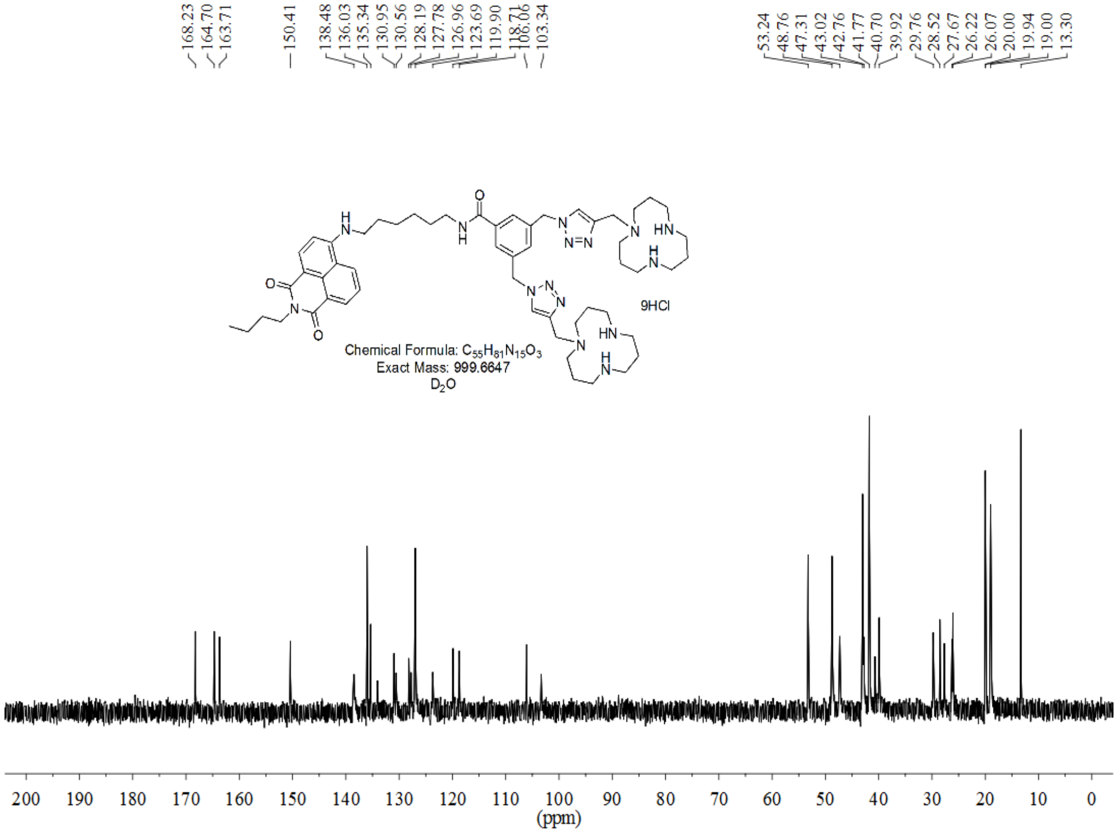

^{13}C NMR spectrum of compound **1d** (solvent: D_2O)

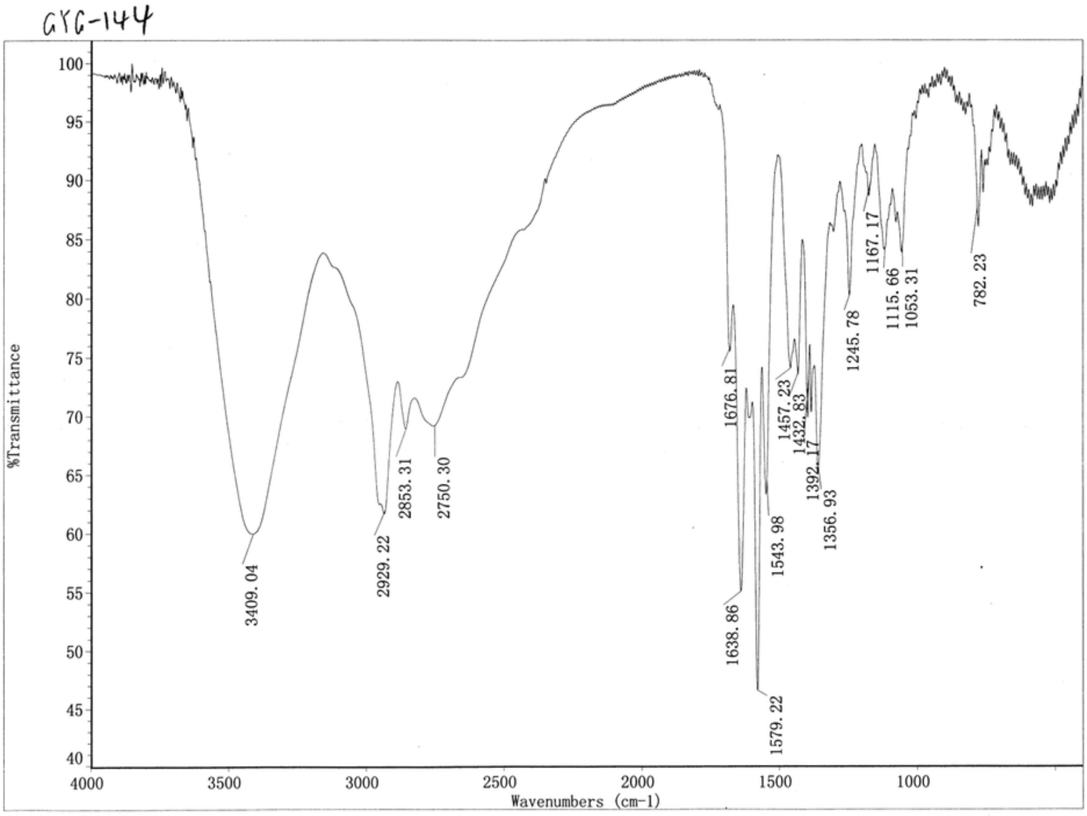

IR spectrum of compound **1d**

Elemental Composition Report **Page 1**

Single Mass Analysis
Tolerance = 5.0 PPM / DBE: min = -1.5, max = 50.0
Element prediction: Off
Number of isotope peaks used for i-FIT = 2

Monoisotopic Mass, Even Electron Ions
6941 formula(e) evaluated with 27 results within limits (up to 50 closest results for each mass)
Elements Used:
C: 0-100 H: 0-120 N: 0-15 O: 0-10 I: 0-4

GYG-144 11 (0.204)
TOF MS ES+

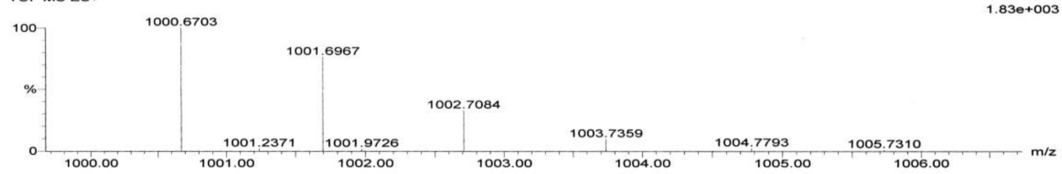

Mass	Calc. Mass	mDa	PPM	DBE	i-FIT	Formula
Minimum:				-1.5		
Maximum:		5.0	5.0	50.0		
1000.6703	1000.6704	-0.1	-0.1	10.5	14.2	C53 H95 N9 O I
	1000.6712	-0.9	-0.9	17.5	10.4	C54 H86 N11 O7
	1000.6712	-0.9	-0.9	21.5	239.2	C24 H9 N6 O7 I4
	1000.6693	1.0	1.0	34.5	119.4	C36 H5 N4 I4
	1000.6693	1.0	1.0	30.5	2.5	C66 H82 N9
	1000.6691	1.2	1.2	5.5	19.9	C52 H99 N5 O5 I
	1000.6687	1.6	1.6	36.5	122.8	C36 N2 O10 I3
	1000.6720	-1.7	-1.7	29.5	2.9	C70 H86 N3 O2
	1000.6685	1.8	1.8	22.5	268.8	C20 H5 N12 O5 I4
	1000.6725	-2.2	-2.2	22.5	6.7	C55 H82 N15 O3
	1000.6725	-2.2	-2.2	26.5	212.1	C25 H5 N10 O3 I4
	1000.6680	2.3	2.3	25.5	2.5	C65 H86 N5 O4
	1000.6680	2.3	2.3	29.5	139.0	C35 H9 O4 I4
	1000.6727	-2.4	-2.4	40.5	87.8	C41 O8 I3
	1000.6678	2.5	2.5	0.5	26.9	C51 H103 N O9 I
	1000.6731	-2.8	-2.8	9.5	9.4	C57 H99 N3 O3 I
	1000.6672	3.1	3.1	17.5	299.5	C19 H9 N8 O9 I4
	1000.6671	3.2	3.2	13.5	21.6	C49 H86 N13 O9
	1000.6736	-3.3	-3.3	2.5	45.2	C42 H95 N15 O4 I
	1000.6739	-3.6	-3.6	16.5	6.5	C58 H90 N5 O9
	1000.6739	-3.6	-3.6	20.5	211.6	C28 H13 O9 I4
	1000.6666	3.7	3.7	20.5	3.4	C64 H90 N O8
	1000.6664	3.9	3.9	6.5	27.3	C48 H95 N11 O3 I
	1000.6744	-4.1	-4.1	13.5	371.0	C13 H9 N12 O10 I4
	1000.6752	-4.9	-4.9	21.5	3.8	C59 H86 N9 O5
	1000.6752	-4.9	-4.9	25.5	186.4	C29 H9 N4 O5 I4
	1000.6653	5.0	5.0	30.5	161.1	C31 H5 N6 O2 I4

HRMs spectrum of compound **1d**

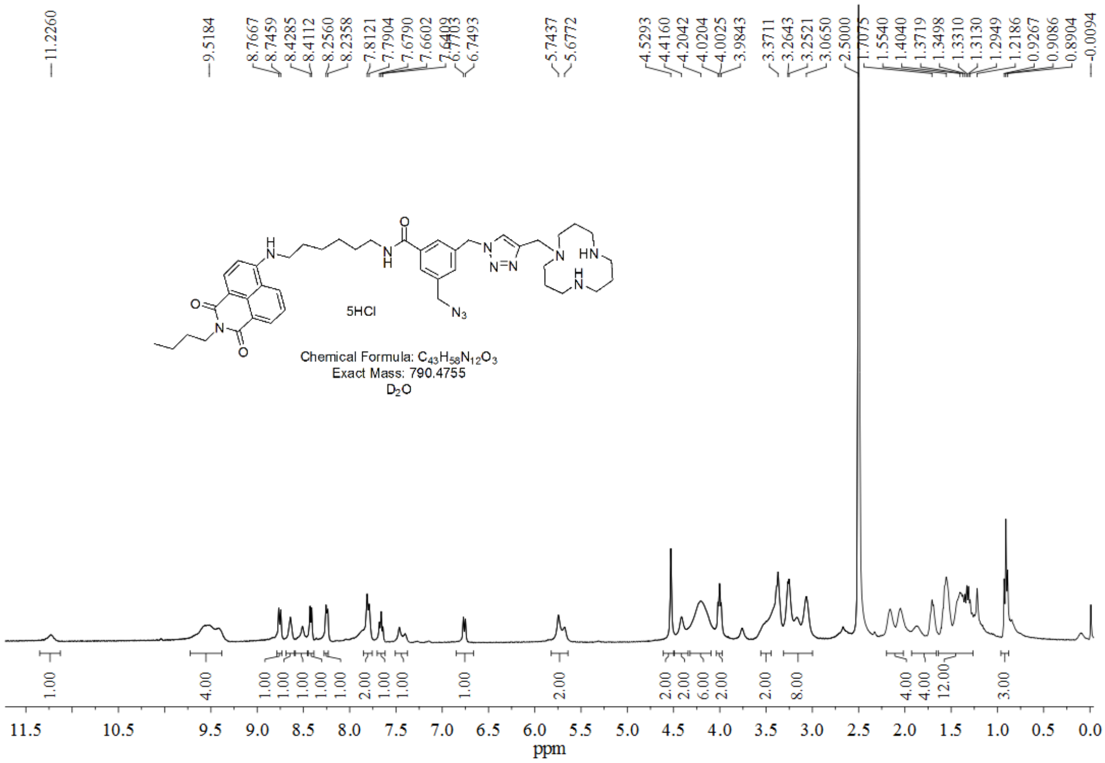

^{1}H NMR spectrum of compound **1e** (solvent: D_2O)

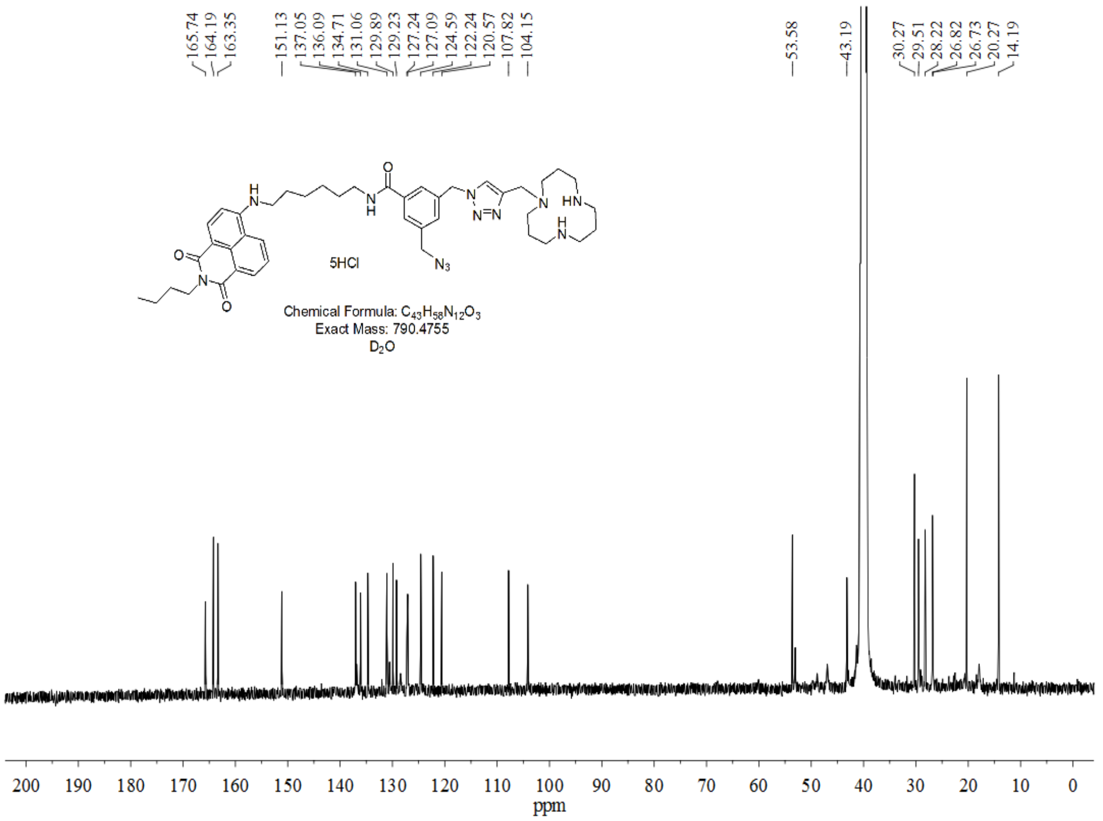

^{13}C NMR spectrum of compound **1e** (solvent: D_2O)

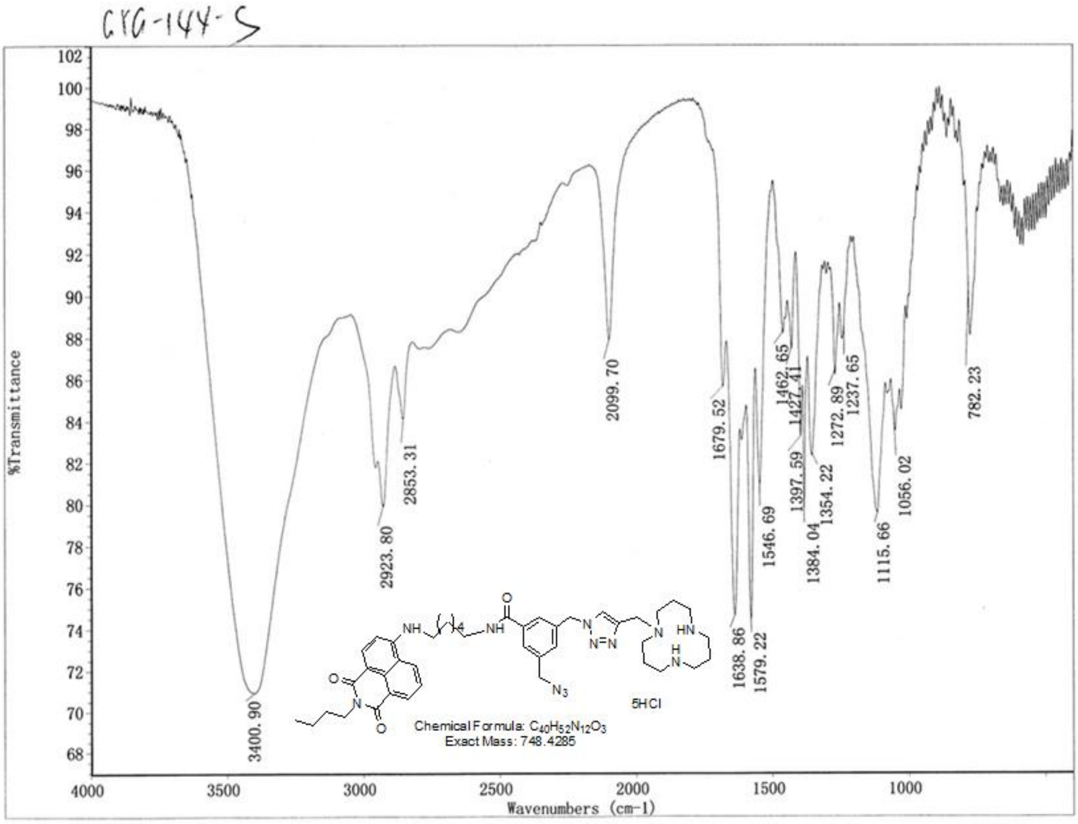

IR spectrum of compound **1e**

Elemental Composition Report **Page 1**

Single Mass Analysis
Tolerance = 5.0 PPM / DBE: min = -1.5, max = 50.0
Element prediction: Off
Number of isotope peaks used for i-FIT = 2

Monoisotopic Mass, Even Electron Ions
4915 formula(e) evaluated with 11 results within limits (up to 50 closest results for each mass)
Elements Used:
C: 0-100 H: 0-120 N: 0-15 O: 0-10 I: 0-4

GYG-144S 25 (0.462)
TOF MS ES+

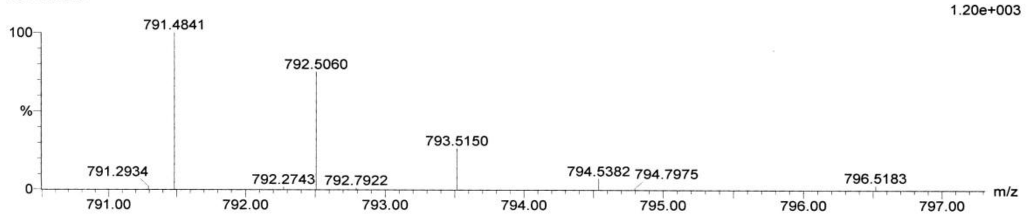

Minimum:				-1.5		
Maximum:		5.0	5.0	50.0		

Mass	Calc. Mass	mDa	PPM	DBE	i-FIT	Formula
791.4841	791.4839	0.2	0.3	7.5	34.4	C45 H76 O3 I
	791.4844	-0.3	-0.4	0.5	87.1	C30 H72 N12 O4 I
	791.4847	-0.6	-0.8	14.5	28.6	C46 H67 N2 O9
	791.4833	0.8	1.0	20.5	28.9	C43 H59 N12 O3
	791.4852	-1.1	-1.4	7.5	76.8	C31 H63 N14 O10
	791.4828	1.3	1.6	27.5	6.1	C58 H63 O2
	791.4860	-1.9	-2.4	19.5	22.3	C47 H63 N6 O5
	791.4820	2.1	2.7	15.5	36.2	C42 H63 N8 O7
	791.4812	2.9	3.7	8.5	42.9	C41 H72 N6 O I
	791.4871	-3.0	-3.8	-0.5	74.3	C34 H76 N6 O6 I
	791.4873	-3.2	-4.0	24.5	16.9	C48 H59 N10 O

HRMs spectrum of compound **1e**
